# Supplementary material for: Does co-inoculation of mycorrhiza and Piriformospora indica fungi enhance the efficiency of chlorophyll fluorescence and essential oil composition in peppermint under irrigation with saline water from the Caspian Sea?
Source: PLoS One. 2021 Jul 9;16(7):e0254076. doi: 10.1371/journal.pone.0254076 (PMC8270468; doi:10.1371/journal.pone.0254076)
Supplement: S1 Table — (DOCX) [file pone.0254076.s001.docx]

| fo | | YII | | FV/fm | | Na^+^ (meq/gdw^-1^) | | Essential oil (%) | | Stomatal conductance  (mmol (H_2_O) m^–2^ s^–1^) | | Membrane electrolyte leakage % | | Inoculation treatment | Salt treatment (dsm-^1^) |
| --- | --- | --- | --- | --- | --- | --- | --- | --- | --- | --- | --- | --- | --- | --- | --- |
| Mean | SD | Mean | SD | Mean | SD | Mean | SD | Mean | SD | Mean | SD | Mean | SD |  |  |
| 1.060 | 0.021 | 0.680 | 0.000 | 0.845 | 0.006 | 0.187 | 0.009 | 2.053 | 0.070 | 16.597 | 0.332 | 20.170 | 0.520 | Non-inoculated treatment | 0 |
| 1.050 | 0.012 | 0.712 | 0.004 | 0.849 | 0.001 | 0.130 | 0.012 | 2.663 | 0.035 | 19.837 | 0.203 | 19.103 | 0.743 | *P. indica* | 0 |
| 1.053 | 0.018 | 0.703 | 0.015 | 0.850 | 0.003 | 0.160 | 0.021 | 2.580 | 0.023 | 19.010 | 0.176 | 18.133 | 0.602 | AMF | 0 |
| 1.037 | 0.007 | 0.730 | 0.012 | 0.852 | 0.002 | 0.145 | 0.025 | 2.697 | 0.033 | 20.360 | 0.090 | 19.033 | 0.191 | *P. indica** AMF | 0 |
| 1.233 | 0.015 | 0.540 | 0.006 | 0.784 | 0.005 | 0.313 | 0.027 | 1.977 | 0.003 | 15.947 | 0.107 | 29.153 | 0.229 | Non-inoculated treatment | 3 |
| 1.153 | 0.007 | 0.603 | 0.018 | 0.823 | 0.010 | 0.233 | 0.015 | 2.523 | 0.035 | 18.933 | 0.348 | 21.203 | 0.455 | P. indica | 3 |
| 1.180 | 0.015 | 0.613 | 0.015 | 0.820 | 0.008 | 0.267 | 0.035 | 2.490 | 0.026 | 18.890 | 0.036 | 22.913 | 0.248 | AMF | 3 |
| 1.117 | 0.003 | 0.643 | 0.003 | 0.818 | 0.014 | 0.210 | 0.015 | 2.613 | 0.009 | 19.913 | 0.158 | 22.167 | 0.548 | *P. indica** AMF | 3 |
| 1.510 | 0.029 | 0.473 | 0.003 | 0.699 | 0.004 | 0.883 | 0.050 | 0.867 | 0.019 | 5.460 | 0.329 | 55.080 | 2.287 | Non-inoculated treatment | 6 |
| 1.370 | 0.035 | 0.521 | 0.015 | 0.739 | 0.011 | 0.670 | 0.035 | 1.193 | 0.027 | 6.983 | 0.035 | 46.070 | 1.191 | *P. indica* | 6 |
| 1.410 | 0.006 | 0.493 | 0.019 | 0.721 | 0.002 | 0.660 | 0.015 | 1.500 | 0.015 | 7.430 | 0.300 | 48.600 | 1.108 | AMF | 6 |
| 1.320 | 0.012 | 0.593 | 0.030 | 0.740 | 0.002 | 0.763 | 0.027 | 1.660 | 0.081 | 8.617 | 0.509 | 45.843 | 2.465 | *P. indica** AMF | 6 |
| 1.730 | 0.042 | 0.410 | 0.012 | 0.611 | 0.010 | 1.293 | 0.103 | 0.817 | 0.111 | 2.620 | 0.221 | 68.147 | 0.254 | Non-inoculated treatment | 9 |
| 1.550 | 0.015 | 0.453 | 0.009 | 0.684 | 0.004 | 1.043 | 0.047 | 1.067 | 0.020 | 5.033 | 0.052 | 60.260 | 1.708 | *P. indica* | 9 |
| 1.570 | 0.053 | 0.443 | 0.015 | 0.671 | 0.012 | 0.963 | 0.084 | 1.027 | 0.050 | 5.680 | 0.098 | 59.953 | 3.549 | AMF | 9 |
