# Supplementary material for: Does co-inoculation of mycorrhiza and Piriformospora indica fungi enhance the efficiency of chlorophyll fluorescence and essential oil composition in peppermint under irrigation with saline water from the Caspian Sea?
Source: PLoS One. 2021 Jul 9;16(7):e0254076. doi: 10.1371/journal.pone.0254076 (PMC8270468; doi:10.1371/journal.pone.0254076)
Supplement: S2 Table — (DOCX) [file pone.0254076.s002.docx]

| Salt treatment (dsm^-1^) | fm | | fv | | Chlorophyll a (µg/ml) | | Chlorophyll b (µg/ml) | | Y(NPQ) | | Y(NO) | | P  (meq/gdw^-1^) | | K^+^  (meq/gdw^-1^) | | NPQ | | ETR | |
| --- | --- | --- | --- | --- | --- | --- | --- | --- | --- | --- | --- | --- | --- | --- | --- | --- | --- | --- | --- | --- |
|  | Mean | SD | Mean | SD | Mean | SD | Mean | SD | Mean | SD | Mean | SD | Mean | SD | Mean | SD | Mean | SD | Mean | SD |
| 0 | 6.960 | 0.046 | 5.910 | 0.048 | 6.816 | 0.136 | 5.733 | 0.132 | 0.114 | 0.003 | 0.140 | 0.001 | 0.447 | 0.027 | 1.800 | 0.038 | 0.814 | 0.015 | 21.083 | 0.621 |
| 3 | 6.268 | 0.177 | 5.097 | 0.181 | 6.198 | 0.140 | 5.305 | 0.129 | 0.132 | 0.008 | 0.156 | 0.004 | 0.393 | 0.028 | 1.222 | 0.057 | 0.870 | 0.008 | 19.167 | 0.842 |
| 6 | 5.105 | 0.071 | 3.702 | 0.074 | 5.211 | 0.122 | 4.652 | 0.129 | 0.213 | 0.014 | 0.216 | 0.011 | 0.276 | 0.022 | 0.895 | 0.057 | 1.031 | 0.023 | 14.500 | 0.793 |
| 9 | 4.643 | 0.082 | 3.054 | 0.091 | 4.371 | 0.119 | 4.197 | 0.109 | 0.239 | 0.011 | 0.276 | 0.007 | 0.226 | 0.019 | 0.427 | 0.048 | 1.172 | 0.023 | 9.917 | 0.668 |
