# Supplementary material for: Does co-inoculation of mycorrhiza and Piriformospora indica fungi enhance the efficiency of chlorophyll fluorescence and essential oil composition in peppermint under irrigation with saline water from the Caspian Sea?
Source: PLoS One. 2021 Jul 9;16(7):e0254076. doi: 10.1371/journal.pone.0254076 (PMC8270468; doi:10.1371/journal.pone.0254076)
Supplement: S3 Table — (DOCX) [file pone.0254076.s003.docx]

| Inoculation treatment | fm | | fv | | Chlorophyll a (µg/ml) | | Chlorophyll b (µg/ml) | | Y(NPQ) | | Y(NO) | | P  (meq/gdw^-1^) | | K^+^  (meq/gdw^-1^) | | NPQ | | ETR | |
| --- | --- | --- | --- | --- | --- | --- | --- | --- | --- | --- | --- | --- | --- | --- | --- | --- | --- | --- | --- | --- |
|  | Mean | SD | Mean | SD | Mean | SD | Mean | SD | Mean | SD | Mean | SD | Mean | SD | Mean | SD | Mean | SD | Mean | SD |
| Non-inoculated treatment | 5.508 | 0.273 | 4.124 | 0.347 | 5.099 | 0.270 | 4.552 | 0.184 | 0.204 | 0.023 | 0.214 | 0.019 | 0.224 | 0.027 | 0.963 | 0.156 | 0.928 | 0.034 | 13.667 | 1.269 |
| *P. indica* | 5.927 | 0.278 | 4.646 | 0.332 | 5.808 | 0.295 | 5.069 | 0.182 | 0.161 | 0.014 | 0.206 | 0.018 | 0.388 | 0.036 | 1.188 | 0.140 | 0.960 | 0.045 | 15.917 | 1.545 |
| AMF | 5.860 | 0.300 | 4.557 | 0.357 | 5.708 | 0.297 | 5.052 | 0.231 | 0.175 | 0.019 | 0.187 | 0.016 | 0.343 | 0.027 | 1.175 | 0.168 | 0.987 | 0.048 | 17.167 | 1.424 |
| *P. indica** AMF | 5.681 | 0.317 | 4.436 | 0.369 | 5.982 | 0.310 | 5.215 | 0.215 | 0.158 | 0.013 | 0.179 | 0.015 | 0.387 | 0.029 | 1.018 | 0.160 | 1.012 | 0.051 | 17.917 | 1.422 |
