# Supplementary material for: Does co-inoculation of mycorrhiza and Piriformospora indica fungi enhance the efficiency of chlorophyll fluorescence and essential oil composition in peppermint under irrigation with saline water from the Caspian Sea?
Source: PLoS One. 2021 Jul 9;16(7):e0254076. doi: 10.1371/journal.pone.0254076 (PMC8270468; doi:10.1371/journal.pone.0254076)
Supplement: Attachment — Submitted filename: PONE-D-21-02576_R2 - with corrections.pdf [file pone.0254076.s008.pdf]

PLOS ONE

Does co-inoculation of mycorrhiza and Piriformospora indica fungi enhance the efficiency of Chlorophyll fluorescence and essential oil composition in peppermint under irrigation with saline water from the Caspian Sea?

--Manuscript Draft--

**Manuscript Number:**	PONE-D-21-02576R2
**Article Type:**	Research Article
**Full Title:**	Does co-inoculation of mycorrhiza and Piriformospora indica fungi enhance the efficiency of Chlorophyll fluorescence and essential oil composition in peppermint under irrigation with saline water from the Caspian Sea?
**Short Title:**	Endophytic fungi benefits on photosystem II and essential oil composition in peppermint under salinity stress
**Corresponding Author:**	masoumeh Khalvandi, Ph.D.<br>Shahrood University of Technology<br>shahrood, semnan IRAN, ISLAMIC REPUBLIC OF
**Keywords:**	Chlorophyll fluorescence; Secondary metabolites; Piriformospora indica; arbuscular mycorrhizal; salinity stress
**Abstract:**	Symbiotic associations between plants and fungi endophytes are ecologically important in medicinal and aromatic plants that highly affects the quantity and quality of herbal products. In this study a pot experiment was carried out in greenhouse to investigate the interactive effects of Piriformospora indica and arbuscular mycorrhizal inoculation on the chlorophyll fluorescence, essential oil composition and antioxidant enzymes of peppermint under saline condition. The results showed that Fo, YNPQ, YNO and NPQ values were obviously increased under salinity conditions, while essential oil content, chlorophyll a and b, gs, Fm, Fv, ETR, ΦPSII and Fv/Fm ratio decreased by increasing salinity. In addition, salt-induced the excess uptake of Na+, whereas the opposite trend was observed for P and K+. The synergistic association of P. indica and AMF caused to a considerable increase in the antioxidant ability, essential oil content, Fv/Fm ratio and ΦPSII, amount of P and K+ uptake in salt stressed plants. The main oil constituents, menthol, menthone and 1,8-cineole increased considerably in inoculated plants. On the other hand, the applied fungi endophytes positively raised the ability of peppermint to overcome the negative effect of the salinity stress.
**Order of Authors:**	masoumeh Khalvandi, Ph.D.
	Mohammadreza Amerian
	Hematollah Pirdashti
	Sara Keramati
**Response to Reviewers:**	Prof. Mayank Gururani<br>Editor-in-Chief<br>PLOS ONE Journal<br>Dear Prof., Mayank Gururani<br>Attached please find our revised manuscript entitled: Does co-inoculation of mycorrhiza and Piriformospora indica fungi enhance the efficiency of Chlorophyll fluorescence and essential oil composition in peppermint under irrigation with saline water from the Caspian Sea? (No.: PONE-D-21-02576) for publication in PLOS ONE Journal.<br>Thank you for giving us this opportunity to revise and resubmit our manuscript. We appreciate your comments and the suggestions made by referees to improve the manuscript. We responded to all of the reviewers' suggestions.<br>We hope that after these enhancements the manuscript can now be accepted for publication; however, we are certainly willing to consider further changes if necessary.<br>We look forward to hearing from you.

Powered by Editorial Manager® and ProduXion Manager® from Aries Systems Corporation

Yours faithfully,
Masoumeh Khalvandi
In behalf authors

Response to Review Comments

(Manuscript Number: PONE-D-21-02576)

Response to Reviewer #1 Comments
Reviewer point #1: If they limit the discussion to their actual findings, the entire discussion can be summarized to 1/3rd of what is presented.
Author response #1: The length of the discussion part has been reduced. And these sentences were removed in the discussion part.
•The reason that salinity decreased fungal colonization might be attributed to the adverse effects of salinity on photosynthesis, along with a reduction in carbon supply from plant to endophytic fungus. This can also be supported by the fact that salinity induced changes in morphological features of fungal hyphae. Besides,
•These results are similar to what has been recorded in wheat (lyas et al, 2020), and maize (Wang et al, 2020).
•inhibition of QA re-oxidation, and degradation of D1 and D2 proteins in the PSII which
•Salinity stress probably saturates the electron transport chain that leads to accumulation of protons, followed by an increase in the amount of NPQ. The increase in NPQ, which is actually an indicator of heat loss in the processes of the excessive energy removal, shows the high capacity of the xanthophyll cycle (Nilkens et al., 2010; Murchie and Lawson, 2013).
•Higher values of photochemical activity of PSII in plants which have a symbiotic relationship with fungi are mainly associated with: the increased density of photosynthetic units, the improved reaction center activity, enhancing the plants' ability to use light energy, and facilitating the electron transport to NADP (Xu et al, 2016; Moreira et al. 2015; Zhang et al, 2018; Shahabivand et al, 2017; Ghorbani et al, 2018; Shahabivand et al, 2012). Furthermore, microbial inoculation can alleviate the adverse impacts of salinity on the photochemical activity of PSII through expression of the genes encoding D1 and D2 proteins, improving water absorption, modulating membrane damage, and improving chlorophyll content (Chen et al, 2017).
•AMF has been reported to regulate root permeability by doubling the expression of the LSPLP1 gene, the strategy which leads to a higher tolerance to osmotic stress) (Heikham Evelin et al, 2019).
•In addition to what is mentioned above, salinity suppresses the biosynthesis of essential oil. The reason for the reduction in the synthesis of essential oils can be related to the low availability of assimilates as a result of reduced photosynthetic activity and inactivation of the PSII reaction center (Zuo et al, 2017). However, even when photosynthesis assimilation approaches zero (due to stomatal restriction, damage to chlorophyll, and chloroplast membrane system under severe stress conditions) monoterpenes biosynthesis still occurs due to alternative carbon sources allocation (Lavoir et al, 2009). Naturally, monoterpenes are biosynthesized through MEP/ DOXP pathway and are fueled by a constant supply of substrates, high amounts of acetyl-CoA, and ATP and NADPH+H cofactors (Niinemets et al., 2002, Behn et al, 2010). This can be the reason for the tight link between methyl erythritol-4-osphate

(MEP) and photosynthesis, as the Calvin cycle in chloroplasts is the main carbon sources for terpenoid biosynthesis and the MEP pathway is a place for reducing excess electron flow in the photosynthetic electron transfer chain (Valifard et al, 2018).
•It is believed that the monoterpene pattern is mostly determined by NADPH+H+ - dependent reductive interconversions, like a reduction of menthone to menthol (Behn et al, 2010). These modifications in the biosynthesis of monoterpenes can be linked to alterations in the photosynthetic electron transfer chain, the production of photosynthetic NDPH2 (Maffei and Codignola, 1990), and oxidation of monoterpenes by ROS to generate oxygenated forms under environmental stress (Zuo et al, 2017).

These references were also removed in the revised manuscript.

1.Agron. 10, 160–162.Wu, M., Wei, Q., Xu, L., Li, H., Oelmüller, R., & Zhang, W. (2018). Piriformospora indica enhances phosphorus absorption by stimulating acid phosphatase activities and organic acid accumulation in Brassica napus. Plant and Soil, 432(1-2), 333-344.
2.Behn, H., Albert, A., Marx, F., Noga, G., & Ulbrich, A. (2010). Ultraviolet-B and photosynthetically active radiation interactively affect yield and pattern of monoterpenes in leaves of peppermint (Mentha× piperita L.). Journal of agricultural and food chemistry, 58(12), 7361-7367.
3.Flexas, J., Escalona, J. M., & Medrano, H. (1999). Water stress induces different levels of photosynthesis and electron transport rate regulation in grapevines. Plant, Cell & Environment, 22(1), 39-48.
4.lyas, N., Mazhar, R., Yasmin, H., Khan, W., Iqbal, S., Enshasy, H. E., & Dailin, D. J. (2020). Rhizobacteria isolated from saline soil induce systemic tolerance in wheat (Triticum aestivum L.) against salinity stress. Agronomy, 10(7), 989.
5.Lavoir, A. V., Staudt, M., Schnitzler, J. P., Landais, D., Massol, F., Rocheteau, A., ... & Rambal, S. (2009). Drought reduced monoterpene emissions from the evergreen Mediterranean oak Quercus ilex: results from a throughfall displacement experiment. Biogeosciences, 6(7), 1167-1180.
6.Maffei, M., & Codignola, A. (1990). Photosynthesis, photorespiration and herbicide effect on terpene production in peppermint (Mentha piperita L.). Journal of Essential Oil Research, 2(6), 275-286.
7.Sebastian, A., & Prasad, M. N. V. (2019). Photosynthetic light reactions in Oryza sativa L. under Cd stress: Influence of iron, calcium, and zinc supplements. The EuroBiotech Journal. 3(4): 175-181.
8.Zuo, Z., Wang, B., Ying, B., Zhou, L., & Zhang, R. (2017). Monoterpene emissions contribute to thermotolerance in Cinnamomum camphora. Trees, 31(6), 1759-1771.

Reviewer point #2: Abstract: First sentence of the abstracts seems complex and needs to be simplified
Author response #2: The correction was done in Abstract.

Reviewer point #3: Introduction – 2nd paragraph – 3rd line: ‘Photosynthesis process’ – please delete ‘process’
Author response #3: process was deleted in ‘Photosynthesis process’

Reviewer point #4: Introduction – 4th paragraph – Line No.12: ‘which are all pharmaceutical useful’ – please modify as ‘pharmaceutically useful’
Author response #4: ‘which are all pharmaceutical useful’ was modified to ‘pharmaceutically useful’

Reviewer point #5: Material and Methods: 1st paragraph – 2nd line: ‘This research was carried out in a factorial experiment in a….’ – may be modified as: ‘A factorial experiment was carried out in a....’
Author response #5: ‘This research was carried out in a factorial experiment in a….’ was modified to ‘A factorial experiment was carried out in a....’

Reviewer point #6: Discussion – 9th paragraph: ‘Several reports have reported a direct correlation between nutrient imbalance and the simultaneous decline in PSII function in salt-stressed plants’ - the sentence may be modified as ‘Several reports suggest a direct correlation between nutrient imbalance and the simultaneous decline in PSII function in salt-stressed plants’
Author response #6: The correction has been made in the revised manuscript.

Powered by Editorial Manager® and ProduXion Manager® from Aries Systems Corporation

Reviewer point #7: which are these reports? Please refer to the reports; the reference of Yang et al. (2021) is not sufficient to substantiate the argument of ‘several reports’; otherwise, modify the sentence.
Author response #7: Thank you for pointing this out. The following articles were added to the references.
•Loudari, A., Benadis, C., Naciri, R., Soulaimani, A., Zeroual, Y., Gharous, M. E., Kalaji, H M., & Oukarroum, A. (2020). Salt stress affects mineral nutrition in shoots and roots and chlorophyll a fluorescence of tomato plants grown in hydroponic culture. Journal of Plant Interactions, 15(1), 398-405.
•Qu, C., Liu, C., Gong, X., Li, C., Hong, M., Wang, L., & Hong, F. (2012). Impairment of maize seedling photosynthesis caused by a combination of potassium deficiency and salt stress. Environmental and Experimental Botany, 75, 134-141

Reviewer point #8: 9th paragraph – line 7: ‘fungi symbiosis’ – may be modified as fungal symbiosis
Author response #8: ‘fungi symbiosis’ was modified to ‘fungal symbiosis’

Reviewer point #9: 9th paragraph: lines 6-7: ‘Nevertheless, endophytic fungi symbiosis mitigated the inhibitory effect of salt stress on the mineral uptake’ – please mention the evidence which the authors have presented as results in their study to substantiate this argument; otherwise delete this speculative argument. (the first sentence of this paragraph is ‘The results of this study showed that severe salinity stress remarkably reduced P and K+ uptake because of the high rate of Na+ absorption’, is contradictory to this argument.
Author response #9: ‘Nevertheless, endophytic fungi symbiosis mitigated the inhibitory effect of salt stress on the mineral uptake’ was modified to ‘Nevertheless, in inoculated plant endophytic fungal symbiosis increased P and K+ content (Figure. 6a) and decreased Na+ uptake in peppermint leaves (Figure. 4c).’
In order to avoid ambiguity and contradiction, ‘in non-inoculated plant’ and ‘in inoculated plant’ were added to the text of this paragraph.

Reviewer point #10: Line 11: ‘osmotic stress)’ – delete the bracket
Author response #10: done

Reviewer point #11: 10th paragraph: lines 5-7: ‘In agreement with the present results, Xu et al. (2016) reported that the positive effect of AMF on Fv/Fm increasing in maize seedlings can be attributed to the absorption of mineral nutrients, activating mediated genes and sink stimulation’ – how can it be in agreement with, especially when the authors argue in the 9th paragraph that ‘The results of this study showed that severe salinity stress remarkably reduced P and K+ uptake because of the high rate of Na+ absorption’ ? - please note the contradiction and revise
Author response #11:
In order to avoid ambiguity and contradiction, ‘The results of this study showed that severe salinity stress remarkably reduced P and K+ uptake because of the high rate of Na+ absorption’ was modified to ‘The results of this study showed that severe salinity stress remarkably reduced P and K+ uptake in non-inoculated plant because of the high rate of Na+ absorption.’

Thank you for your valuable suggestion. After reviewing articles in various journals, we found that they also reported such contradictions, and in all of them, although the rate of colonization and formation of fungal hyphae and spores decreased under salinity stress, its presence in the plant roots was able to reduce the negative effects of salinity stress on the plant and improves the amount of some plant parameters.
•Klinsukon, C., Lumyong, S., Kuyper, T. W., & Boonlue, S. (2021). Colonization by arbuscular mycorrhizal fungi improves salinity tolerance of eucalyptus (Eucalyptus camaldulensis) seedlings. Scientific Reports, 11(1), 1-10.

•Zai, X. M., Fan, J. J., Hao, Z. P., Liu, X. M., & Zhang, W. X. (2021). Effect of co-inoculation with arbuscular mycorrhizal fungi and phosphate solubilizing fungi on nutrient uptake and photosynthesis of beach palm under salt stress environment. Scientific Reports, 11(1), 1-11.

Powered by Editorial Manager® and ProduXion Manager® from Aries Systems Corporation

	•Parvin, S., Van Geel, M., Yeasmin, T., Verbruggen, E., & Honnay, O. (2020). Effects of single and multiple species inocula of arbuscular mycorrhizal fungi on the salinity tolerance of a Bangladeshi rice (Oryza sativa L.) cultivar. Mycorrhiza, 30(4), 431-444.<br><br>•Kaya, C., Ashraf, M., Sonmez, O., Aydemir, S., Tuna, A. L., & Cullu, M. A. (2009). The influence of arbuscular mycorrhizal colonisation on key growth parameters and fruit yield of pepper plants grown at high salinity. Scientia horticulturae, 121(1), 1-6.<br><br>•Jahromi, F., Aroca, R., Porcel, R., & Ruiz-Lozano, J. M. (2008). Influence of salinity on the in vitro development of Glomus intraradices and on the in vivo physiological and molecular responses of mycorrhizal lettuce plants. Microbial Ecology, 55(1), 45.<br><br>Reviewer point #12: I think the 11th and 12th paragraph are unnecessary as these do not discuss any specific findings of the researchers.<br>Author response #12: These 11th and 12th paragraphs was delated in the revised manuscript.
Additional Information:	
Question	Response
**Financial Disclosure**<br><br>Enter a financial disclosure statement that describes the sources of funding for the work included in this submission. Review the submission guidelines for detailed requirements. View published research articles from *PLOS ONE* for specific examples.<br><br>This statement is required for submission and **will appear in the published article** if the submission is accepted. Please make sure it is accurate.<br><br>**Unfunded studies**<br>Enter: *The author(s) received no specific funding for this work.*<br><br>**Funded studies**<br>Enter a statement with the following details:<br>• Initials of the authors who received each award<br>• Grant numbers awarded to each author<br>• The full name of each funder<br>• URL of each funder website<br>• Did the sponsors or funders play any role in the study design, data collection and analysis, decision to publish, or preparation of the manuscript?<br>• **NO** - Include this sentence at the end of your statement: *The funders had no role in study design, data collection and analysis, decision to publish, or preparation of the manuscript.*<br>• **YES** - Specify the role(s) played.	there was no fund on the article.<br>The funders had no role in study design, data collection and analysis, decision to publish, or preparation of the manuscript.

* typeset	
**Competing Interests**<br><br>Use the instructions below to enter a competing interest statement for this submission. On behalf of all authors, disclose any competing interests that could be perceived to bias this work—acknowledging all financial support and any other relevant financial or non-financial competing interests.<br><br>This statement is required for submission and **will appear in the published article** if the submission is accepted. Please make sure it is accurate and that any funding sources listed in your Funding Information later in the submission form are also declared in your Financial Disclosure statement.<br><br>View published research articles from *PLOS ONE* for specific examples.<br><br>**NO authors have competing interests**<br><br>Enter: *The authors have declared that no competing interests exist.*<br><br>**Authors with competing interests**<br><br>Enter competing interest details beginning with this statement:<br><br>*I have read the journal's policy and the authors of this manuscript have the following competing interests: [insert competing interests here]*<br><br>* typeset	The authors have declared that no competing interests exist.
**Ethics Statement**<br><br>Enter an ethics statement for this submission. This statement is required if the study involved:	N/A

• Human participants<br>• Human specimens or tissue<br>• Vertebrate animals or cephalopods<br>• Vertebrate embryos or tissues<br>• Field research<br><br>Write "N/A" if the submission does not require an ethics statement.<br><br>General guidance is provided below. Consult the submission guidelines for detailed instructions. **Make sure that all information entered here is included in the Methods section of the manuscript.**	

**Format for specific study types**<br><br>**Human Subject Research (involving human participants and/or tissue)**<br>• Give the name of the institutional review board or ethics committee that approved the study<br>• Include the approval number and/or a statement indicating approval of this research<br>• Indicate the form of consent obtained (written/oral) or the reason that consent was not obtained (e.g. the data were analyzed anonymously)<br><br>**Animal Research (involving vertebrate animals, embryos or tissues)**<br>• Provide the name of the Institutional Animal Care and Use Committee (IACUC) or other relevant ethics board that reviewed the study protocol, and indicate whether they approved this research or granted a formal waiver of ethical approval<br>• Include an approval number if one was obtained<br>• If the study involved *non-human primates*, add *additional details* about animal welfare and steps taken to ameliorate suffering<br>• If anesthesia, euthanasia, or any kind of animal sacrifice is part of the study, include briefly which substances and/or methods were applied<br><br>**Field Research**<br><br>Include the following details if this study involves the collection of plant, animal, or other materials from a natural setting:<br>• Field permit number<br>• Name of the institution or relevant body that granted permission	
**Data Availability**<br><br>Authors are required to make all data underlying the findings described fully available, without restriction, and from the time of publication. PLOS allows rare exceptions to address legal and ethical concerns. See the PLOS Data Policy and FAQ for detailed information.	Yes - all data are fully available without restriction

A Data Availability Statement describing where the data can be found is required at submission. Your answers to this question constitute the Data Availability Statement and **will be published in the article**, if accepted.<br><br>**Important:** Stating 'data available on request from the author' is not sufficient. If your data are only available upon request, select 'No' for the first question and explain your exceptional situation in the text box.<br><br>Do the authors confirm that all data underlying the findings described in their manuscript are fully available without restriction?	
**Describe where the data may be found in full sentences. If you are copying our sample text, replace any instances of XXX with the appropriate details.**<br><br>• If the data are **held or will be held in a public repository**, include URLs, accession numbers or DOIs. If this information will only be available after acceptance, indicate this by ticking the box below. For example: *All XXX files are available from the XXX database (accession number(s) XXX, XXX.).*<br>• If the data are all contained **within the manuscript and/or Supporting Information files**, enter the following: *All relevant data are within the manuscript and its Supporting Information files.*<br>• If neither of these applies but you are able to provide **details of access elsewhere**, with or without limitations, please do so. For example:<br><br>*Data cannot be shared publicly because of [XXX]. Data are available from the XXX Institutional Data Access / Ethics Committee (contact via XXX) for researchers who meet the criteria for access to confidential data.*<br><br>*The data underlying the results presented in the study are available from (include the name of the third party*	All relevant data are within the manuscript and its Supporting Information files

and contact information or URL).<br>• This text is appropriate if the data are owned by a third party and authors do not have permission to share the data.<br><br>* typeset	
Additional data availability information:	

Manuscript Click here to access/download;Manuscript;Manuscript.docx

Does co-inoculation of mycorrhiza and *Piriformospora indica* fungi enhance the efficiency of Chlorophyll fluorescence and essential oil composition in peppermint under irrigation with saline water from the Caspian Sea?

Masoumeh Khalvandi[1*], Mohammadreza Amerian[2], Hematollah Pirdashti[3], Sara Keramati[3]

[1] Ph.D. Graduate, Department of Agronomy, Faculty of Agriculture, Shahrood University of Technology, Iran.

[2] Department of Agronomy, Faculty of Agriculture, Shahrood University of Technology, Iran

[3] Department of Agronomy, Genetic and Agricultural Biotechnology Institute of Tabarestan, Sari Agricultural Sciences and Natural Resources University, Iran

[*] Corresponding author
E-mail: M.khalvandi@gmail.com

Abstract

Symbiotic associations with endophytic fungi are ecologically important for medicinal and aromatic plants. Endophytic fungi highly affect the quantity and quality of herbal products. In this study, a pot experiment was carried out in the greenhouse to investigate the interactive effects of *Piriformospora indica* and arbuscular mycorrhizal (AMF) inoculation on the chlorophyll fluorescence, essential oil composition, and antioxidant enzymes of peppermint under saline condition. The results showed that Fo, YNPQ, YNO, and NPQ values were obviously increased under salinity conditions, while essential oil content, chlorophyll a and b, gs, Fm, Fv, ETR, ΦPSII and Fv/Fm ratio decreased by increasing salinity. In addition, salt induced the excess Na^+ uptake, whereas the opposite trend was observed for P and K^+. The synergistic association of *P. indica* and AMF caused a considerable increase in the antioxidant ability, essential oil content, Fv/Fm ratio, ΦPSII, and amount of P and K^+ uptake in salt-stressed plants. The main peppermint oil constituents, menthol, menthone, and 1,8-cineole increased considerably in inoculated plants. Besides, the applied endophytic fungi positively enhanced the ability of peppermint to alleviate the negative effect of the salinity stress.

Keywords: Chlorophyll fluorescence, secondary metabolites, *Piriformospora indica*, arbuscular mycorrhizal, salinity stress.

1. Introduction

Mentha piperita is one of the most important medicinal plants of Lamiaceae family, the demand of which has grown dramatically in recent decades. The essential oil of peppermint is widely used in food, fragrance, pharmaceutical and cosmetic industries (Hemming, 2013; Dolatabadi et al, 2012). It is evident that the essential oil contains a high proportion of cytotoxic, antioxidant, and anti-microbial compounds with health-beneficial effects such as antifungal effects, cancer prevention activities as well as scavenging of reactive oxygen species (ROS) activity because of its valuable constituents (Miguel, 2011; Moghaddam et al., 2013; Sun et al., 2014).

The sustainability of medicinal plants' yield is negatively affected by salt stress in many parts of the world (Abdelshafy Mohamad et al, 2020). Salinity causes a broad range of physiological changes in plants, induces impairments of metabolic processes, such as photosynthesis, pigment synthesis, secondary metabolite accumulation, destruction of the chloroplast and thylakoid systems, and inhibits the photosystem II (PSII) activity, which results in the excess accumulation of ROS (Ashraf et al., 2018; Monetti et al., 2014). In this regard, chlorophyll fluorescence is widely used to detect plant physiological status and to determine photosynthetic damage under various stresses, because the same can show the integrity of the thylakoid membrane, the quantum yield of photosystem II (PSII), and the balance between the metabolism process and energy production (Lu and Zhang, 1998; Giardi et al, 1996). It has been proved that salinity has a negative effect on the photochemical efficiency of PSII as shown by chlorophyll fluorescence parameters such as qP, ΦPSII, and Fv/Fm (Aroca et al, 2013; Radić et al., 2013). In this regard, previous research showed that salt stress reduced the quantum yield of photosystem II; whereas, it increased non-photochemical extinction in lettuce plants (Aroca et al, 2013), and decreased the ratio of the quantum yield of actual PSII photochemistry in durum wheat (Yaghoubi Khanghahi et al, 2020).

Monoterpenes play an important role in plants' response to environmental stresses. Nevertheless, there are few studies focusing on the relationship between terpene biosynthesis and the efficiency of PSII. Terpenoid biosynthesis patterns are mostly affected by photosynthetic carbon assimilation and partly regulated by

environmental stresses (Valifard et al, 2018; Haberstroh et al, 2018). There are also some reports about the anti-stress activity of monoterpenes in plants under stressful conditions. For example, it has been reported that the Monoterpenes in *Quercus ilex* can improve membrane integrity by ROS-scavenging, photoprotective, and thermotolerance roles (Peñuelas & Llusià., 2002, Loreto et al, 2002).

Applying Plant beneficial rhizospheric microorganisms (PBRMs) like symbiotic fungi is an important environmental strategy to overcome the deleterious effects of salinity stress, and to improve plants' yield and performance. Arbuscular mycorrhizal fungi and *Piriformospora indica* (also named as *Sebacinales indica*) are two of these beneficial endophytic fungi which increase plant resistance to various environmental stresses through a wide range of mechanisms; mechanisms such as stimulating the immune system of plants (Chen et al, 2017; Khalvandi et al, 2019), promoting nutrient uptake (Wu et al., 2018), and stimulating root growth (Mansotra et al., 2015). It is believed that symbiotic relationship with *P. indica* can help to ameliorate photo-oxidative damages to PSII and electron transfer chain (Yun et al, 2018), also, it increases the synthesis of secondary metabolites in various plants (Zolfaghari et al., 2013; Kari Dolatabadi et al., 2011). Several studies have shown that symbiotic relationship with *P. indica* can stimulate the human health-promoting compounds synthesis in various plants. For example: podophyllotoxins in *Linum album*, thymol and carvacrol in *Thymus vulgaris*, menthol in *Mentha piperita* (Dolatabadi et al, 2017), and estragole in *Ocimum basilicum*, pharmaceutically useful compounds (Baldi et al., 2010; Tarraf et al., 2015; Keramati et al, 2016). Moreover, it can reduce undesirable compounds for human nutrition, such as erucic acid and glucosinolates contents in *Brassica napus* (Su et al., 2017).

The present study aims to investigate the hypothesis that co-inoculation of AMF and *P. indica* can protect peppermint plants against adverse effects of the salt stress through modifying some physio-biochemical characteristics.

2. Materials and Methods

Growth Conditions:

Plants were grown in the greenhouse at Genetics and Agricultural Biotechnology Institute of Tabarestan at Sari Agricultural Sciences and Natural Resources University. A factorial experiment was carried out in a completely randomized design with three replications. Plants were treated with four levels of fungal inoculation including no-inoculation (control), *Piriformospora indica* (*P. indica*), Arbuscular mycorrhizal fungi (AMF), and co-inoculation with *P. indica* and AMF (*P. indica* + AMF) under four salinity levels including 0, 3, 6 and 9 dSm^{-1} (the Caspian Sea water and distilled water mixture was used for irrigation) (Table 1). In order to reach each level of salinity stress: determined amount of the Caspian Sea water and distilled water were mixed and then the desired salinity was determined by EC meter. *P. indica* and AMF were prepared from Sari Agricultural Sciences and Natural Resources University, Sari, Iran. 10 g of Arbuscular mycorrhizal inoculum (with a density of 120 active spores per gram) and 10 ml of *Piriformospora indica* suspension (1×10^9) were added to the pots. Peppermint rhizomes were planted in plastic pots, and were irrigated with saline water after four weeks of planting. Plant physiological measurements and sampling for the desired traits were carried out after two months of planting in all plants. The roots were bleached in a solution of KOH 10%, they were painted, using 5% solution of ink and vinegar. Then, 40 pieces of stained root were spread out in a Petri dish. The colonization percentage was measured by using the gridline intersects method optical microscope (10- 40X) (Vierheilig et al., 1998; Norris et al., 1992; Varma et al., 1999).

Physiological parameters:

Chlorophyll fluorescence parameters were measured in the last fully developed leaf by using pulse amplitude modulated fluorometer (PAM-2500, Walz, Germany). First, leaves were placed in darkness for 30 minutes using specific leaf clamps. The samples were exposed to low-intensity light [< 0.1 μmol (photon) m−2 s−1, red light]. Then, a saturating light pulse [> 8,000 μmol (photon) m−2 s−1, white light) was turned on for 1 s (one pulse). The minimum fluorescence (Fo) and maximum fluorescence (Fm) were determined in dark-adapted leaves. The variable fluorescence (Fv) and maximum quantum photosystem II efficiency (Fv/Fm) were evaluated based on Equations 1 and 2. and the effective photochemical quantum efficiency II [ΦPSII], electron transfer rate [ETR, μmol(electron) m–2 s–1), non-photochemical quenching (NPQ), the quantum yield of regulated energy dissipation (YNPQ), and Quantum yield of non-regulated energy dissipation in PSII (Y (NO)) were calculated by using Equations 3 and 6 (Khanghahi et al, 2012).

1) Fv= Fm- Fo

2) Fv/Fm= (Fm- Fo)/ Fm

3) ΦPSII= (Fm′ − F)/Fm′

4) NPQ = (Fm/Fm') – 1

5) Y(NPQ) = (F/Fm') – (F/Fm)

6) Y(NO) = F/Fm

F= steady-state fluorescenc

Fm'= maximum fluorescence measured in light-exposed leaf samples.

Stomatal conductance (gs) was determined by using a porometer data. Chlorophyll a and b concentrations were calculated based on the Arnon's methods (1949).
The minerals Na^+ and K^+, P in peppermint leaves were determined according to Ntatsi et al. (2017). The amount of K^+, P and Na^+ were assayed via an atomic absorption spectrophotometer, microplate spectrophotometer, and flame photometer, respectively.
Membrane permeability was monitored based on procedures described in Lutts et al. (1996).
Essential oil extraction:
The essential oil of peppermint was extracted from dried leaves by using hydro-distillation in Clevenger's apparatus. Chemical composition of peppermint essential oil (Figure. 1) was analyzed by gas chromatography/mass spectrometry (GC/MS) (Khalvandi et al, 2019).
Statistical analysis*:*
SAS (9.2) statistical program was used for statistical analysis and a mean between treatments was compared using an LSD (Least Significant Difference) test ($P <0.05$). Principal component analysis (PCA) and Heatmap was performed with the R language.

3. Results

Analysis of variance showed that salinity stress, P. indica, and AMF significantly affected all measured traits. According to the results, a significant two-way interaction was observed between salinity and inoculation treatment in essential oil, Na, membrane electrolyte leakage, Fv/Fm, ΦPSII, Fo, and stomatal conductance (Table 2 and 3); however, no significant Interaction was observed in other parameters. The main effects were significant for Fm, Fv, P, K, ETR, NPQ, Y(NPQ), Y(NO), chlorophyll a, and chlorophyll b (Table 2 and 3).

Colonization rate

In the microscopic investigation, not only chlamydospores and hyphae of *P. indica*, and vesicles, arbuscules, and intraradical hyphae of AMF were observed in the peppermint root cortex, but also a mycelial network of AMF and *P. indica* was spread around the roots (Figure. 2). The investigation into the AMF and *P. indica* and co-inoculation of them with plants showed 87%, 77%, and 89% colonization rate, respectively. Salt stress significantly reduced the ability of endophytic fungi to colonize with peppermint root. The decline was more noticeable when plants were inoculated with *P. indica* under medium level of salinity (6 dS $m^{-1)}$. Generally, the highest decline in the percentage of root colonization in both fungi (AMF and *P. indica*) was observed in the 9 dS m^{-1} level of salinity. The synergistic association of *P. indica* and AMF considerably increased the colonization ability of them with peppermint roots even under intense salt stress (Figure. 3).

Drought stress increases membrane electrolyte leakage (EL)

Membrane electrolyte leakage was increased under salinity stress by up to 70.4% (9 dSm^{-1}) (Figure. 4.a and Table S1), while microbial inoculation significantly alleviated oxidative damage to the membrane permeability even under severe salt stress. However, the difference between *P. indica* and AMF was not significant. The decrease in the percentage of EL was more noticeable when plants were co-inoculated by both *P. indica* and AMF. The results showed that co-inoculation was more effective than either single inoculation with *P. indica* or AMF for reducing membrane electrolyte leakage in the high level of salinity (9 dSm^{-1}).

Chlorophyll fluorescence parameters

Primary fluorescence (Fo), Maximum fluorescence (Fm), variable fluorescence (Fv)

It is known that salinity stress causes photosynthetic apparatus injury . Our data showed that salinity and microbial inoculation remarkably affected the Chlorophyll fluorescence; as salt stress led to a remarkable increase in Fo value, with maximum enhancement (29.6% and 28.9%, respectively compared to the control treatment) in plants which were grown under medium and high levels of salinity (Figure. 5a and Table S1). In addition, the increase in Fo was consistently accompanied by a decline in Fm and Fv values in salinity-treated leaves compared to the control (33.3%, and 48.81% reduction, respectively, when plants were subjected to the severe stress) (Figure. 6 and Table S2). As indicated in the figure. 5a, symbiotic relationship with *P. indica* and AMF and their co-inoculation significantly reduced Fo, while the same treatments significantly enhanced Fm and Fv. The highest Fm and Fv were observed in *P. indica* -inoculated plants (7.09%, and 24.33% higher, respectively compared to the control) (Figure. 6 and Table S3).

Maximum photochemical quantum yield of PSII (Fv/Fm) was reduced under salt stress

The Fv/Fm ratio considerably decreased when plants were subjected to salinity stress; the reduction was even greater when the higher salinity level (9 dSm^{-1}) was applied (Figure. 5a). As expected, the symbiotic relationship with endophytic fungi was associated with the increased Fv/Fm ratio under both normal and salinity conditions. This improvement was more noticeable when salinity reached 9 dS m^{-1}, as almost increased by 11.47 %, 9.83%, and 6.55%, with single and co-inoculation with *P. indic*a and AMF respectively compared to the control plants (Figure. 5a and Table S1).

Salt stress significantly reduced photochemical efficiency of photosystem II (ΦPSII) and ETR

In this experiment, ΦPSII and ETR values were sharply decreased under saline condition (Table S2), with maximum reduction (39.7% and 53.36% lower respectively, compared to the control) in plants grown under high salinity (9 dSm^{-1}) (Figure. 5 and 6). In general, endophytic fungi and especially their co-inoculation significantly

improved the ability of plants to respond to the negative effects of salinity, with the most obvious improvement of ΦPSII in 9 dSm^{-1}, (13.86% compared to control plants) (Figure. 5). ETR was also positively affected by endophytic fungi inoculation, as the highest ETR (24.11%) was observed in co-inoculated plants (Figure. 6 and Table S3)

Nonphotochemical chlorophyll quenching (NPQ), YNPQ and YNO were increased under salt stress

Salinity had significant effects on NPQ, YNPQ and YNO values in peppermint plants. The plants subjected to salt stress showed a remarkable increase in NPQ, YNPQ and YNO values in comparison to the control plants. The highest NPQ, YNPQ and YNO values (30.76%, 52.17% and 49.09%, respectively compared to controlled plants) was observed in plants which were grown under salt stress (9dsm^{-1}) (Figure. 6 and Table S2).

The results indicated that the fungi inoculation significantly reduced the NPQ, YNPQ and YNO values compared to controlled plants. The most reduction was observed in co-inoculated plants (Figure. 6 and Table S3).

Stomatal conductance (gs) is decreased under salt stress

The effect of saline condition on the stomatal conductance (gs) in the peppermint plants is shown in Figure. 5d. Increasing salinity led to a reduction in stomatal conductance. As indicated in Figure. 5d, gs reached to the lowest level under 9 dSm^{-1} of salinity (84.2% lower compared to the control). In contrast, fungi inoculation remarkably increased gs (53.87%, 47.91%, and 57% higher in *P. indica*, AMF and co-inoculation, respectively compared to un-inoculated plants grown under salt stress (9dsm^{-1})) (Figure. 5d and Table S1).

Salt stress reduces chlorophyll content

Salt stress led to a remarkable decrease in chlorophyll a and b compared to the control. The lowest contents of chlorophyll a and b, were observed in the plants treated with sever salinity (35.82% and 26.87%, respectively compared to the control plants) (Figure. 6b and Table S2). As it can be seen in Figure 6a and Table S3, fungi inoculation and especially co-inoculation significantly enhanced chlorophyll a and b contents (14.71% and 12.66%, respectively compared to controlled plants).

P, K, Na

The effect of saline condition on the minerals (P, K^+ and Na^+) concentrations in the peppermint plants is shown in Figure. 4c and 7. According to the results, Na content was dramatically increased in the peppermint leaves when plants were exposed to salt stress. Fungi inoculation ameliorated salinity negative effects, and the alleviation ratio (25.11% lower than control plant) was more considerable in co-inoculated plants under high (9 dSm^{-1}) level of salinity (Figure. 4c and Table S1). However, the opposite trend was observed for K^+ and P, as increasing in Na level was accompanied by a decline in P and K^+ content. This reduction was even greater when the higher salinity concentration (9 dSm^{-1}) was used (49.53% and 76.27% respectively, compared to the control) (Figure. 7a and 7b and Table S2). Our data shows that microbial inoculation positively affected the absorption of P and K in rhizosphere soil. The highest P content (43.58%) was observed in co-inoculated plants (Figure. 7d and Table S3).

Principal component of chlorophyll fluorescence parameters

Principal component analysis was conducted on the data of the salinity stress × 9 traits selected of leaf chlorophyll fluorescence parameters. The association between the traits were compared across the control, AMF, *P. indica* and co-inoculation treatments (Figure. 8). The score plot displayed four different groups, which were associated with four salinity samples, indicating a distinct separation of the chlorophyll fluorescence profiles under salinity conditions (Figure. 8). The PC1 mainly explained the separation of samples caused by 6 and 9 sm^{-1} of salinity (inoculation and non-inoculation), which was significantly positively correlated with YNPQ, YNO, Fo and, NPQ; whereas, PC2 mainly described non-stressed peppermint plants under low and mild salinity (inoculation and non-inoculation), it also showed the positive correlation with the Fm, Fv, Fv/Fm, ETR and, ΦPSII. Additionally, PCA findings, showed a tight positive association between Fm and Fv while they were associated negatively with Fo.

Essential oil content and composition

Salinity stress negatively affected essential oil content. Generally, salinity levels decreased essential oil content, and the lowest amount was recorded under 9 dSm^{-1}. Surprisingly, fungal symbiosis had a positive impact on essential oil content under all evaluated conditions. However, when the higher salinity concentration (9 dSm^{-1}) was used, co-inoculation was more effective in improving essential oil content compared to those of single inoculated plants (Figure. 4b and Table S1).

Essential oil composition was affected by *P. indica*, AMF, co-inoculation, salinity, and their interactions (Table 4 and Figure. 10). In all essential oil samples, the major constituents were Menthone, Menthol, 1,8-Cineole, and iso-menthone, respectively. As it can be seen in Table 4, the pattern of Menthone and Menthol increase differed in stress and non-stress conditions, indicating that microbial inoculation positively enhanced Menthone and Menthol content in both conditions. The highest Menthone content was recorded in the AMF-inoculated plant's essential oil, whereas the highest Menthol content as the second main component was observed in co-inoculated plants grown under salt stress (Table 4). A similar pattern was also observed for 1,8-Cineole, while the pattern for Menthofuran, another major component, was completely different, where salinity and Microbial inoculation reduced this content compared to the control plants (Table 4).

Piperitone content was increased under high salinity stress (9 dSm^{-1}) while β – Pinene and Terpinolene decreased compared with control samples under the same salinity level. Under both control and salinity conditions, fungi

inoculation enhanced α-Pinene, β – Pinene, 1,8-Cineole, Menthone, Menthol, neoisomenthol, Pulegone, α-Terpineol, Menthyl acetate, Carvacrol, methyl ether, β – Bourbonene, Caryophyllene, and germacrene D. In addition, higher levels of Menthone, Menthol and 1,8-Cineole were considerable in co-inoculated plant under salinity stress.

Heatmap and principal component of the essential oil constituents

As it can be seen in Figure. 9 and 10, Dim1 or PC1 accounted for 42.3% of the total variation and was mainly influenced by hydrocarbon monoterpenes (which is mainly due to the relative percentage variation of menthol), hydrocarbon sesquiterpenes, and alcohol monoterpenes. The PC1 mainly explained the separation of samples caused by endophytic fungi (salinity and non-salinity conditions), whereas, PC2 mainly explained salinity-treated and control peppermint plants (Figure. 9).

A Heatmap analysis was carried out in order to identify the significantly changed metabolites between inoculated and non-inoculated peppermint under salinity and non-salinity conditions (Figure. 11). Interestingly, salinity and non-salinity treatment (inoculated plants and non-inoculated plants) were separated from each other by two opposing groups (Figure. 11). Based on cluster analysis, two clusters of metabolites can be identified; all metabolites of the PC1, except α-Humulene, were classified in the first cluster, whereas the other group consisted of the PC2. However, linalool, Piperitone and Menthofuran were not included in either of the first two components. Controlled plants contained the higher iso-menthone, Terpinolene, and limonene together with Menthofuran and Viridiflorol which is related to oxygenated terpenes. It is possible to observe that co-inoculation treatment largely altered the relative percentage proportion of the essential oil compounds, with a high relative percentage of Menthol, Menthone, β - Pinene, 1,8-Cineole, Caryophyllene oxid, α-Terpineol, Menthyl acetate, β - Bourbonene, linalool, β – Farnesene, and sabinene. However, some metabolites showed higher percentages in control plants and co-inoculation had no significant effect on their production. The significantly changed metabolites in peppermint leaves under salinity stress were shown in Figure. 14.

4. Discussion

In the present study, the root colonization with *P. indica* and AMF were remarkably reduced by increasing salinity (6 to 9 dSm^{-1}). Similar observations were reported in other plants in which salinity inhibited the formation of the spores and arbuscules and growth of fungal hyphae (McMillen et al., 1998, Wu et al., 2010; khalvandi et al, 2019; Hadian-Deljou, et al, 2020).

It has been well documented that salinity induces excessive generation of ROS, the phenomenon that causes oxidative damage to thylakoid membrane components (disrupts the functional integrity of lipid membranes), and impairs electron transport function (Yang et al, 2021). In the current study, while salt stress decreased ETR, Fm, Fv/Fm ratio, and ΦPSII, the Fo and NPQ rates showed an increase under salinity conditions. Such alterations in fluorescence parameters can mainly be linked to the impairment of PSII function and inhibition of the electron transfer process; this may probably be due to 1: the membrane system damage in peppermint, which increases membrane electrolytic leakage (Figure. 4a), and 2: an increase in toxic ions such as $Na+$ (Figure. 4c).

These results provide a correlation (97%, $P < 0.05$) between the Fm and chlorophyll a. It should be mentioned that the decrease of Fm in response to salinity was along with a reduction in chlorophyll content (Figure. 6b). Fm reduction may be linked to the deactivation of chlorophyll protein compounds due to leaf chlorosis, or to alterations in any biochemical reactions (Gilmore and Björkman, 1995). Similar findings about increased Fo and decreased Fm were also reported on maize under salt stress (Sheng et al, 2008), low temperature stress (Zhu et al., 2010), and on winter wheat under drought stress (Lu and Zhang, 1998).

Increased Fo rate can indicate potential damages to PSII; damages such as D1 protein degradation, decreased thylakoid membrane integrity, damage to electron transport chain of photosystem II, reduction of plastoquinone electron receptors, lack of complete oxidation plastoquinone, and separation of light-harvesting protein complexes in chlorophyll a/b (Calatayud et al., 2006; Baker, 2008; Ashraf and Harris, 2013).

Fv/Fm represents the energy transfer efficiency from the light-harvesting complex of photosystem II to the photosystem II reaction center. Fv/Fm reduction under salinity stress can be due to limitation in the electron transport chain, which can destruct PSII reaction centers and reduce PSII maximum efficiency (Porcar-Castell et al., 2014; Giardi et al, 199, Shahabivand et al, 2017). In our experiment, the decrease in chlorophyll was accompanied by a decline in ΦPSII (Figure. 11), and Fv/Fm parameters under saline condition, which can be explained by a lower efficiency of energy transfer from antenna chlorophylls to the photosystem II reaction centers.

This result corroborates with those of previous studies reporting that salinity decreased Fv/Fm, ΦPSII and qp in durum wheat (Yaghoubi Khanghahi et al, 2020), Fv/Fm, ΦPSII, stomatal conductance and chlorophyll content in maize (Wang et al, 2020), and Chl a, Chl b, stomatal conductance and Fv/Fm in Canola (Athar et al, 2015). It has been well documented that reduction in the chlorophyll contents under salt stress reduces light reactions and electron transport in PS II from primary to secondary acceptor (Nilkens et al., 2010; Murchie and Lawson, 2013). Furthermore, we observed an inverse relationship between ΦPSII and NPQ value in peppermint ($r^2 = 0.81$; $p < 0.001$) (Figure. 12). The decrease in ΦPSII and the significant increase in NPQ in peppermint leaves under salinity stress indicates a decrease in photosynthetic process and carbon fixation capacity; this impairment decreases

utilization of electron transport products and induces greater thermal dissipation of light energy (Tavakkoli et al, 2010).
Our results showed that although the rate of colonization of *P. indica*, AMF and their co-inoculation symbiosis were negatively affected by high levels of salinity, the presence of these fungi in the roots of the plant caused a slight improvement in the function of the plant photosynthetic apparatus. According to the reports of some researchers, these changes can be due to the presence of symbiotic fungi in salinity stress conditions which can improve PSII function by increasing the transcription of chloroplast genes which are responsible for coding antenna proteins (involved in transferring stimulated energy) (Evelin et al, 2019).
The positive effects of plant-microbe interactions on PSII photochemical activity have been reported in many plants; for example, the interaction between AMF fungi and *P. indica* improved the efficiency of energy capture in PSII in pineapple plantlets (Moreira et al. 2015); *P. indica* increased photochemical efficiency of PSII, Fm, Fv/Fm and NPQ, and alleviated damages to the photosynthetic apparatus under salt stress in tomato plants (Ghorbani et al, 2018); symbiotic relationship with *P. indica* in wheat and sunflower, and *G. mosseae* in *Lolium perenne* L. protected photosystem II and enhanced the photosynthetic electron transport capacity of PSII, increased Fv/Fm and ETR values, and Chl-a and Chl-b amounts in Cd-contaminated soil (Zhang et al, 2018; Shahabivand et al, 2017; Shahabivand et al, 2012).
Endophytic fungi can reduce the toxic impact of salinity on the chloroplast and chlorophyll by stimulating antioxidant capacity and secondary metabolism related to ROS scavenger activity (such as PPO, DPPH-scavenging activity), by synthesizing phenolic compounds in peppermint, and last but not least, by alleviating damages to the cell membranes caused by salt stress (Zai et al, 2012; Khalvandi et al, 2019; Sun et al, 2012). Yun et al. (2018) observed a definite relationship between quantum efficiency of photosystem II, electron transport chain, and salt tolerance with the activity of antioxidant enzymes and plant hormone levels in *Zea mays* L. plants under salinity stress (Yun et al, 2018).
It has been well documented that there is a close link between reduction of the quantum efficiency of PSII and photoinhibition and depression of photosynthesis under environmental stress. The above results suggest that a low light harvesting efficiency due to reduced chlorophyll content is the first step of photosynthesis inhibition in response to environmental stresses. In this study, a direct correlation between an increasing chlorophyll content and a high ΦPSII in microbial colonization treatments at the presence of salinity can be attributed to stimulating the accumulation of chlorophyll by the microbes which ameliorated the negative effects of salinity on light-harvesting and electron transfer to plastoquinone.
The results of this study showed that severe salinity stress remarkably reduced P and K^+ uptake in non-inoculated plant because of the high rate of Na^+ absorption. Moreover, the inhibitory effect of ion toxicity, such as Na^+ and Cl^-, on the mineral absorption directly influences photosynthetic performance. Several reports suggest a direct correlation between nutrient imbalance and the simultaneous decline in PSII function in salt-stressed plants (Loudari et al, 2020; Qu et al, 2012); it may be due to reducing membrane permeability and increasing leakage of ions (Figure. 4a), and antagonistic relationship between excess uptake of Na^+ and mineral nutrient such as P and K^+ under severe salinity stress (Figure. 12) leading to an imbalance in cellular ion homeostasis (Yang et al, 2021). Nevertheless, in inoculated plant endophytic fungal symbiosis increased P and K+ content (Figure. 6a) and decreased Na+ uptake in peppermint leaves (Figure. 4c). It has been well established that endophytic fungi can keep the balance of sodium to potassium ratio through inducing the expression of genes which are involved in sodium excretion and potassium entry into the roots. Also, vesicles and arbuscules play a key role in nutrient transfer, and particularly in mobilization of phosphorus (Kumar, 2020). Endophytic fungi can release P from sources which are not accessible for plant roots and transforms P to the available forms, which are transported to host plants through fungal hyphae directly or taken up by roots (Wu et al. 2018). This can be attributed to the ability of endophytic fungi to increase the activity of phosphate transporters and also to stimulate phosphatase activities in the plant as well as in the rhizosphere soil (Wu et al, 2018).
Increased mineral nutrients absorption due to plant-microbe interactions influences photosynthesis performance by regulating gas exchange and photosynthesis pigments. There is a close relationship between the chlorophyll and gas interchange with PSII efficiency (Figure. 12). Positive effects of fungal symbiosis on the capacity of gas exchange are mainly through elevating the mineral absorption and improving water status. In agreement with the present results, Xu et al (2016) reported that the positive effect of AMF on Fv/Fm increasing in maize seedlings can be attributed to the absorption of mineral nutrients, activating mediated genes and sink stimulation. AMF inoculation also positively influenced chlorophyll biosynthesis in *Ligustrum vicaryi* by alleviating the detrimental effects of salinity on Mg^{2+} absorption and therefore improved Fv/Fm values (Qiu et al, 2020).
Furthermore, monoterpene emissions protect the photosynthetic apparatus and maintain PSII stability under stressful conditions through inducing photoprotective mechanisms. Besides, it maintains PSII stability through partitioning of the excess electrons into other sinks in the terpenoid biosynthetic pathway (Bertamini et al, 2020). Based on the results, there was a close correlation between the ETR and some essential oil components (Menthol, Menthone, and 1,8-cineole) (Figure. 13). Similar observations were reported in other plants. For example: it has been shown a tight link between monoterpene emission, increasing Fv/Fm ratio, and ETR in the *Vitis vinifera*

under heat stress (Bertamini et al, 2020); a positive correlation between the secondary metabolites and the quantum efficiency of PII in *Lactuca sativa* was reported (Ouzounis et al, 2015); also, a positive tight link between ETR and UPSII with monoterpene and sesquiterpene emissions in Q. coccifera and R. officinalis, respectively (Ormeño et al, 2009); photosynthetic electron transport rate and limonene emission in *Artemisia annua* (Rapparini et al, 2008); and finally, monoterpene emissions in Quercus species (Niinemets et al, 2002). Besides, the quantum yield of PSII (ΔF/Fm) in *Quercus ilex* L. was higher when plants were exposed to selected exogenous monoterpenes (Loreto et al 1998). According to the results, higher ETR values besides a higher monoterpene content in mycorrhizal plants can be attributed to a better physiological status, changing in the pattern of carbon partitioning, and accumulation and biosynthesis of terpenes. The possible mechanism is that endophytic fungus exerts anti-stress effects by inducing the expression of antioxidant biosynthetic genes (antioxidant potential) and activating antioxidant enzymes. The mechanism which alleviates the damages to chloroplast and chlorophyll, helps to maintain the relative stability of the organelle, and improves performance of ROS scavengers, such as CAT, PPO, and SOD activities.
It is believed that phosphate bonds can be a limiting factor in terpenoid biosynthesis. Naturally, monoterpenes emission is controlled by the availability of photosynthetic substrates. The biosynthesis of terpenoids in endophytic fungi treated plants can mainly be linked to their role in mineral nutrients uptake, especially phosphorus supply for NADPH and ATP (substrates of terpenoids biosynthesis), and increasing peltate glandular trichomes (Zolfaghari et al, 2013; Rai et al,2001; Sirrenberg et al,2007; Song, 2005; Loomis, and Corteau, 1972). Several reports have suggested a direct correlation between the synthesis of menthol, menthone, 1,8-cineole, citral, linalool and caryophyllene with antimicrobial and fungicide properties. The possible mechanism can be explained by increasing biosynthesis of these compounds in inoculated plants through activating defensive reactions (Coban, et al., 2016; Khalvandi et al, 2019). It has also been shown that reduction in CO_2 assimilation capacity under stress condition and defense response to microbial inoculation probably redirected resources to the mevalonic acid (MVA) (Gupta et al, 2018; Vickers et al, 2009).

5. Conclusion

Co-inoculation of mycorrhiza and *Piriformospora indica* fungi had a synergic effect on the enhancement of peppermint essential oil and its physiological characteristics. Fungal co-inoculation protected peppermint plants under salinity conditions. There are many reports about the synergistic effect of symbiotic fungi on the improvement of plant function under various environmental conditions; however, there are other research which did not support the synergistic effect and emphasized that separate application of endophyte fungi had more significant effect on the antioxidant activity and Fv / fm. (Shahabivand et al., 2012; Moreira et al., 2016; Ray and Walsalakumar, 2010). Thus, it needs further research in the future because applying microbial treatments for boosting plant secondary metabolite production could be a natural way as well as a sustainable approach in the promising herbal medicine industry.

Reference

Abdelshafy Mohamad, O. A., Ma, J. B., Liu, Y. H., Zhang, D., Hua, S., Bhute, S., ... & Li, L. (2020). Beneficial endophytic bacterial populations associated with medicinal plant Thymus vulgaris alleviate salt stress and confer resistance to Fusarium oxysporum. Frontiers in plant science, 11, 47.

Arnon, D. I. (1949). Copper enzymes in isolated chloroplasts. Polyphenol oxidase in Beta vulgaris. Plant physiology, 24(1), 1.

Aroca, R., Ruiz-Lozano, J.M., Zamarreño, Á.M., Paz, J.A., García-Mina, J.M., Pozo, M.J., López-Ráez, J.A., (2013). Arbuscular mycorrhizal symbiosis influences strigolactone production under salinity and alleviates salt stress in lettuce plants. J. Plant Physiol.

170, 47–55.

Ashraf, M., Harris, P.J.C., (2013). Photosynthesis under stressful environments: an overview. Photosynthetica 51, 163e190.

Ashraf, M., Shahzad, S.M., Imtiaz, M., Rizwan, M.S., (2018). Salinity effects on nitrogen metabolism in plants–focusing on the activities of nitrogen metabolizing enzymes: A review. J. Plant Nutr. 41, 1065–1081.

Athar, H. U. R., Zafar, Z. U., & Ashraf, M. (2015). Glycinebetaine improved photosynthesis in canola under salt stress: evaluation of chlorophyll fluorescence parameters as potential indicators. Journal of agronomy and crop science, 201(6), 428-442.

Baker, N.R., (2008). Chlorophyll fluorescence: a probe of photosynthesis in vivo. Annu. Rev. Plant Biol. 59, 89e113.

Baldi, A., Farkya, S., Jain, A., Gupta, N., Mehra, R., Datta, V., Srivastava, A.K., & Bisaria, V. S. (2010). Enhanced production of podophyllotoxins by co-culture of transformed Linum album cells with plant growth-promoting fungi. Pure and Applied Chemistry, 82(1), 227-241.

Bertamini, M., Faralli, M., Varotto, C., Grando, M. S., & Cappellin, L. (2020). Leaf Monoterpene Emission Limits Photosynthetic Downregulation Under Heat Stress in Field-Grown Grapevine.

Calatayud, A., Roca, D., Martínez, P.F., (2006). Spatial-temporal variations in rose leaves under water stress conditions studied by chlorophyll fluorescence imaging. Plant Physiol. Biochem. 44, 564e573.

Chen, J., Zhang, H., Zhang, X., & Tang, M. (2017). Arbuscular mycorrhizal symbiosis alleviates salt stress in black locust through improved photosynthesis, water status, and K+/Na+ homeostasis. Frontiers in plant science, 8, 1739.

Coban, O., Göktürk Baydar, N., (2016). Brassinosteroid effects on some physical and biochemical properties and secondary metabolite accumulation in peppermint (*Mentha piperita* L.) under salt stress. Ind Crops Prod. 86, 251-258.

Dolatabadi, H. K., Goltapeh, E. M., Moieni, A., & Varma, A. (2012). Evaluation of different densities of auxin and endophytic fungi (Piriformospora indica and Sebacina vermifera) on Mentha piperita and Thymus vulgaris growth. African Journal of Biotechnology, 11(7), 1644-1650.

Dolatabad, H. K., Goltapeh, E. M., Safari, M., & Golafaie, T. P. (2017). Potential effect of Piriformospora indica on plant growth and essential oil yield in Mentha piperita. Plant Pathol. Quarantine, 7, 96-104.

Evelin, H., Devi, T. S., Gupta, S., & Kapoor, R. (2019). Mitigation of salinity stress in plants by arbuscular mycorrhizal symbiosis: current understanding and new challenges. Frontiers in Plant Science, 10, 470.

Giardi, M. T., Cona, A., Geiken, B., Kučera, T., Masojidek, J., & Mattoo, A. K. (1996). Long-term drought stress induces structural and functional reorganization of photosystem II. Planta, 199(1), 118-125.

Gilmore AM, Björkman O. (1995). Temperature sensitive coupling and uncoupling of ATPase-mediated, nonradiative energy dissipation: Similarities between chloroplasts and leaves. Planta. 197:646-654.

Ghorbani, A., Razavi, S. M., Ghasemi Omran, V. O., & Pirdashti, H. (2018). Piriformospora indica inoculation alleviates the adverse effect of NaCl stress on growth, gas exchange and chlorophyll fluorescence in tomato (Solanum lycopersicum L.). Plant Biology, 20(4), 729-736.

Gupta, R., Singh, A., Srivastava, M., Singh, V., Gupta, M. M., & Pandey, R. (2017). Microbial modulation of bacoside A biosynthetic pathway and systemic defense mechanism in Bacopa monnieri under Meloidogyne incognita stress. *Scientific reports*, *7*(1), 1-11.

Hadian-Deljou, M., Esna-Ashari, M., & Mirzaie-asl, A. (2020). Alleviation of salt stress and expression of stress-responsive gene through the symbiosis of arbuscular mycorrhizal fungi with sour orange seedlings. *Scientia Horticulturae*, *268*, 109373.

Hasan, M., Ma, F., Prodhan, Z., Li, F., Shen, H., Chen, Y., & Wang, X. (2018). Molecular and physio-biochemical characterization of cotton species for assessing drought stress tolerance. International journal of molecular sciences, 19(9), 2636.

Haberstroh, S., Kreuzwieser, J., Lobo-do-Vale, R., Caldeira, M. C., Dubbert, M., & Werner, C. (2018). Terpenoid emissions of two Mediterranean woody species in response to drought stress. Frontiers in plant science, 9, 1071.

Hemming, D., 2013. Plant sciences reviews 2012. CABI. 280 pp. (online ISSN 1749-8848).

Kari Dolatabadi, H., Mohammadi Goltapeh, E., Jaimand, K., Rohani, N., Varma, A., (2011). Effects of *Piriformospora indica* and *Sebacina vermifera* on growth and yield of essential oil in fennel (*Foeniculum vulgare*) under greenhouse conditions. J. Basic Microbiol. 33–39.

Keramati, S., Pirdashti, H., Babaeizad, V., & Dehestani, A. (2016). Essential oil composition of sweet basil (*Ocimum basilicum* L.) in symbiotic relationship with Piriformospora indica and paclobutrazol application under salt stress. *Acta Biologica Hungarica*, *67*(4), 412-423.

Khalvandi, M. Amerian, M.R., Pirdashti, H., Keramati, S., Hosseini, J. (2019). Essential oil of peppermint in symbiotic relationship with *Piriformospora indica* and methyl jasmonate application under saline condition. Industrial crops and products 127, 195-202.

Khatri, K., & Rathore, M. S. (2019). Photosystem photochemistry, prompt and delayed fluorescence, photosynthetic responses and electron flow in tobacco under drought and salt stress. Photosynthetica, 57(1), 61-74.

Kromdijk, J., Głowacka, K., Leonelli, L., Gabilly, S.T., Iwai, M., Niyogi, K.K., Long, S.P. (2016). Improving photosynthesis and crop productivity by accelerating recovery from photoprotection. Science 354, 857–861.

Kumar, G. (2020). Unit 7 Topic: Arbuscular Mycorrhizal Colonization In Plant Roots.

Li, D., Liu, H., Qiao, Y., Wang, Y., Cai, Z., Dong, B., Shi, C., Liu, Y., Li, X., and Liu, M. (2013). Effects of elevated CO2 on the growth, seed yield, and water use efficiency of soybean (*Glycine max* (L.) Merr.) under drought stress. Agricultural Water Management, 129, 105-112.

Li, Y., Song, H., Zhou, L., Xu, Z., & Zhou, G. (2019). Tracking chlorophyll fluorescence as an indicator of drought and rewatering across the entire leaf lifespan in a maize field. Agricultural Water Management, 211, 190-201.

Loomis, W.D., Corteau, R., 1972. Essential oil biosynthesis. Recent Adv Phytochem. 6, 147-185.

Loreto, F., Förster, A., Dürr, M., Csiky, O., & Seufert, G. (2002). On the monoterpene emission under heat stress and on the increased thermotolerance of leaves of Quercus ilex L. fumigated with selected monoterpenes. Plant, Cell & Environment, 21(1), 101-107.

Loudari, A., Benadis, C., Naciri, R., Soulaimani, A., Zeroual, Y., Gharous, M. E., Kalaji, H M., & Oukarroum, A. (2020). Salt stress affects mineral nutrition in shoots and roots and chlorophyll a fluorescence of tomato plants grown in hydroponic culture. Journal of Plant Interactions, 15(1), 398-405.

Lu, C., & Zhang, J. (1998). Effects of water stress on photosynthesis, chlorophyll fluorescence and photoinhibition in wheat plants. Functional Plant Biology, 25(8), 883-892.

Lutts, S., Kinet, J. M., & Bouharmont, J. (1996). NaCl-induced senescence in leaves of rice (Oryza sativa L.) cultivars differing in salinity resistance. Annals of botany, 78(3), 389-398.

Mansotra, P., Sharma, P., & Sharma, S. (2015). Bio augmentation of *Mesorhizobium cicer*, *Pseudomonas spp*. and *Piriformospora indica* for sustainable chickpea production. Physiology and molecular biology of plants, 21(3), 385-393.

McMillen, B.G., Juniper, S., Abbott, L.K., (1998). Inhibition of hyphal growth of a Vesicular arbuscular mycorrhizal fungus in soil containing sodium chloride limits the spread of infection from spores. Soil Biol. Biochem. 30, 1639–1646.

Miguel, M.G., (2011). Anthocyanins: Antioxidant and/or anti -inflammatory activities. J Appl Pharm Sci. 6, 7-15.

Moghaddama, M., Pourbaige, M., Kourosh Tabar, H., Farhadid, N., Ahmadi Hosseinie, S.M., 2013. Composition and Antifungal Activity of Peppermint (*Mentha piperita*) Essential Oil from Iran. J. Essent. Oil-Bear. Plants. 16, 506-512.

Monetti, E., Kadono, T., Tran, D., Azzarello, E., Arbelet-Bonnin, D., Biligui, B., Briand, J., Kawano, T., Mancuso, S., Bouteau, F., (2014). Deciphering early events involved in hyperosmotic stress-induced programmed cell death in tobacco BY-2 cells. J. Exp.

Bot. 65, 1361–1375.

Moreira, B. C., Junior, P. P., Jordao, T. C., da Silva, M. D. C. S., Stürmer, S. L., Salomão, L. C. C., ... & Kasuya, M. C. M. (2016). Effect of inoculation of symbiotic fungi on the growth and antioxidant enzymes' activities in the presence of Fusarium subglutinans f. sp. ananas in pineapple plantlets. Acta Physiologiae Plantarum, 38(10), 1-14.

Murchie, E.H., Lawson, T. (2013). Chlorophyll fluorescence analysis: a guide to good practice and understanding some new applications. J. Exp. Bot. 64, 3983–3998.

Niinemets, Ü., Hauff, K., Bertin, N., Tenhunen, J. D., Steinbrecher, R., & Seufert, G. (2002). Monoterpene emissions in relation to foliar photosynthetic and structural variables in Mediterranean evergreen Quercus species. New Phytologist, 153(2), 243-256.

Nilkens, M., Kress, E., Lambrev, P., Miloslavina, Y., Müller, M., Holzwarth, A.R., Jahns, P. (2010). Identification of a slowly inducible zeaxanthin-dependent component of nonphotochemical quenching of chlorophyll fluorescence generated under steady-state conditions in Arabidopsis.

Norris, I.R., Read, D.J., Varma, A.K., 1992. Methods in Microbiology. Techniques for Study of Mycorrhiza. Academic Press, London. 450 pp.

Ntatsi, G., Aliferis, K.A., Rouphael, Y., Napolitano, F., Makris, K., Kalala, G., Katopodis, G., Savvas, D., 2017. Salinity source alters mineral composition and metabolism of *Cichorium spinosum*. Environ. Exp. Bot. 141, 113–123.

Ormeño, E., Olivier, R., Mévy, J. P., Baldy, V., & Fernandez, C. (2009). Compost may affect volatile and semi-volatile plant emissions through nitrogen supply and chlorophyll fluorescence. Chemosphere, 77(1), 94-104.

Ouzounis, T., Razi Parjikolaei, B., Fretté, X., Rosenqvist, E., & Ottosen, C. O. (2015). Predawn and high intensity application of supplemental blue light decreases the quantum yield of PSII and enhances the amount of phenolic acids, flavonoids, and pigments in Lactuca sativa. Frontiers in plant science, 6, 19.

Peñuelas, J., & Llusià, J. (2002). Linking photorespiration, monoterpenes and thermotolerance in Quercus. New Phytologist, 155(2), 227-237.

Porcar-Castell, A., Tyystj arvi, E., Atherton, J., Van Der Tol, C., Flexas, J., Pfündel, E.E., € Moreno, J., Frankenberg, C., Berry, J.A. (2014). Linking chlorophyll a fluorescence to photosynthesis for remote sensing applications: mechanisms and challenges. J. Exp. Bot. 65, 4065e4095.

Qiu, Y. J., Zhang, N. L., Zhang, L. L., Zhang, X. L., Wu, A. P., Huang, J. Y & Wang, Y. H. (2020). Mediation of arbuscular mycorrhizal fungi on growth and biochemical parameters of Ligustrum vicaryi in response to salinity. Physiological and Molecular Plant Pathology, 112, 101522.

Qu, C., Liu, C., Gong, X., Li, C., Hong, M., Wang, L., & Hong, F. (2012). Impairment of maize seedling photosynthesis caused by a combination of potassium deficiency and salt stress. Environmental and Experimental Botany, 75, 134-141

Radić, S., Peharec Štefanić, P., Lepeduš, H., Roje, V., Pevalek-Kozlina, B., (2013). Salt tolerance of Centaurea ragusina L. is associated with efficient osmotic adjustment and increased antioxidative capacity. Environ. Exp. Bot. 87, 39–48.

Rai, M., Acharya, D., Singh, A., Varma, A., (2001). Positive growth responses of the medicinal plants Spilanthes calva and Withania somnifera to inoculation by Piriformospora indica in a field trial. Mycorrhiza. 11, 123-128.

Rapparini, F., Llusià, J., & Peñuelas, J. (2008). Effect of arbuscular mycorrhizal (AM) colonization on terpene emission and content of Artemisia annua L. Plant Biology, 10(1), 108-122.

Ray, J. G., & Valsalakumar, N. (2010). Arbuscular mycorrhizal fungi and Piriformospora indica individually and in combination with Rhizobium on green gram. Journal of plant nutrition, 33(2), 285-298.

Shahabivand, S., Parvaneh, A., & Aliloo, A. A. (2017). Root endophytic fungus Piriformospora indica affected growth, cadmium partitioning and chlorophyll fluorescence of sunflower under cadmium toxicity. Ecotoxicology and environmental safety, 145, 496-502.

Sheng, M., Tang, M., Chen, H., Yang, B., Zhang, F., & Huang, Y. (2008). Influence of arbuscular mycorrhizae on photosynthesis and water status of maize plants under salt stress. Mycorrhiza, 18(6-7), 287-296.

Shahabivand, S., Maivan, H. Z., Goltapeh, E. M., Sharifi, M., & Aliloo, A. A. (2012). The effects of root endophyte and arbuscular mycorrhizal fungi on growth and cadmium accumulation in wheat under cadmium toxicity. Plant Physiology and Biochemistry, 60, 53-58.

Sirrenberg, A., Gobel, C., Grond, S., Czempinski, N., Ratzinger, A., Karlovsky, P., Santos, P., Feussner I., Pawlowski, K., (2007). *Piriformospora indica* affects plant growth by auxin production. Physiol. Plant. 131, 581-589

Su, Z. Z., Wang, T., Shrivastava, N., Chen, Y. Y., Liu, X., Sun, C., Yin, Y., Gao, Q.K. & Lou, B. G. (2017). *Piriformospora indica* promotes growth, seed yield and quality of *Brassica napus* L. Microbiological research, 199, 29-39.

Sun, C., Johnson, J. M., Cai, D., Sherameti, I., Oelmüller, R., & Lou, B. (2010). *Piriformospora indica* confers drought tolerance in Chinese cabbage leaves by stimulating antioxidant enzymes, the expression of drought-related genes and the plastid-localized CAS protein. *Journal of plant physiology*, *167*(12), 1009-1017.

Sun, Z., Wang, H., Wang, J., Zhou, L., Yang, P., (2014). Chemical composition and anti-inflammatory, cytotoxic and antioxidant activities of essential oil from Leaves of *Mentha piperita* grown in china. PLoS One. 12, e114767.

Tarraf, W., Ruta, C., Cillis, F.D., Tagarelli, A., Tedone, L., Mastro, G.D., (2015). Effects of mycorrhiza on growth and essential oil production in selected aromatic plants. J.

Tavakkoli, E., Rengasamy, P., & McDonald, G. K. (2010). High concentrations of Na+ and Cl–ions in soil solution have simultaneous detrimental effects on growth of faba bean under salinity stress. Journal of Experimental Botany, 61(15), 4449-4459.

Valifard, M., Mohsenzadeh, S., Kholdebarin, B., Rowshan, V., Niazi, A., & Moghadam, A. (2019). Effect of salt stress on terpenoid biosynthesis in Salvia mirzayanii: From gene to metabolite. The Journal of Horticultural Science and Biotechnology, 94(3), 389-399.

Varma, A., Verma, S., Sudha Sahay, N., Bu¨tehorn, B., Franken, P., 1999. Piriformospora indica, a Cultivable Plant-Growth- Promoting Root Endophyte. Appl. Environ. Microbiol. 65, 2741–2744.

Vickers, C. E., Gershenzon, J., Lerdau, M. T., & Loreto, F. (2009). A unified mechanism of action for volatile isoprenoids in plant abiotic stress. *Nature chemical biology*, *5*(5), 283.

Vierheilig, H., Coughlan, A.P., Wyss, U., Piche, Y., 1998. Ink and vinegar, a simple staining technique for arbuscular-mycorrhizal fungi. Appl. Environ. Microbiol. 12, 5004-5007.

Tsai, M.L., Wu, C.T., Lin, T.F., Lin, W.C., Huang, Y.C., Yang, C.H., (2013). Chemical composition and biological properties of essential oils of two mint species. Trop. J. Pharm. Res. 12, 577–582.

Wang, H., Liang, L., Liu, B., Huang, D., Liu, S., Liu, R., ... & Chen, Y. (2020). Arbuscular Mycorrhizas Regulate Photosynthetic Capacity and Antioxidant Defense Systems to Mediate Salt Tolerance in Maize. Plants, 9(11), 1430.

Wu, Q.S., Zou, Y.N., He, X.H., (2010). Contributions of arbuscular mycorrhizal fungi to growth, photosynthesis, root morphology and ionic balance of citrus seedlings under salt stress. Acta Physiol. Plant. 32, 297–304. https://doi.org/10.1007/s11738-009-0407-z.

Wu, M., Wei, Q., Xu, L., Li, H., Oelmüller, R., & Zhang, W. (2018). *Piriformospora indica* enhances phosphorus absorption by stimulating acid phosphatase activities and organic acid accumulation in *Brassica napus*. *Plant and Soil*, *432*(1-2), 333-344.

Xu, H., Lu, Y., & Zhu, X. (2016). Effects of arbuscular mycorrhiza on osmotic adjustment and photosynthetic physiology of maize seedlings in black soils region of northeast China. Brazilian Archives of Biology and Technology, 59.

Yaghoubi Khanghahi, M., Strafella, S., & Crecchio, C. (2020). Changes in photo-protective energy dissipation of photosystem II in response to beneficial bacteria consortium in durum wheat under drought and salinity stresses. Applied Sciences, 10(15), 5031.

Yang, M., Sun, X., Zhu, J., Liu, Y., Chen, N., & Zhao, G. Physiological and proteomics insights into salt tolerance of two Jerusalem artichoke cultivars. Journal of Plant Biochemistry and Biotechnology, 1-10.

Yun, P., Xu, L., Wang, S.S., Shabala, L., Shabala, S., Zhang, W.Y. (2018). *Piriformospora indica* improves salinity stress tolerance in *Zea mays* L. plants by regulating Na+ and K+ loading in root and allocating K+ in shoot. Plant Growth Regul. 1–9. https://doi.org/10.1007/s10725-018-0431-3.

Zai, X. M., Zhu, S. N., Qin, P., Wang, X. Y., Che, L., & Luo, F. X. (2012). Effect of Glomus mosseae on chlorophyll content, chlorophyll fluorescence parameters, and chloroplast ultrastructure of beach plum (*Prunus maritima*) under NaCl stress. *Photosynthetica*, *50*(3), 323-328.

Zolfaghari, M., Nazeri, V., Sefidkon, F., Rejali, F., (2013). Effects of arbuscular mycorrhizal fungi on plant growth and essential oil content and composition of *Ocimum basilicum* L. J Plant Physiol. 3, 643-650.

Zhu, X. C., Song, F. B., & Xu, H. W. (2010). Arbuscular mycorrhizae improve low temperature stress in maize via alterations in host water status and photosynthesis. Plant and Soil, 331(1-2), 129-137.

Table 1. Chemical analysis of Caspian Sea water

parameter	unit	amount
EC	dS m^{-1}	15
pH	-	8.2
sodium	ppm	376.48
potassium	ppm	77.11
calcium	ppm	17
magnesium	ppm	54.5

(Khalvandi et al, 2019)

Table 2. Variance analysis of salinity and *P. indica* and AMF effects on measured chlorophyll fluorescence parameters of peppermint

S.O.V	df	fo	YII	Fv/fm	fm	fv	Y(NPQ)	Y(NO)	NPQ	ETR
Replication	2	0.0017	0.00337	0.00116	0.7396	0.7337	0.0004	0.0011	0.0045	14.7708
Salinity (S)	3	0.6930**	0.14945**	0.09022**	13.4999**	20.2384**	0.0446**	0.0456**	0.3145**	300.055**
Fungi (F)	3	0.0412**	0.01461**	0.00362**	0.4269**	0.6231**	0.0054**	0.0031**	0.0153**	41.50**
S × F Interaction	9	0.0047**	0.00097**	0.00049**	0.1005ns	0.0988ns	0.0015 ns	0.0005ns	0.0042ns	3.7037 ns
Error	30	0.0015	0.00034	0.00013	0.0772	0.0736	0.00072	0.00026	0.0027	3.304
CV%		3.04	3.26	1.52	4.83	6.11	15.44	8.3	5.42	3.304

ns = no significant, * significant (P < 0.05), ** significant (P < 0.01)

Table 3. Variance analysis of salinity and *P. indica* and AMF effects on measured physiological and biochemical parameters of peppermint

S.O.V	df	Chlorophyll a (μg/ml)	Chlorophyll b (μg/ml)	P (meq/gdw^{-1})	K^+ (meq/gdw^{-1})	Na^+ (meq/gdw^{-1})	Essential oil (%)	Stomatal conductance (mmol (H_2O) m^{-2} s^{-1})	Membrane electrolyte leakage %	colonization rate (%)
Replication	2	0.3765	0.50701	0.00255	0.02985	0.0043	0.00316	0.0593	2.9203**	11.583
Salinity (S)	3	13.9547**	5.57805**	0.12515**	3.99761**	2.1872**	6.81303**	655.0106**	4835.1701**	2267.629**
Fungi (F)	3	1.7688**	1.00595**	0.07111**	0.15185**	0.0690**	0.82588**	28.2901**	133.2135**	424.750**
S × F Interaction	9	0.0399ns	0.07208ns	0.00281ns	0.02509ns	0.0145*	0.04237**	0.4364*	13.5865**	13.824*
Error	30	0.08101	0.1196	0.00227	0.02054	0.0061	0.0068	0.1701	6.0838	5.2500
CV%		5.03	6.95	14.21	13.2	14.12	4.59	3.34	6.44	3.25

ns = no significant, * significant ($P < 0.05$), ** significant ($P < 0.01$)

Table 4. Effect of microbial inoculation on *Mentha piperita* essential oils composition under salinity stress.

No	Compound	Concentration (%)							
		Salinity* P. indica *AMF	Salinity *AMF	Salinity * P. indica	Salinit y	P. indica *AMF	P. indica	AMF	control
1	limonene	2.4	1.93	1.42	3.5	2.63	2.89	2.73	5.36
2	Carvacrol, methyl ether	1.38	1.77	2.08	1.23	2.01	2.21	1.9	1.38
3	Menthyl acetate	1.21	1.13	1.22	1.07	1.12	1.16	1.15	1.08
4	α-Pinene	1.04	1.07	1.09	1.03	1.05	0.93	1.02	0.85
5	sabinene	0.92	0.88	0.75	0.72	0.82	0.84	0.82	0.86
6	β - Pinene	0.92	0.51	0.61	0.43	0.61	0.6	0.63	0.52
7	β - Bourbonene	0.72	0.65	0.78	0.41	0.43	0.38	0.54	0.32
8	Terpinolene	0.43	0.14	0.35	0.31	0.49	0.48	0.53	0.81
9	α- Terpinene	0.28	0.27	0.23	0.23	0.41	0.48	0.42	0.64
10	cis-Ocimene	0.14	0.29	0.6	0.33	0.52	0.62	0.54	0.64
11	Piperitone	0.1	0.31	0.13	0.29	0.23	0.13	0.21	0.28
12	Menthol	19.87	19.37	18.03	15.39	18.12	18.89	17.3	14.86
13	neoisomenthol	5.41	5.27	6.18	4.46	5.37	6.09	5.35	5.07
14	Trans sabinene hydrate	0.92	0.61	0.98	0.68	0.87	0.81	0.8	0.85
15	1,8-Cineole	10.66	9.62	9.23	9.01	9.14	9.04	9.12	8.87
16	Menthofuran	5.22	6.18	5.07	5.87	5.63	5.61	5.48	7.33
17	α-Terpineol	0.87	0.52	0.85	0.58	0.75	0.63	0.72	0.61
18	Linalool	0.68	0.54	0.26	0.56	0.5	0.49	0.47	0.52
19	Menthone	34.52	31.27	32.98	31.04	34.75	34.95	35.2	29.41
20	iso-menthone	4.9	5.74	4.57	6.15	6.12	5.95	5.97	8.34
21	Pulegone	0.23	0.33	0.34	0.24	0.27	0.3	0.26	0.15
22	Caryophyllene	0.81	0.36	0.76	0.45	0.75	0.73	0.75	0.68
23	β - Farnesene	0.37	0.33	0.35	0.32	0.25	0.28	0.27	0.31
24	germacrene D	0.17	0.21	0.21	0.17	0.2	0.25	0.22	0.15
25	Caryophyllene oxid	0.19	0.13	0.14	0.11	0.11	0.12	0.1	0.06
26	α-Humulene	0.15	0.11	0.14	0.18	0.15	0.27	0.1	0.16
27	Viridiflorol	0.15	0.13	0.12	0.11	0.18	0.31	0.21	0.43

Salinity (9dsm^{-1}); *P. indica*; AMF – Mycorrhizae treatment; P. indica *AMF – *P. indica* and Mycorrhizae treatment.

Fig. 1. *Mentha piperita* essential oil chromatogram

Fig. 2. *Piriformospora indica* interacellular chlamydospores in cortex (a), *P. indica* spore and hyphae (around the roots) (b) and interadical hyphae, exteradical hyphae, vesicles and arbuscules formation of AMF (c and d) were formed in the *Mentha piperita* roots. The root-pieces were examined under light microscope at the magnification of 10–40 X.

Fig. 3. Effect of salinity on root colonization (*P. indica*, AMF and co-inoculation) in peppermint. In each figure, means with the same letter are significantly different according to LSD test at $P < 0.05$.

Fig. 4. Effect of salinity on Membrane electrolyte leakage (a), Essence (b) and Na (c) in inoculated and non-inoculated peppermint with (*P. indica* AMF and co-inoculation)

Fig. 5. Effect of salinity on Fo (a), ΦPSII (b), Fv/Fm (c) and Stomatal conductance (d) in inoculated and non-inoculated peppermint with (*P. indica*, AMF and co-inoculation)

Fig 6. Changes in various physiological traits in inoculated and non-inoculated peppermint with (*P. indica*, AMF and co-inoculation) (a), and caused by salinity stress in peppermint plant (b) *P. indica*; AMF – Mycorrhizae treatment; AMF* *P. indica* –Mycorrhizae and *P. indica* treatment.

Fig 7. Box Plots of P and K in peppermint under salinity stress (a) and in inoculated and non-inoculated peppermint with (*P. indica*, AMF and co-inoculation) (b). *P. indica*; AMF – Mycorrhizae treatment; AMF* *P. indica* –Mycorrhizae and *P. indica* treatment.

Fig. 8. Principal component analysis (PCA) (a) and 2D score plot (b) of chlorophyll fluorescence parameters in peppermint leaves. The score plot for the four-salinity levels; 0, 3, 6 and 9 dsm^{-1} were indicated in blue, orange, green and red, respectively. Abbreviations: *P. indica*; AMF – Mycorrhizae treatment; *P. indica**AMF – *P. indica* and Mycorrhizae treatment.

Fig. 9. Principal component analysis (PCA) of metabolites in peppermint leaves. AMF – Mycorrhizae treatment.

Fig 10. Changes in terpenoid content in inoculated and non-inoculated peppermint with (*P. indica*, AMF and co-inoculation) under salinity stress in peppermint plant. S – Salinity; *P. indica*; AMF – Mycorrhizae treatment; AMF* P. indica –Mycorrhizae and *P. indica* treatment.

Fig. 11. Heatmap pattern of the essential oil constituents from peppermint. AMF – Mycorrhizae treatment; *P. indica* *AMF – *P. indica* and Mycorrhizae treatment.

Fig 12. Relationship between some physiological parameters in inoculated and non-inoculated peppermint with (*P. indica*, AMF and co-inoculation) under salinity stress in peppermint plant. AMF – Mycorrhizae treatment; AMF* *P. indica* –Mycorrhizae and *P. indica* treatment.

Fig 13. Relationship between some metabolites in inoculated and non-inoculated peppermint with (*P. indica*, AMF and co-inoculation) under 9 dsm^{-1} salinity stress in peppermint plant. The salinity and non-salinity condition were indicated in orange and green, respectively.

Fig 14. Leaf metabolites involved in the terpenoied pathways in peppermint plant under salinity stress. The relative abundance of metabolites in inoculated and non-inoculated peppermint with (*P. indica* and AMF) under salt stress was shown as box plots. Regulated metabolites under salinity and non-salinity condition were indicated in orange and green, respectively.

Figure 2

Click here to access/download;Figure;2a.tiff

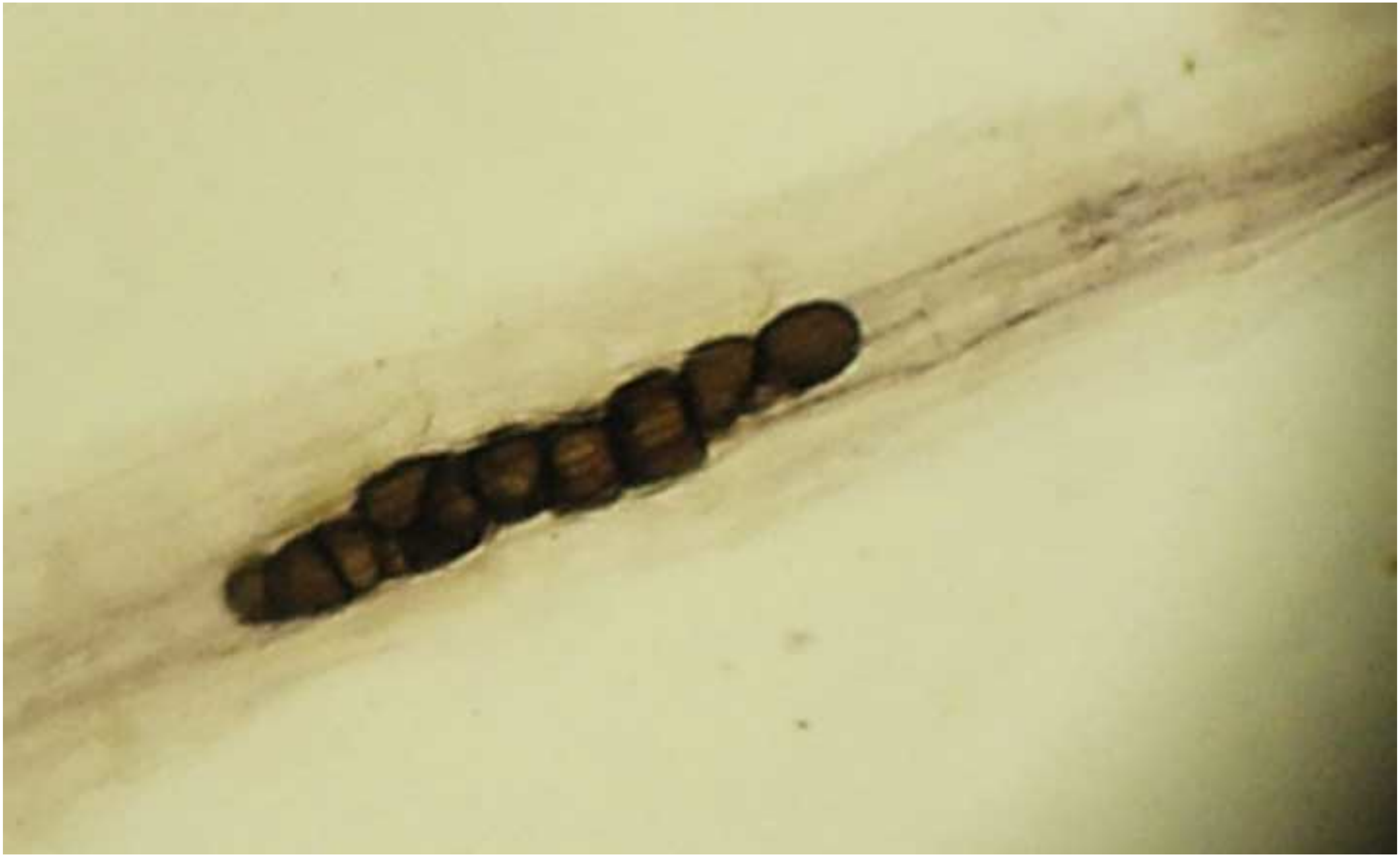

Figure 2

Click here to access/download;Figure;2b.tiff

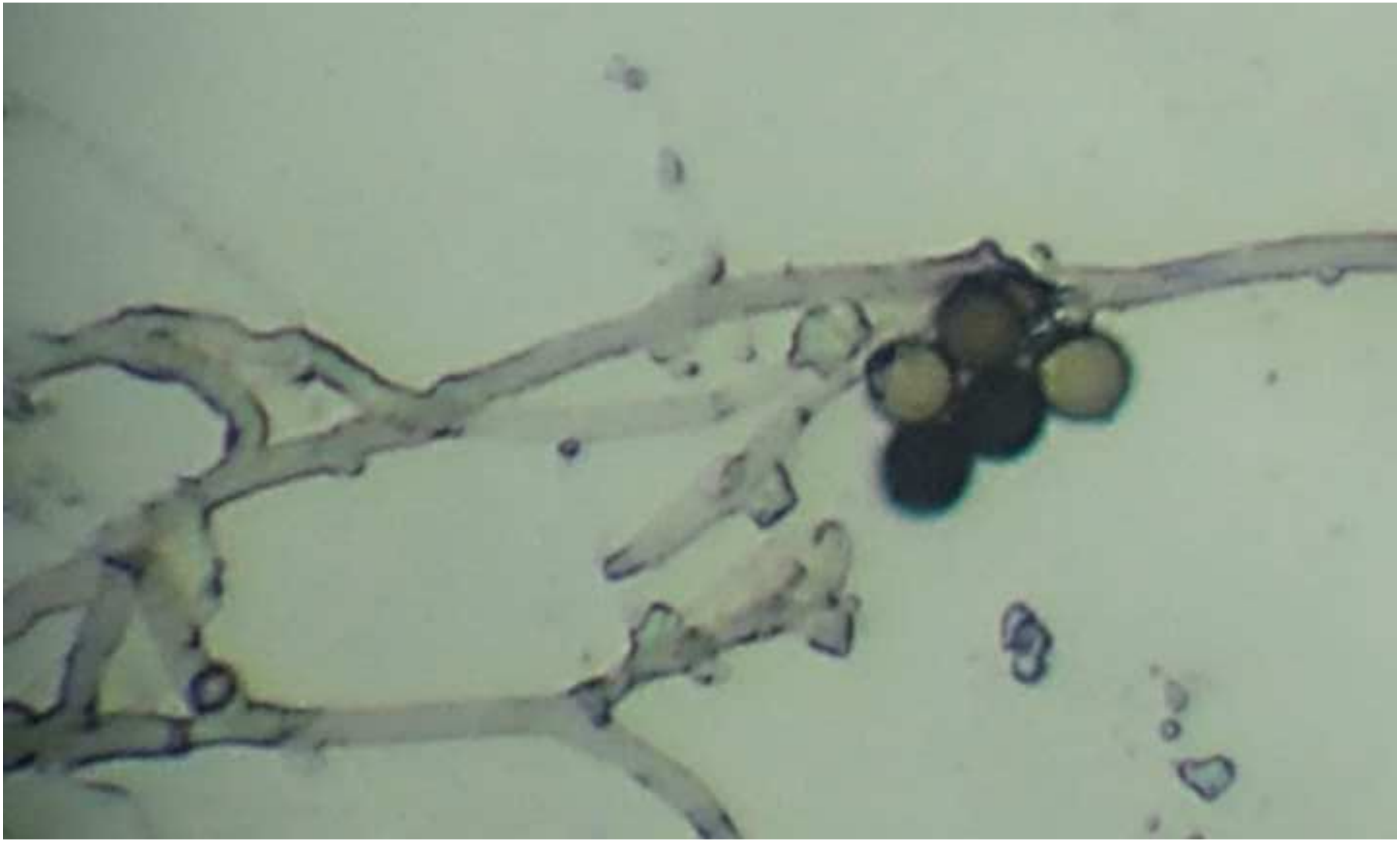

Figure 2

Click here to access/download;Figure;2c.tiff

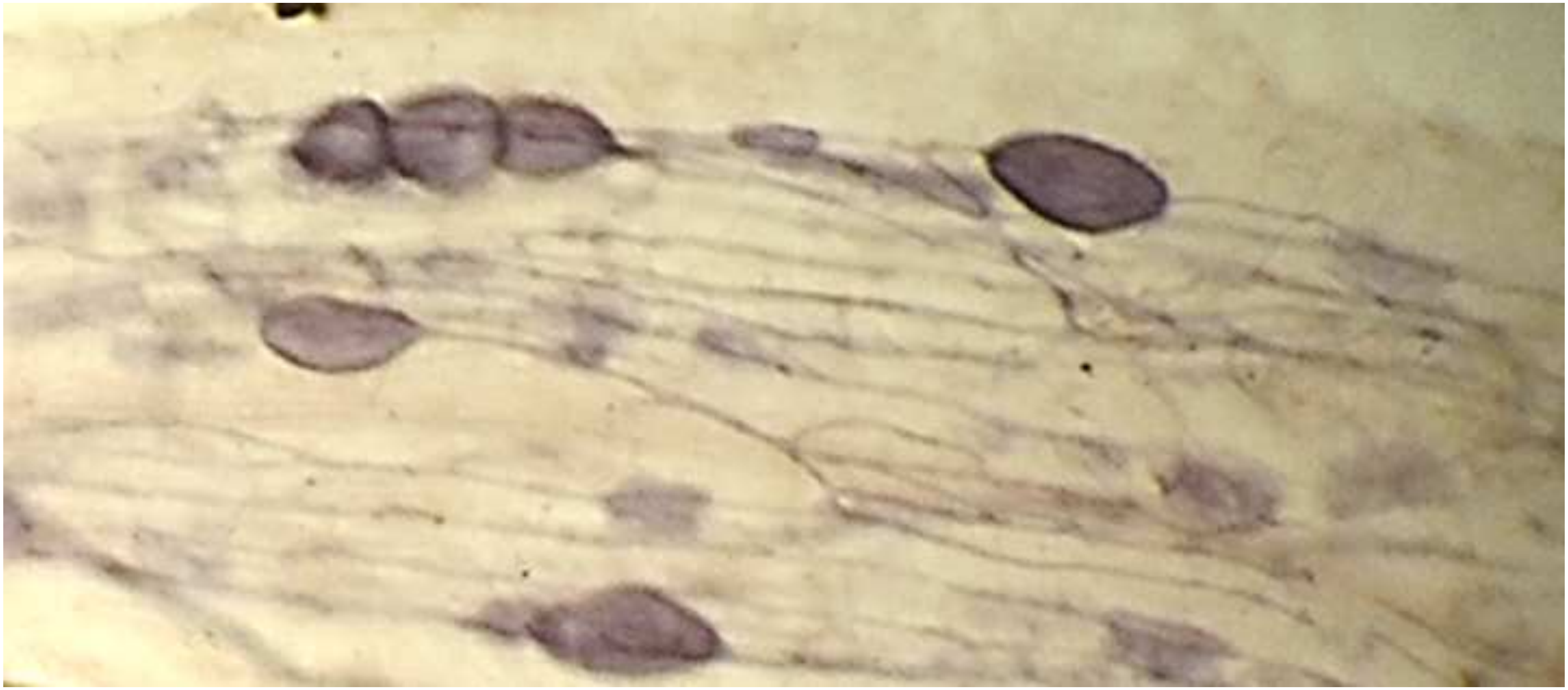

Figure 2

Click here to access/download;Figure;2d.tiff

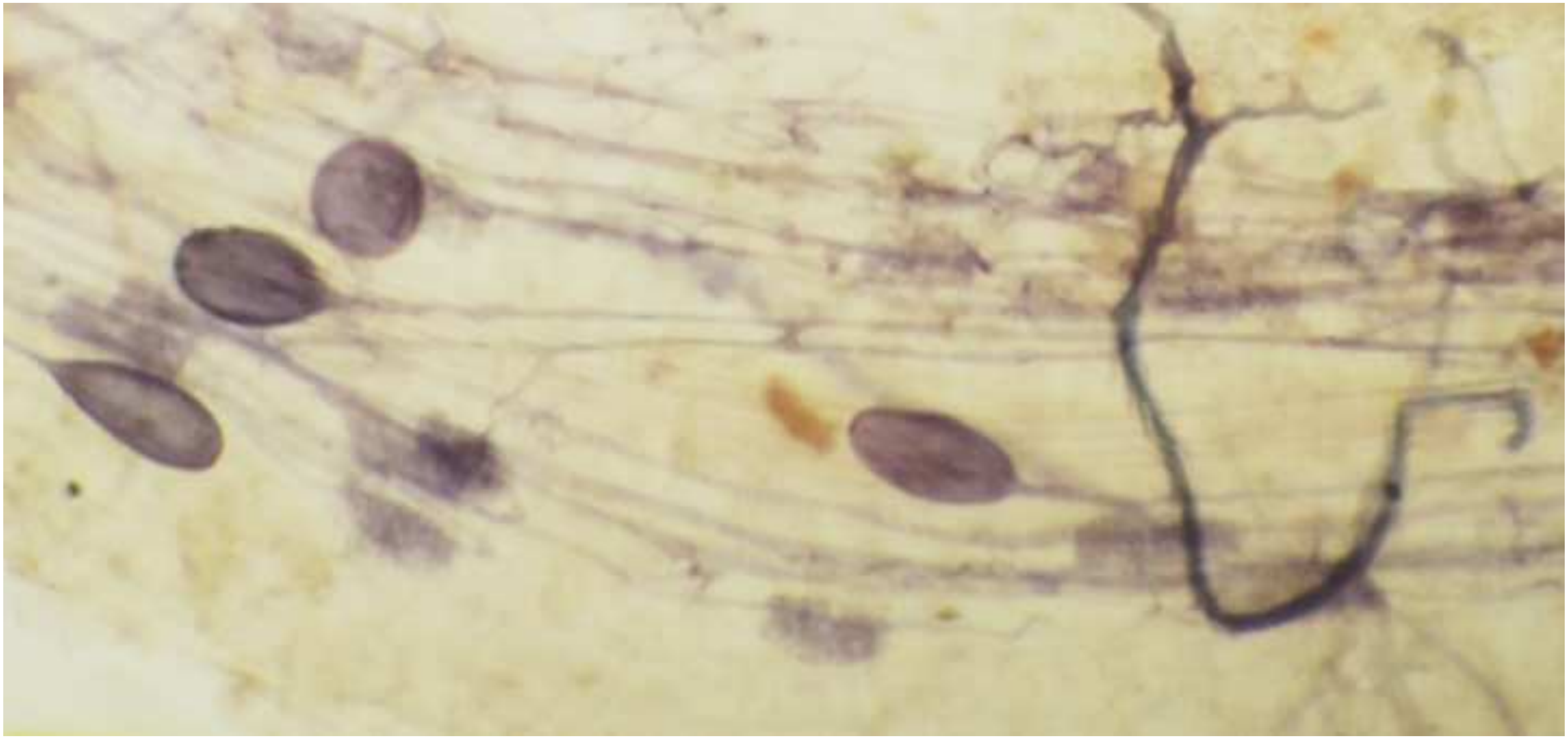

Click here to access/download;Figure;6a.tif

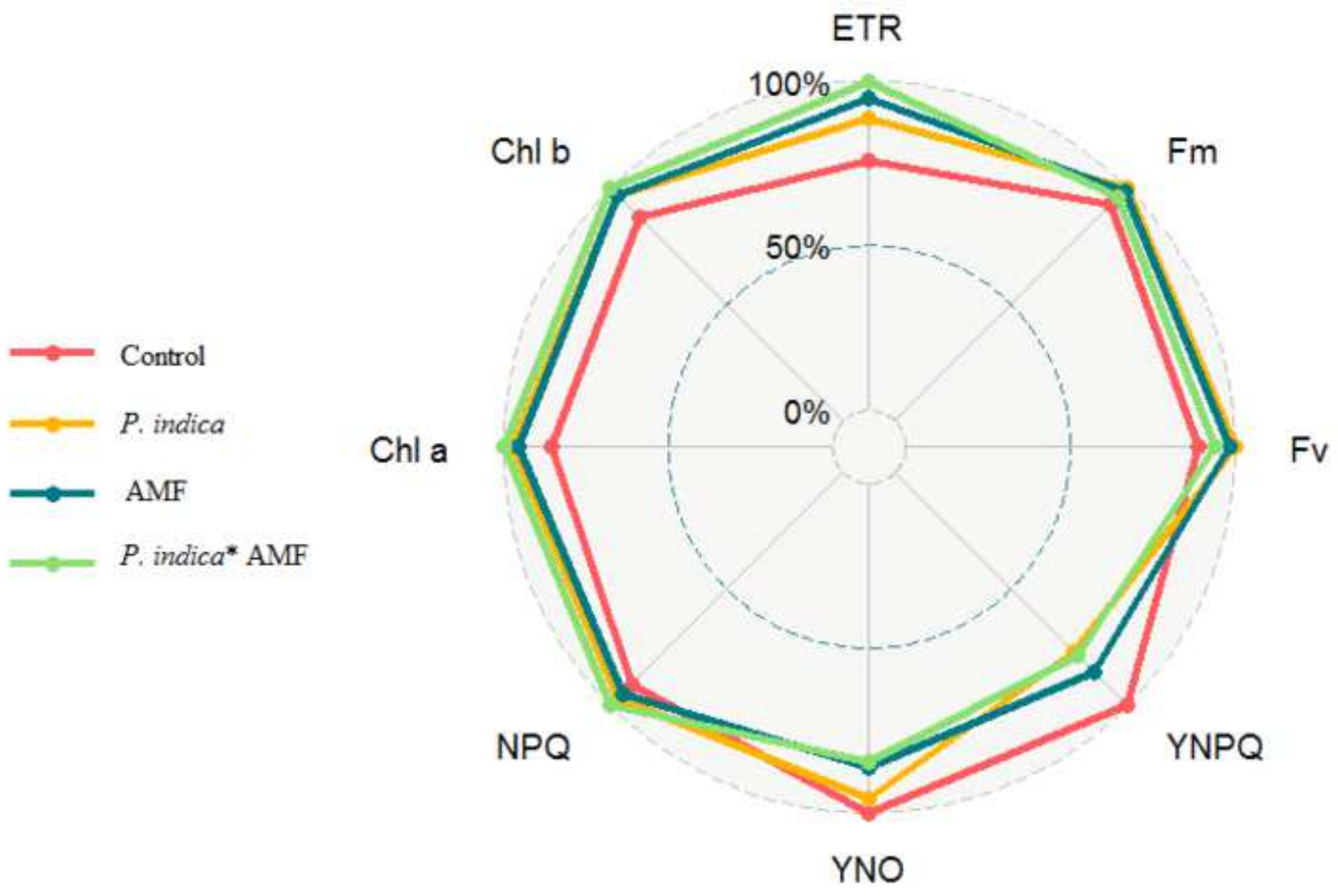

Figure

Click here to access/download;Figure;6b.tif

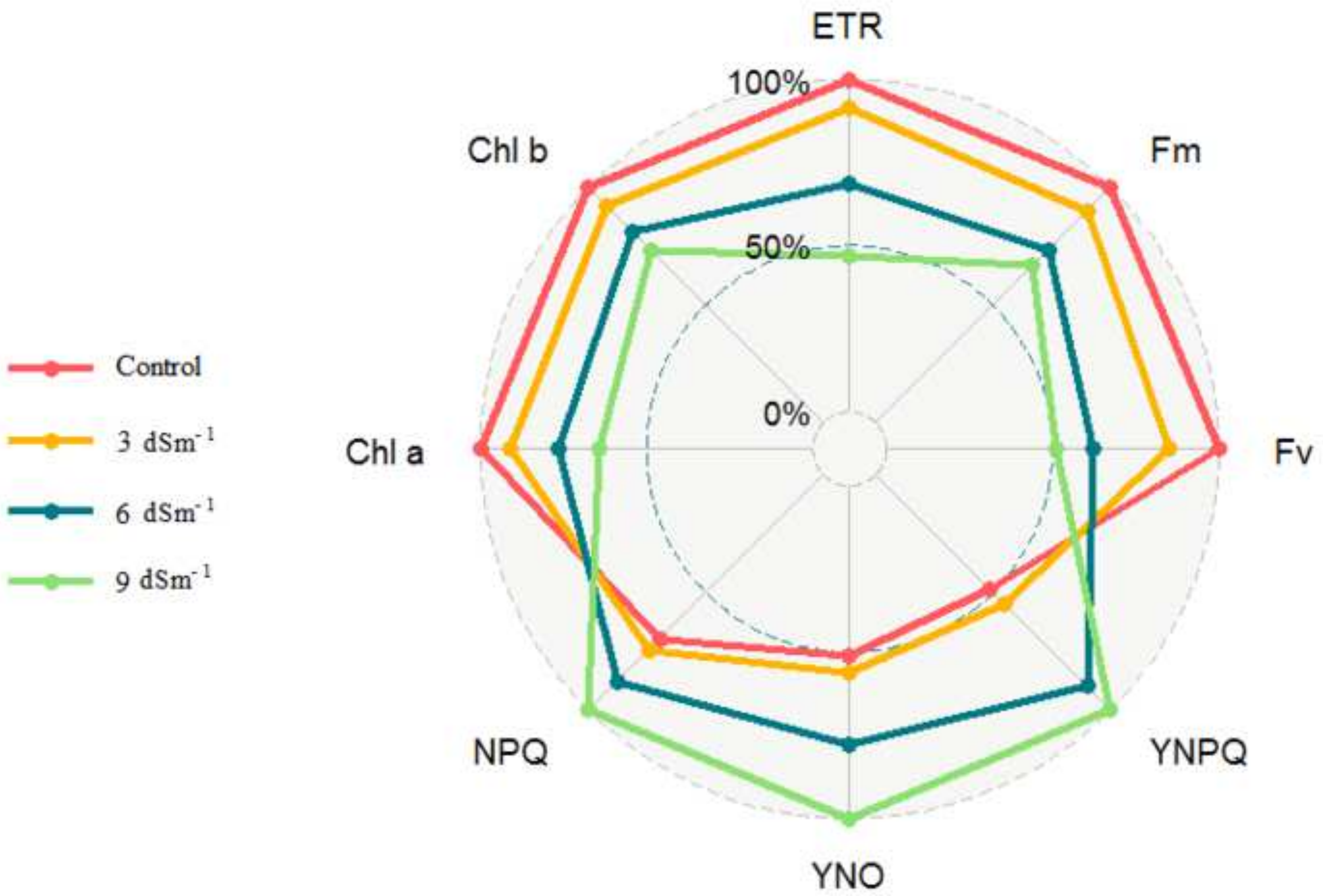

Figure

Click here to access/download;Figure;10.tif

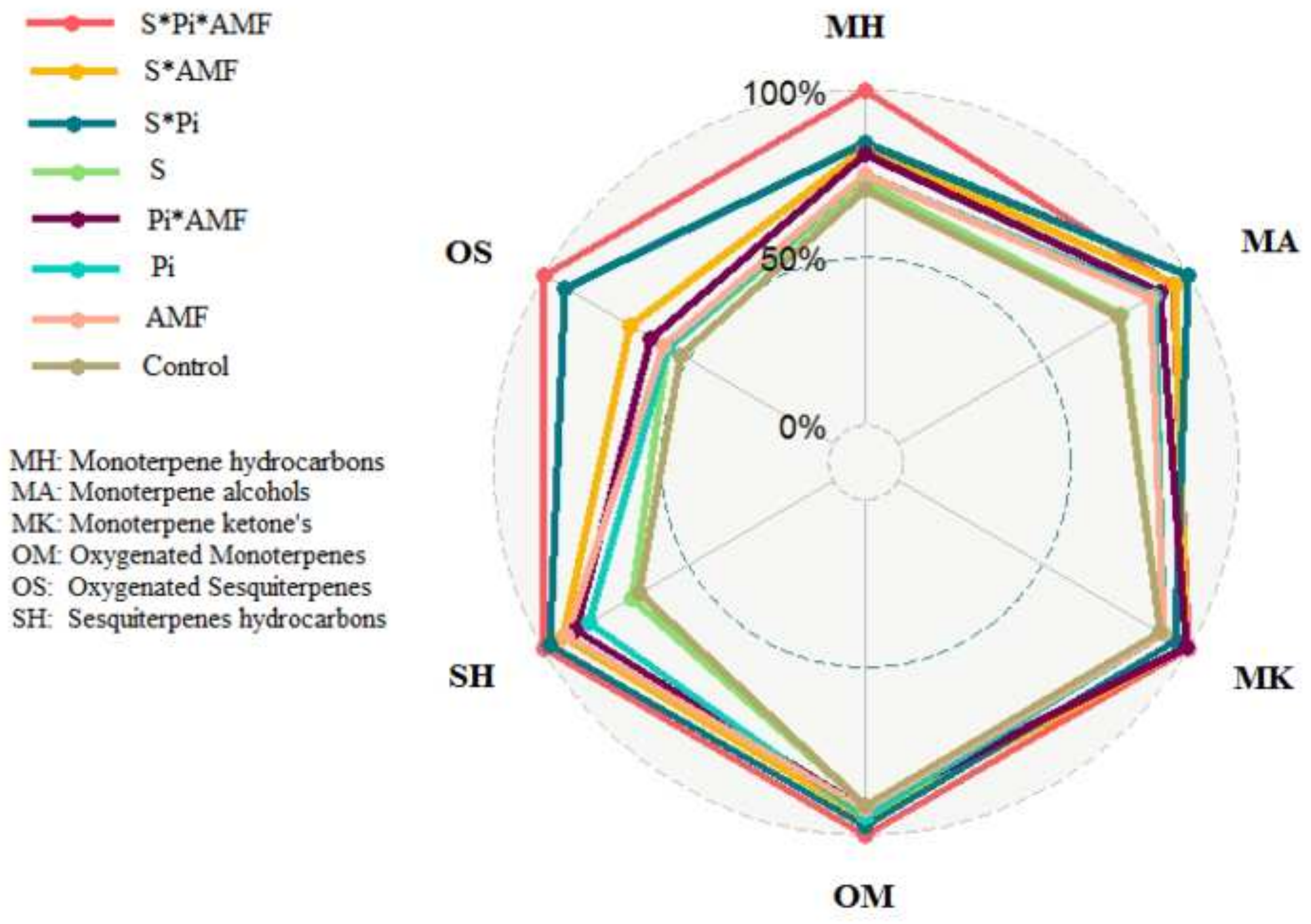

Figure
Click here to access/download;Figure;11.tif

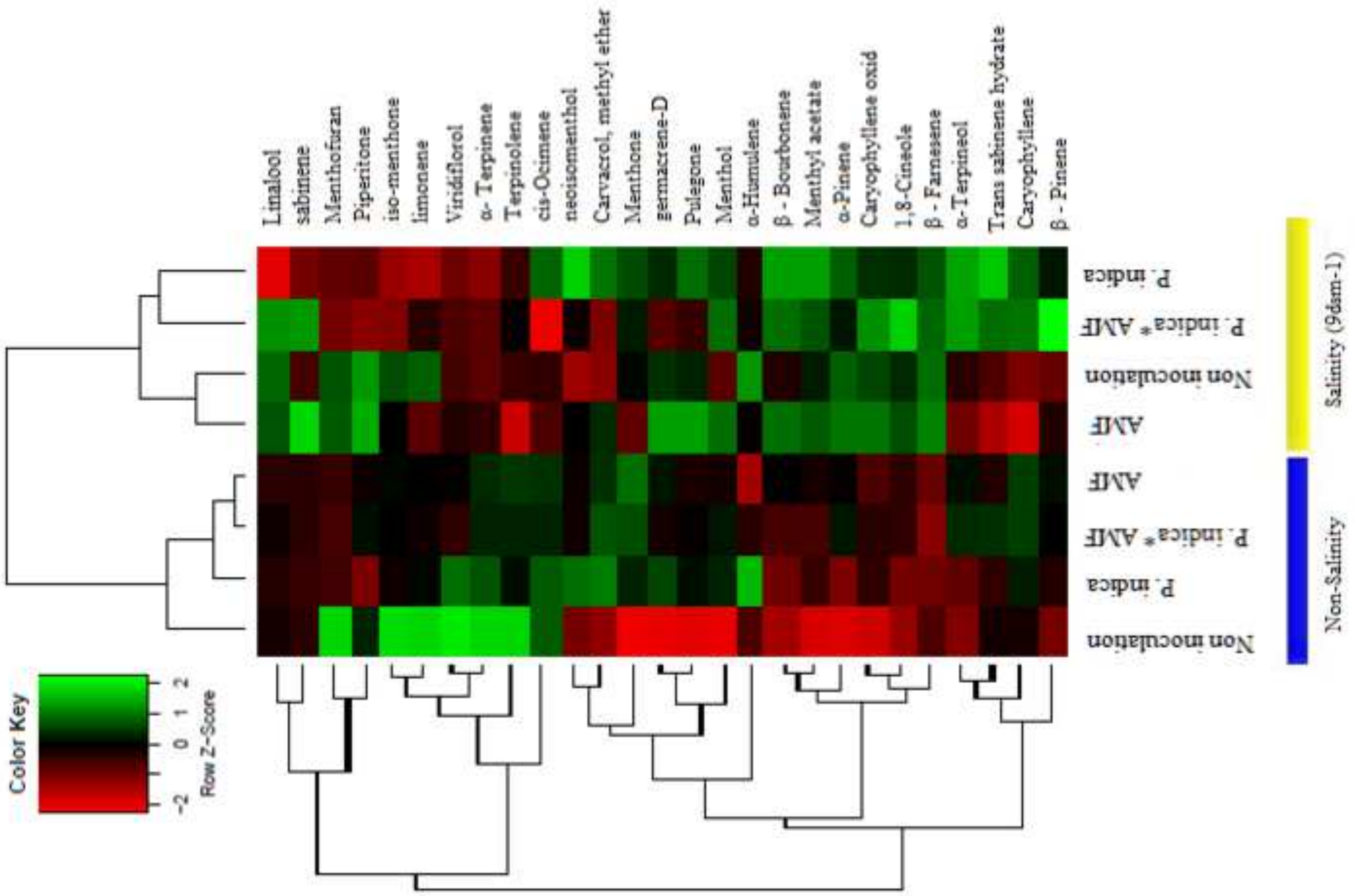

4c

Click here to access/download;Figure;4c.tiff

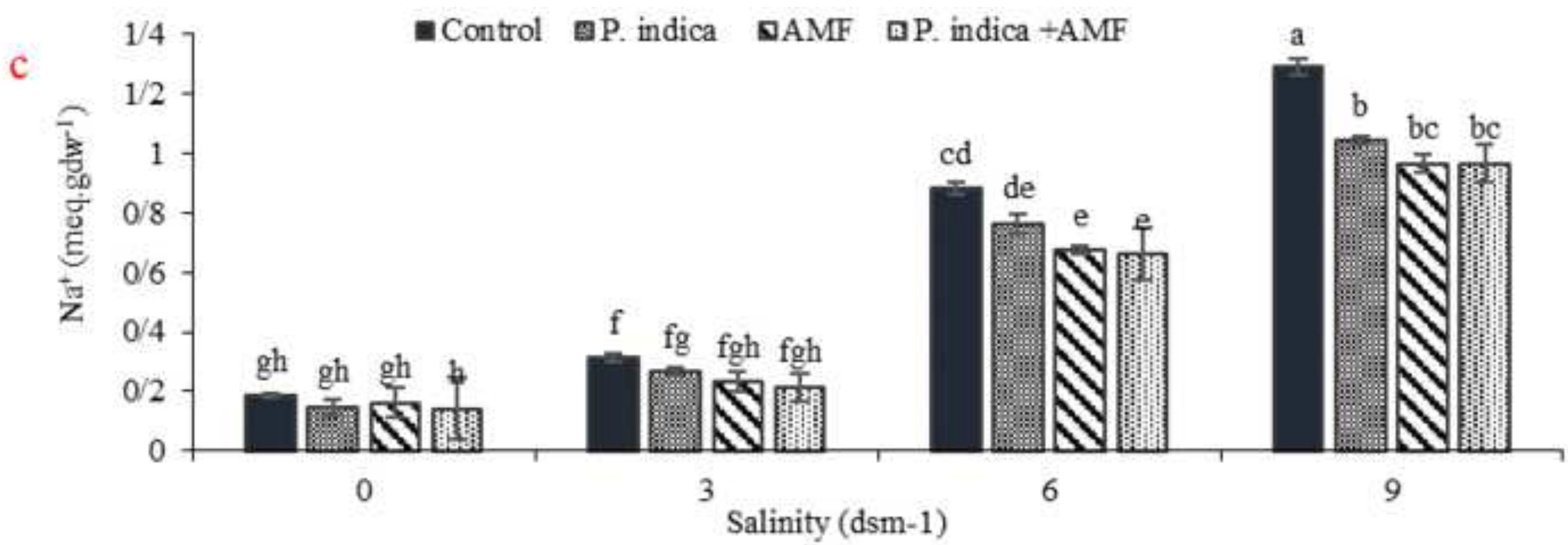

5a

Click here to access/download;Figure;5a.tiff

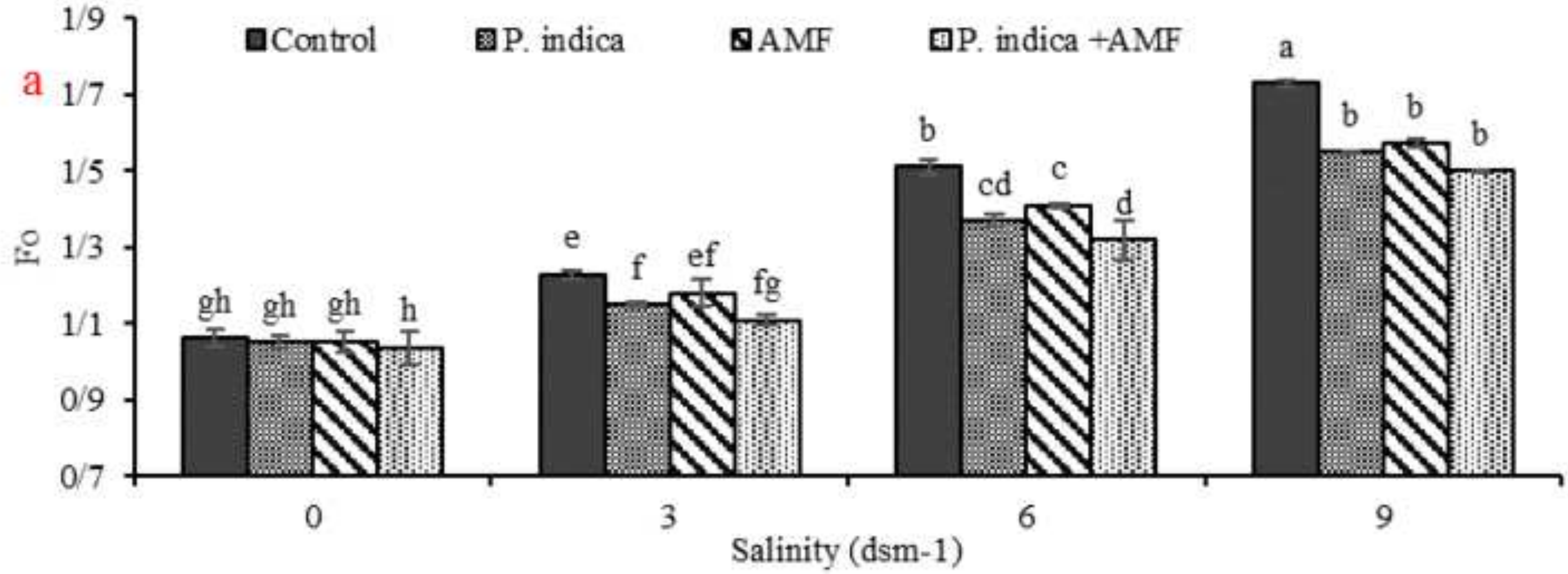

5b

Click here to access/download;Figure;5b.tiff

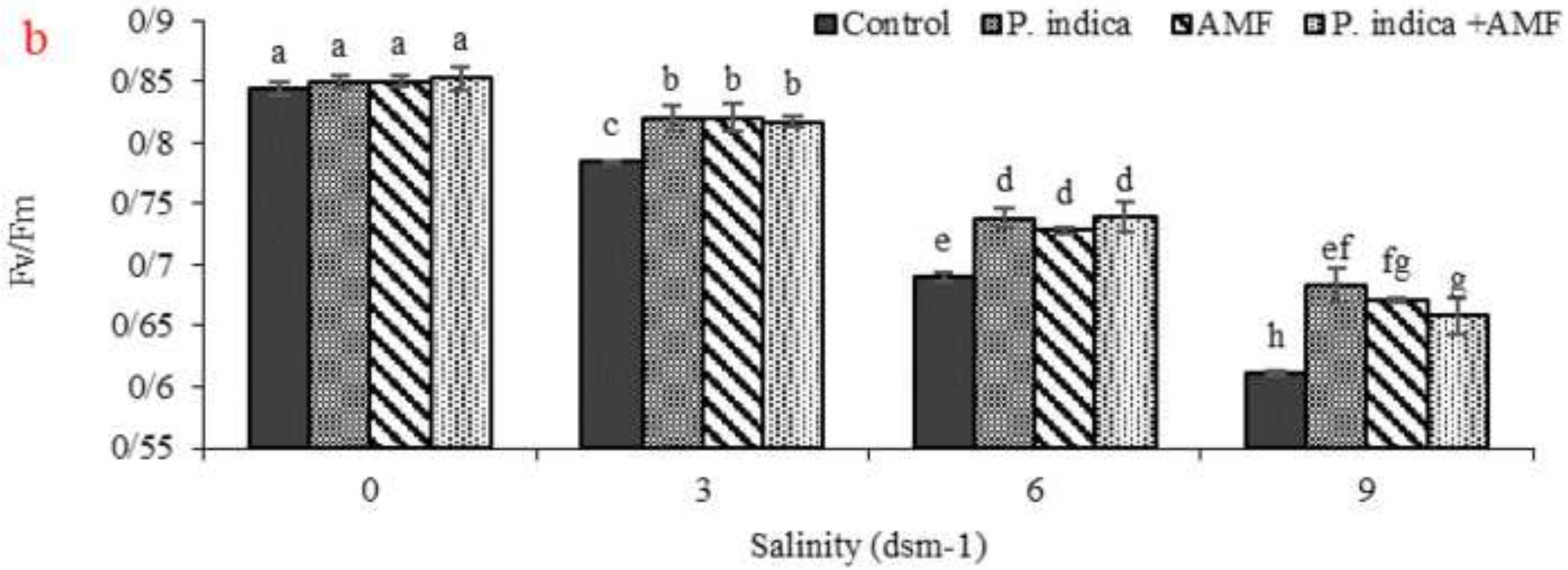

5c

Click here to access/download;Figure;5c.tiff

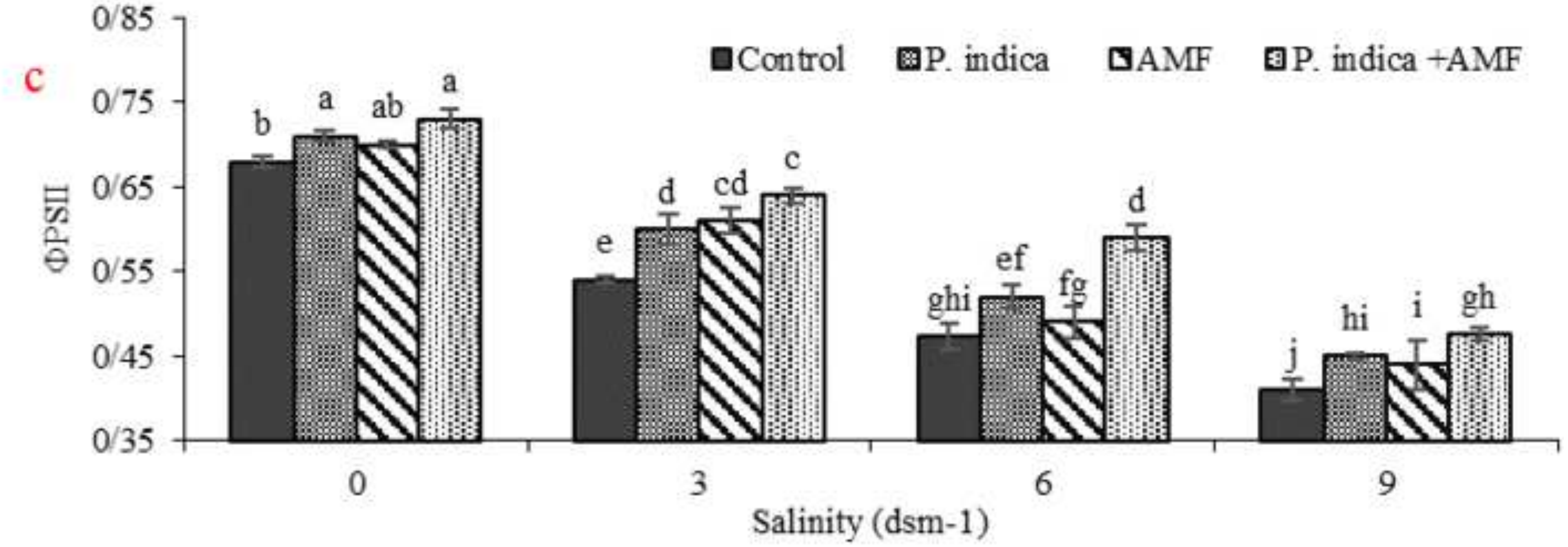

Click here to access/download;Figure;5d.tiff

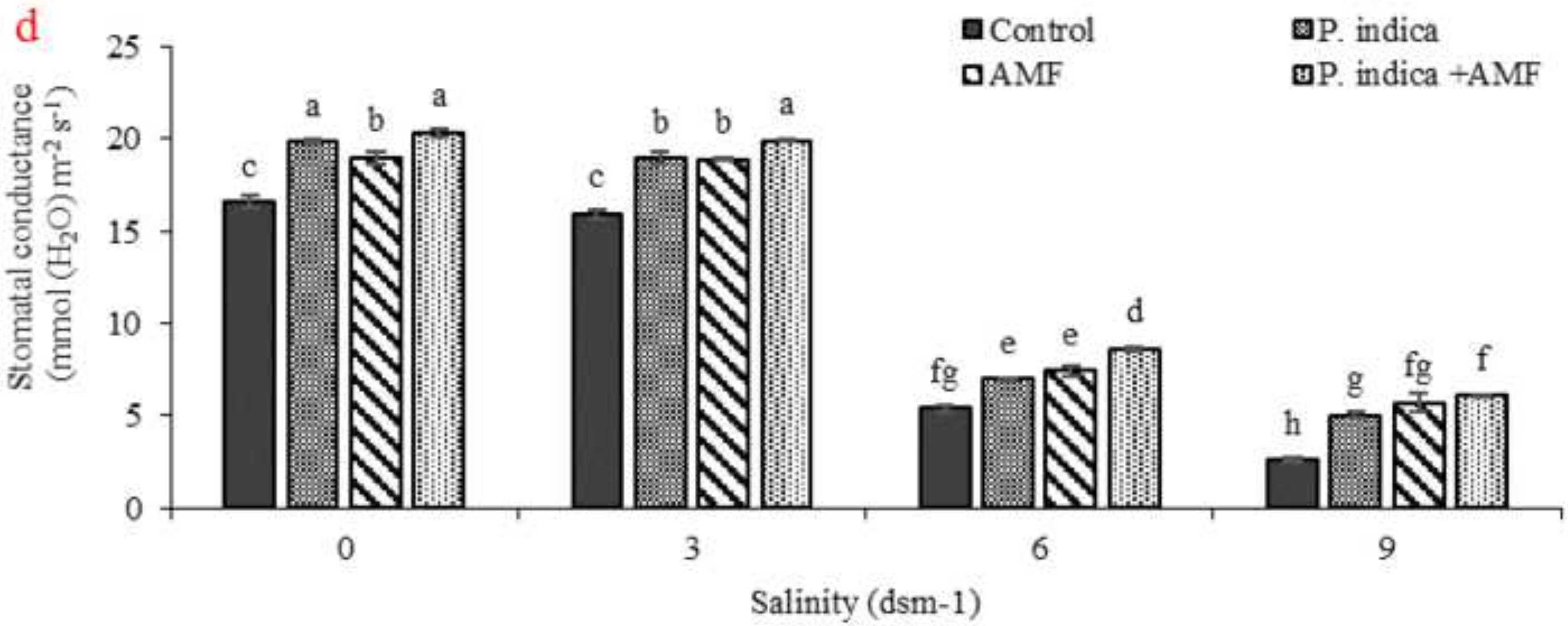

7a

Click here to access/download;Figure;7a.tif

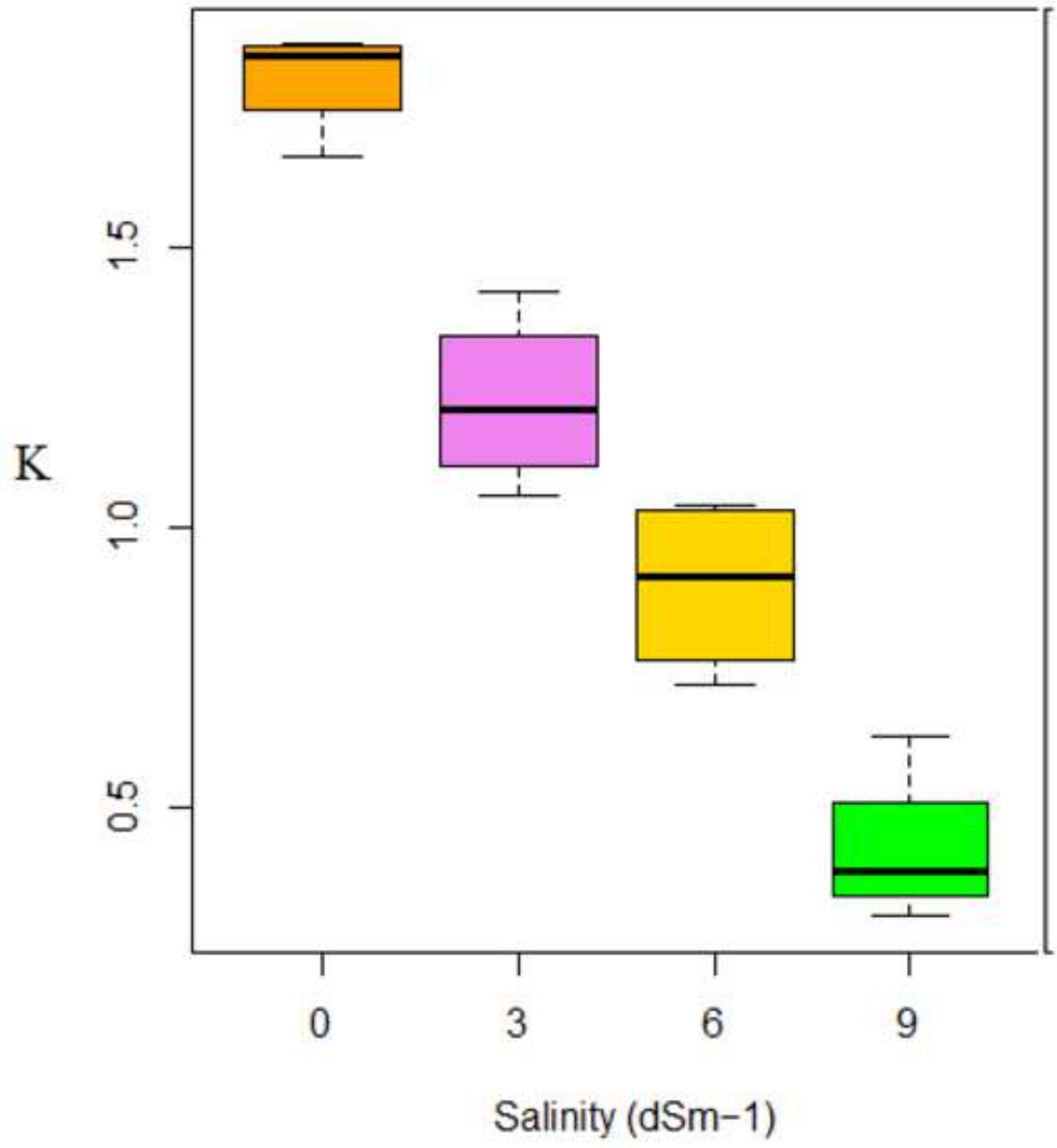

7b

Click here to access/download;Figure;7b.tif

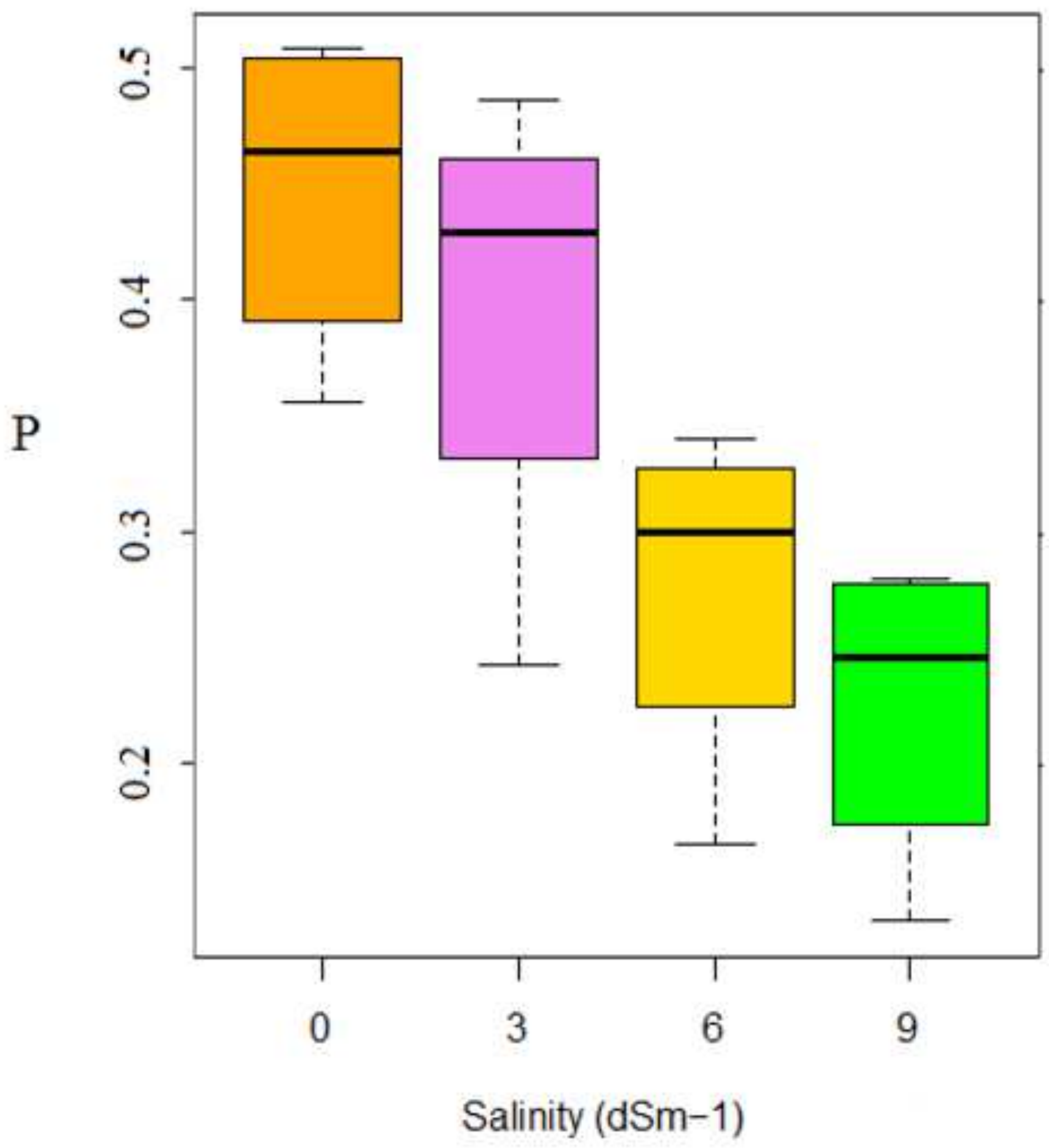

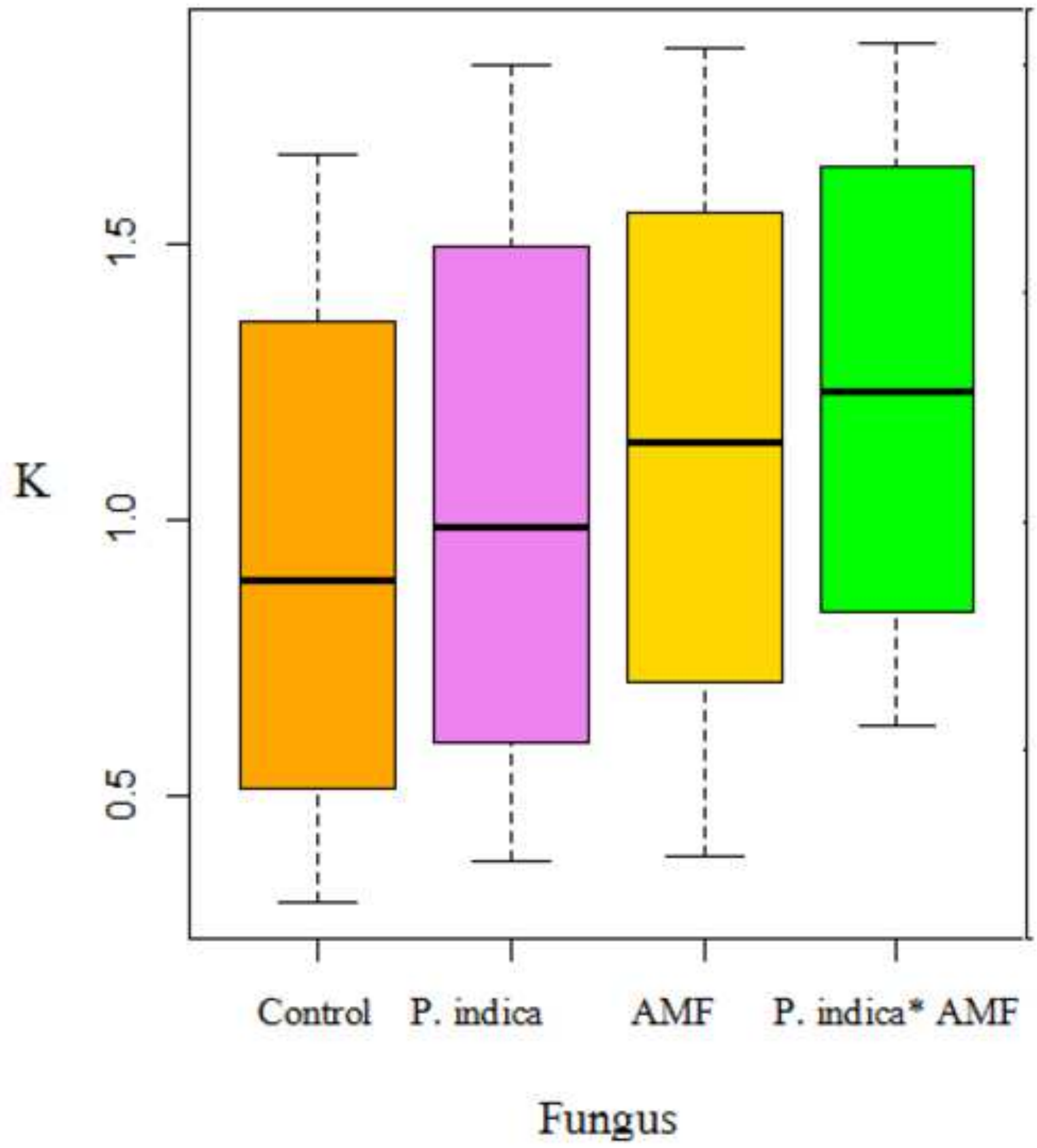
K
0.5
1.0
1.5
Control
P. indica
AMF
P. indica* AMF
Fungus

Click here to access/download;Figure;7d.tif

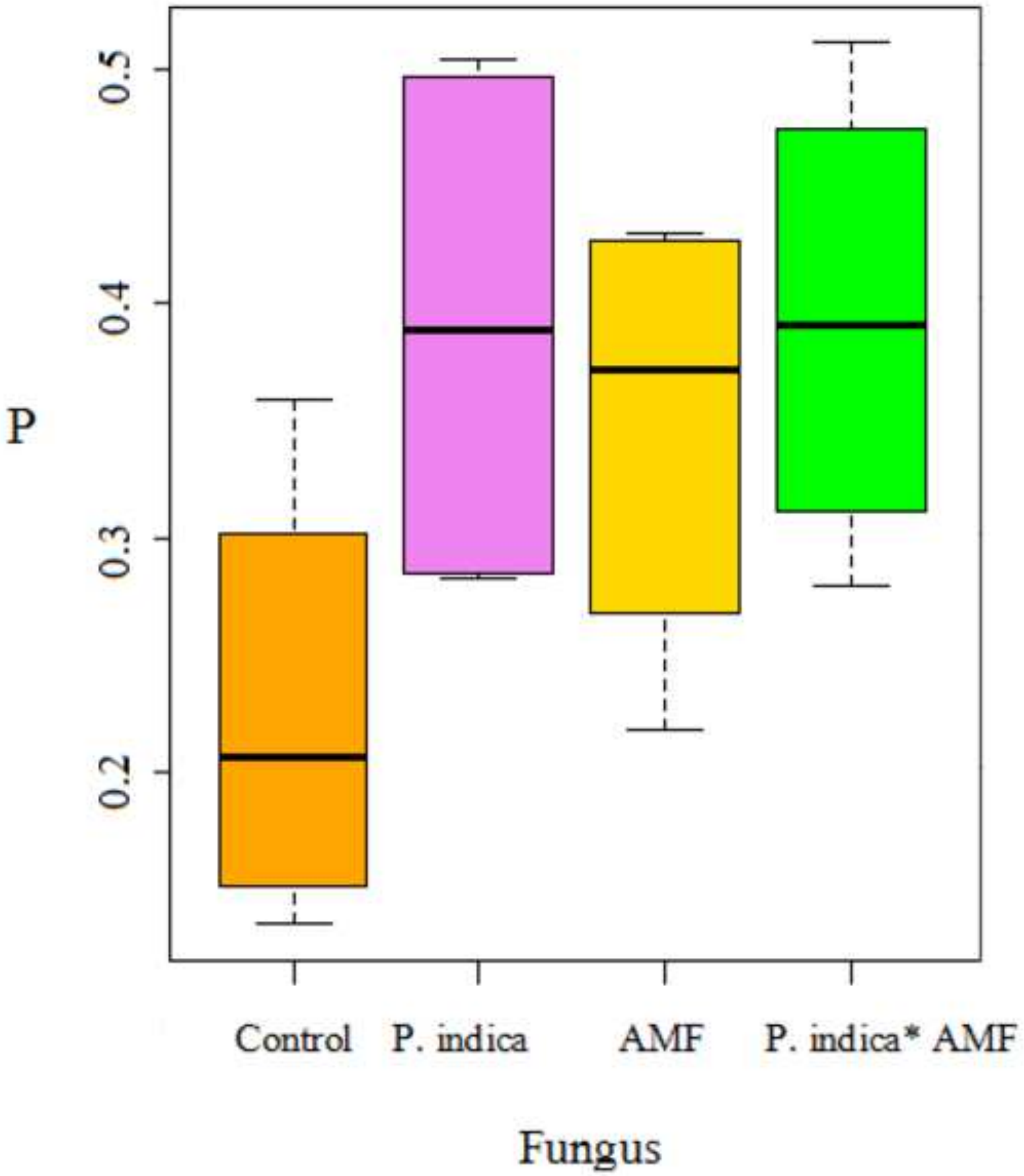

Click here to access/download;Figure;8a.tiff

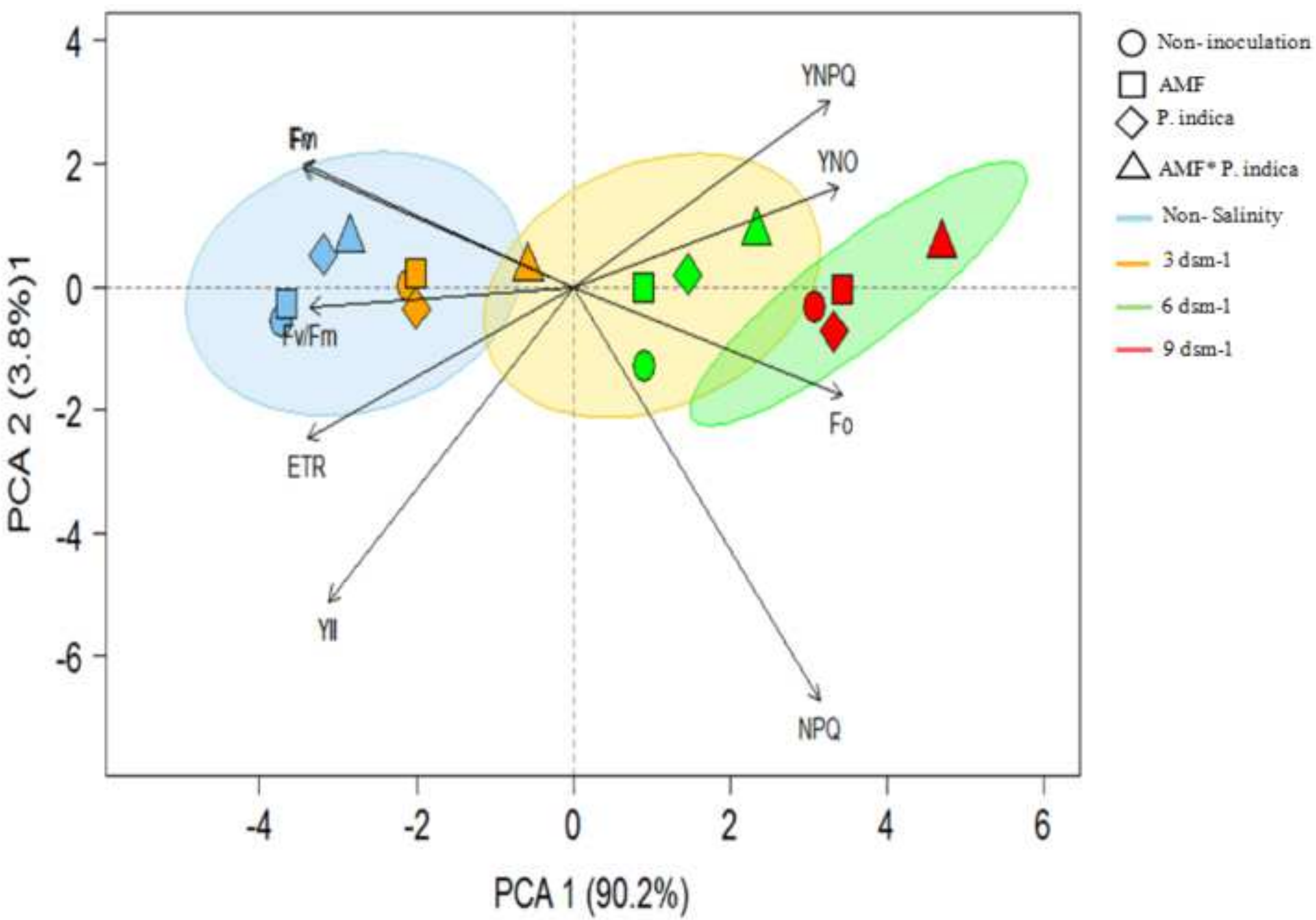

8b

Click here to access/download;Figure;8b.tiff

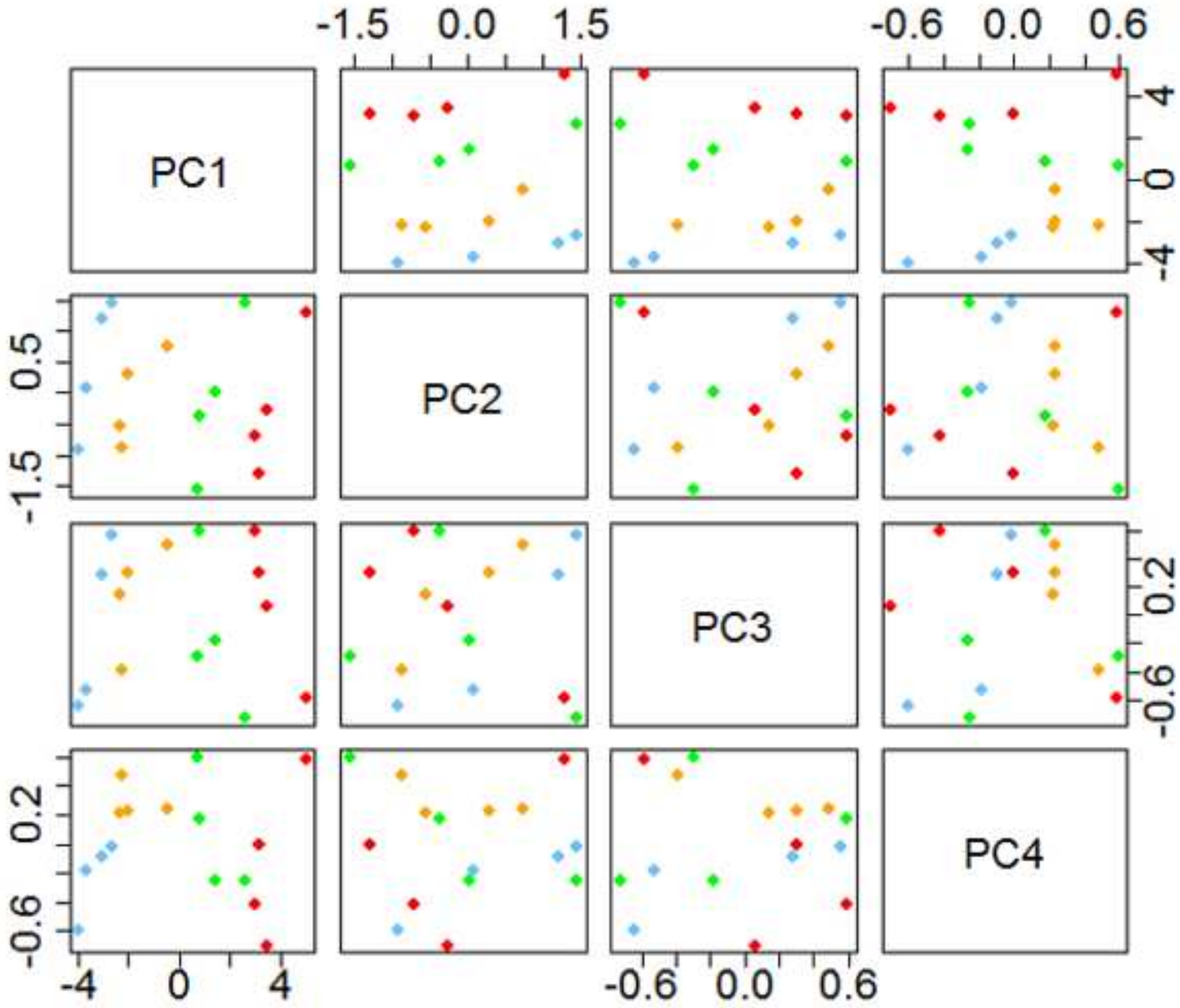

Click here to access/download;Figure;9.tiff

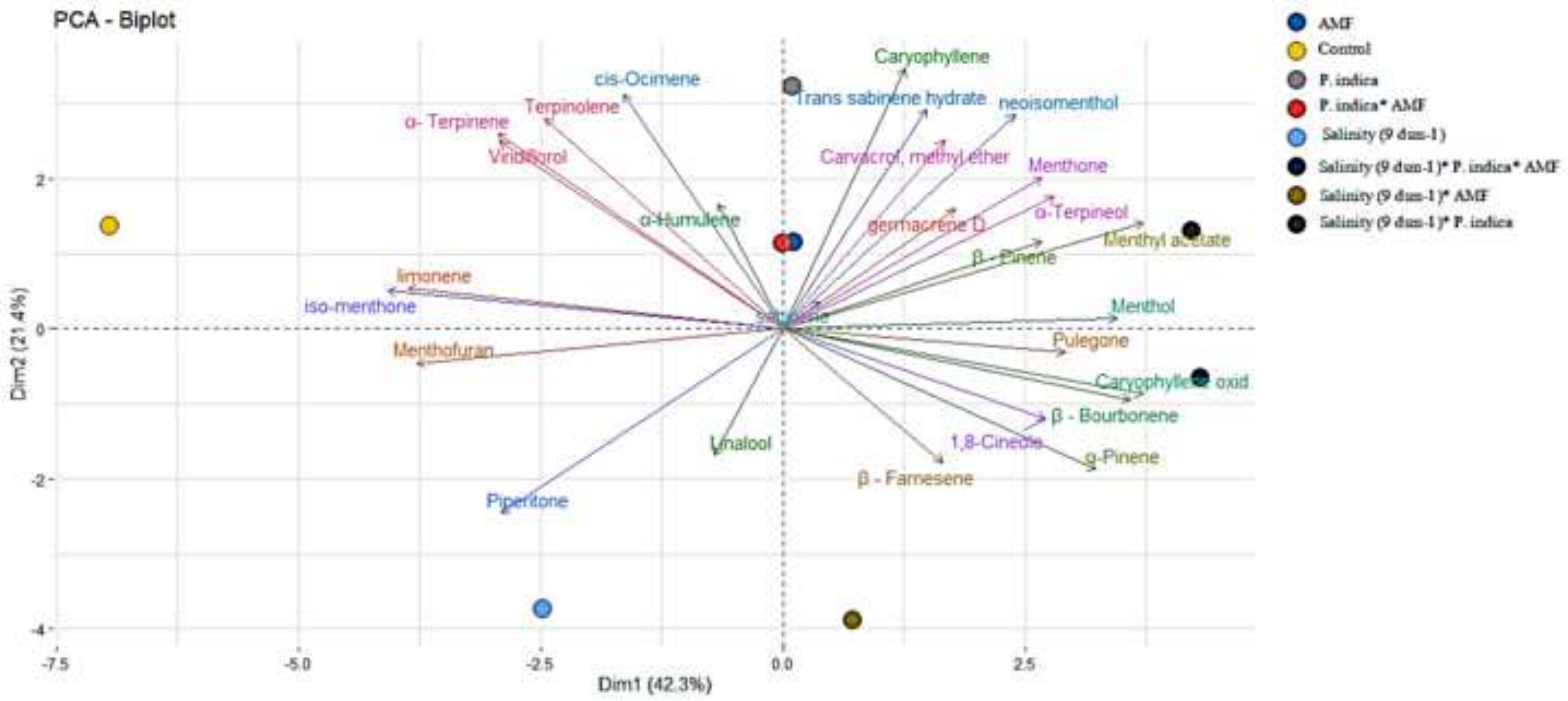

12a

Click here to access/download;Figure;12a.tiff

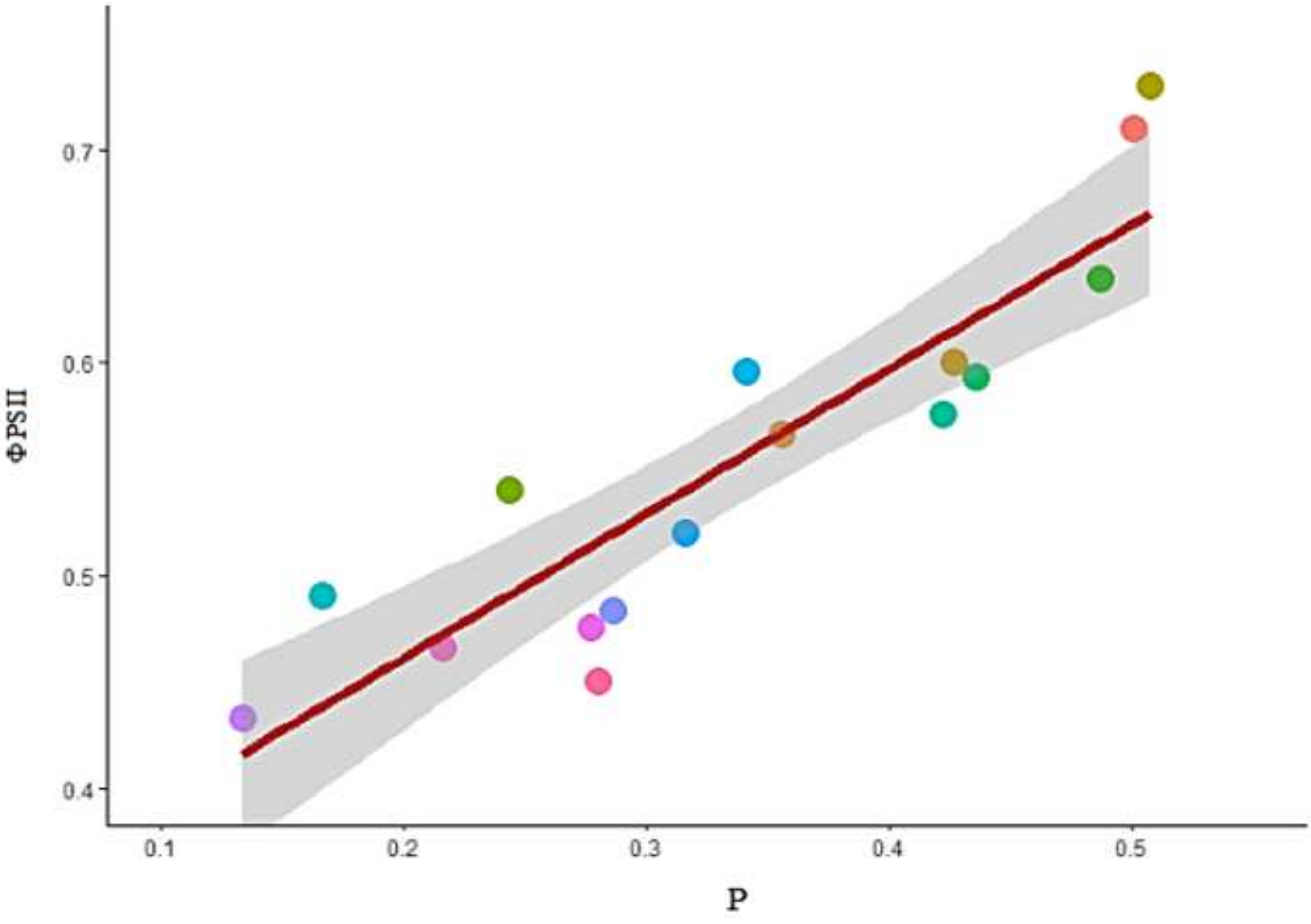

12b

Click here to access/download;Figure;12b.tiff

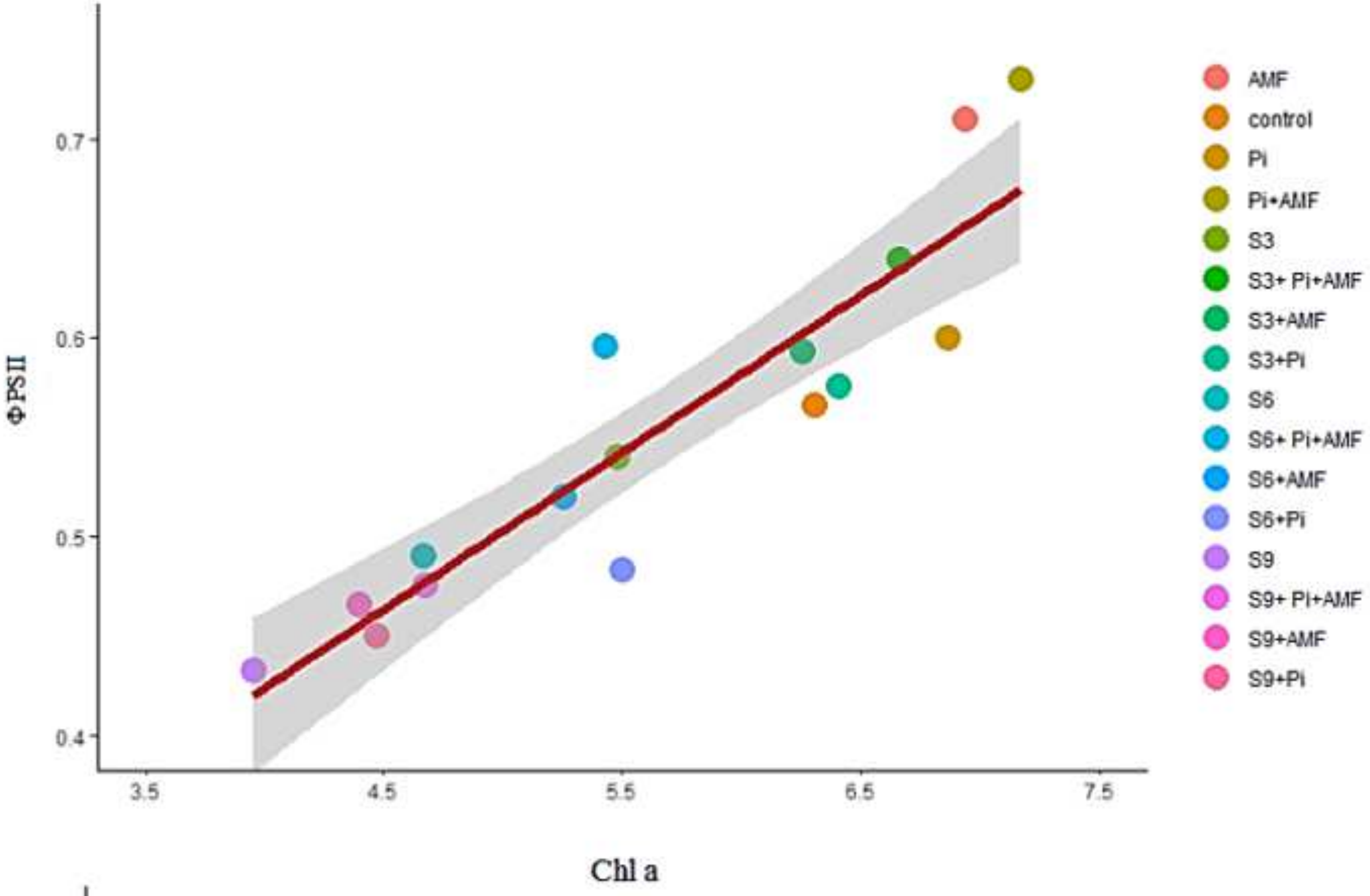

Click here to access/download;Figure;12c.tiff

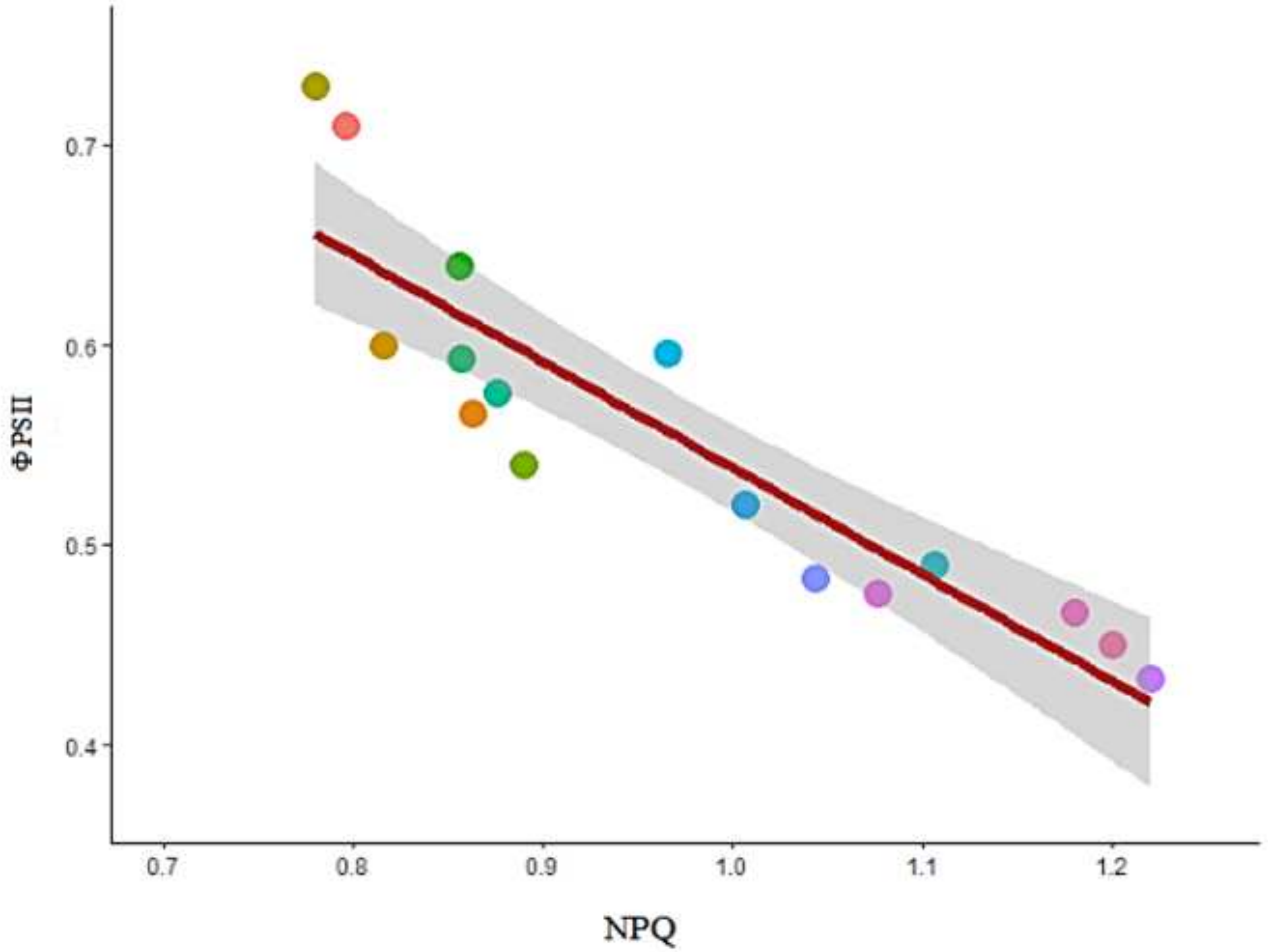

12d

Click here to access/download;Figure;12d.tiff

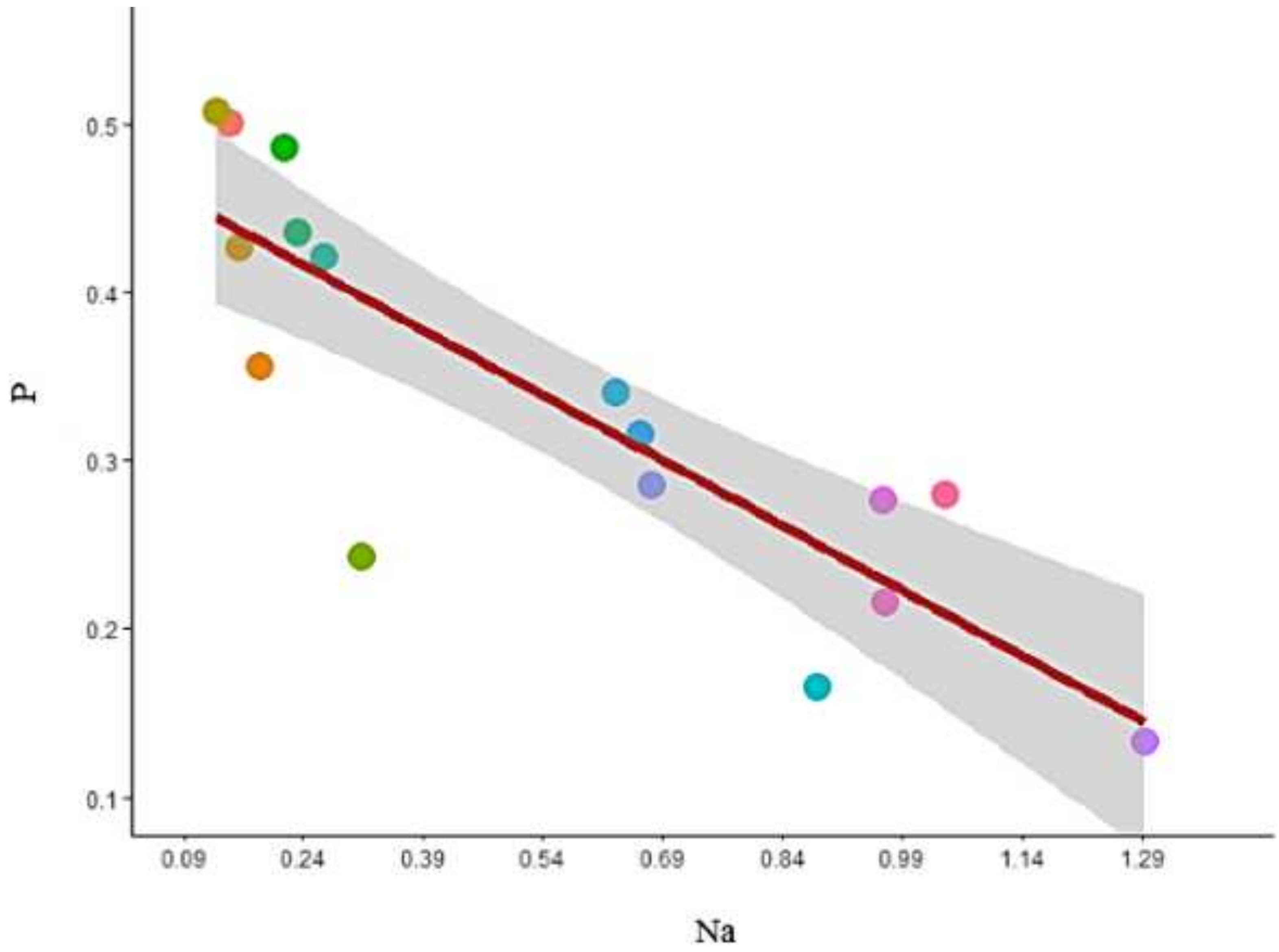

 Click here to access/download;Figure;13a.tiff

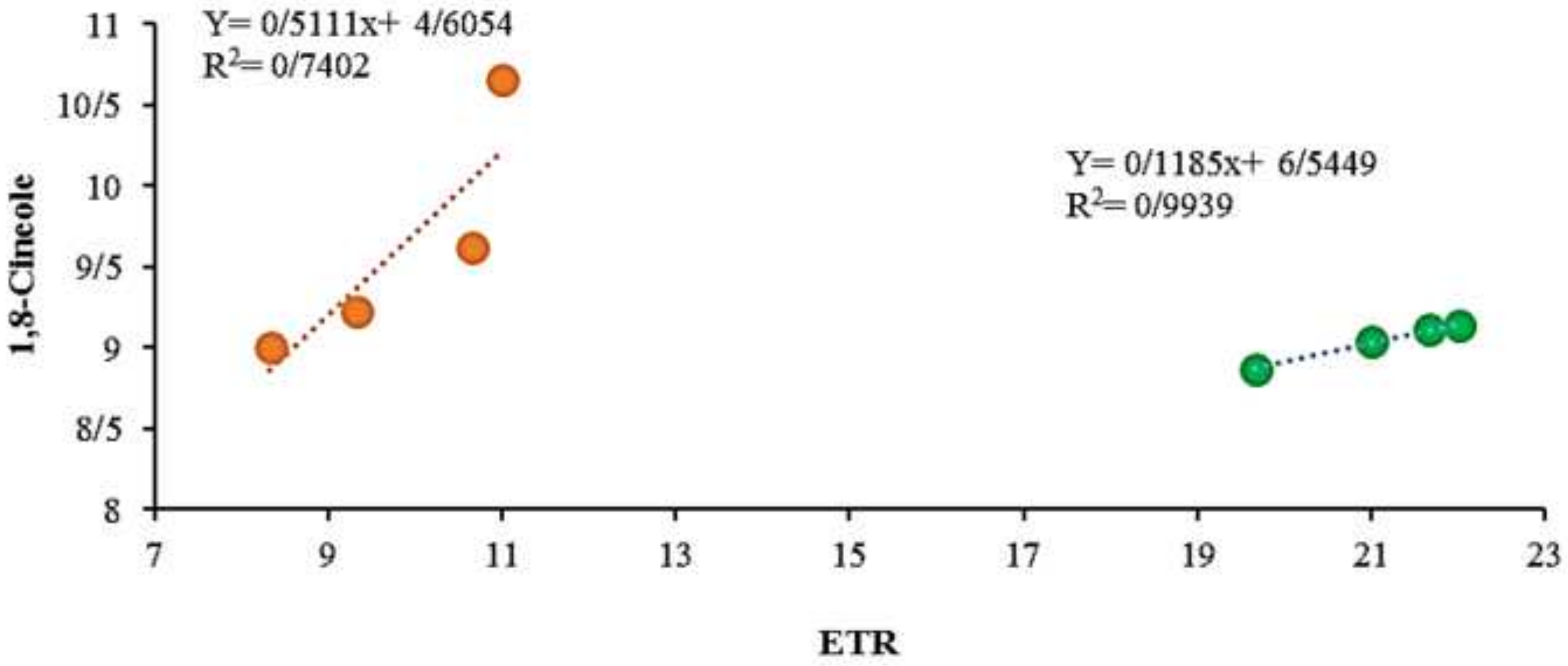

 Click here to access/download;Figure;13b.tiff

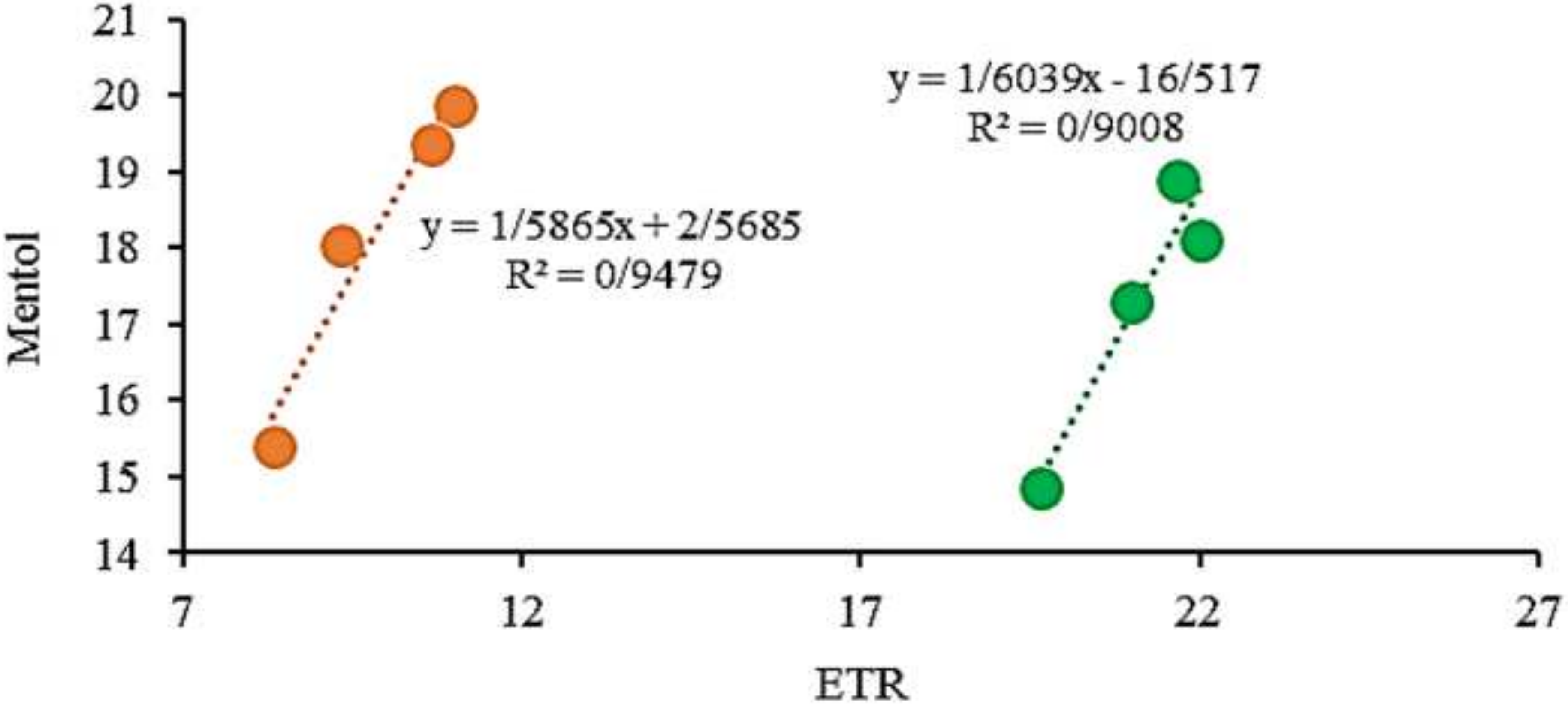

Click here to access/download;Figure;Fig 3..tiff

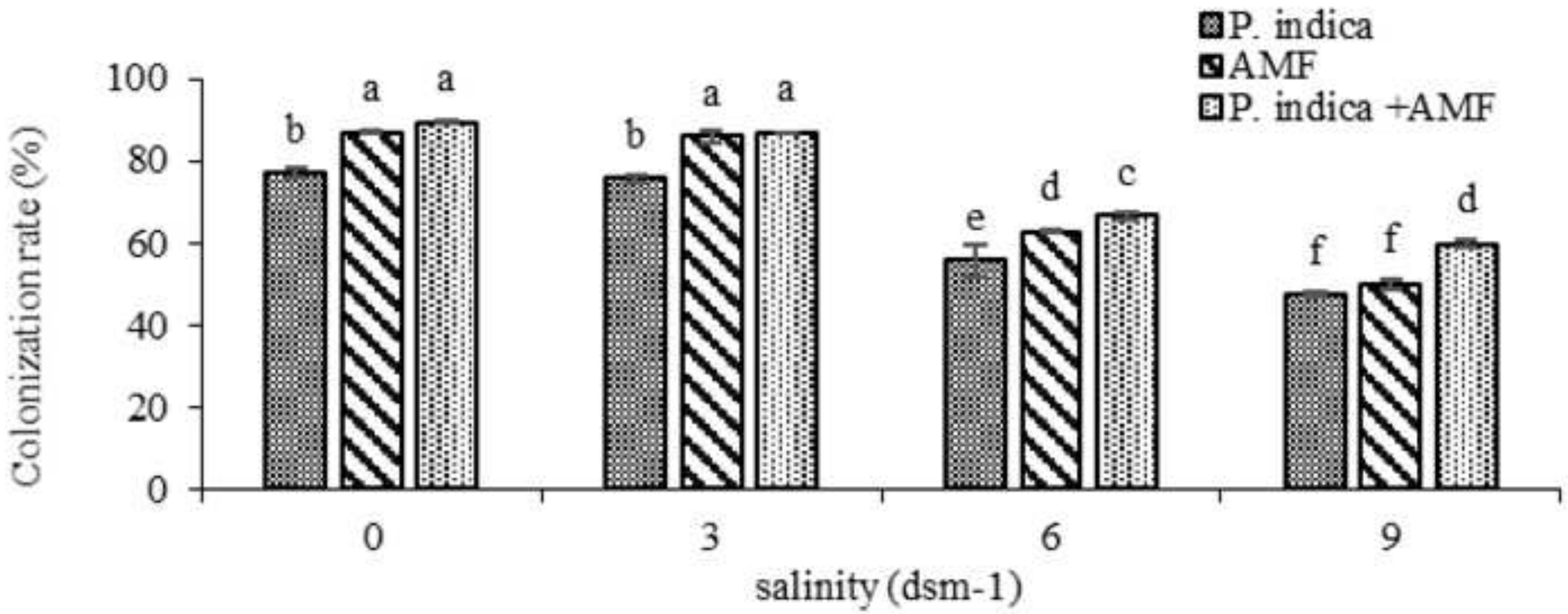

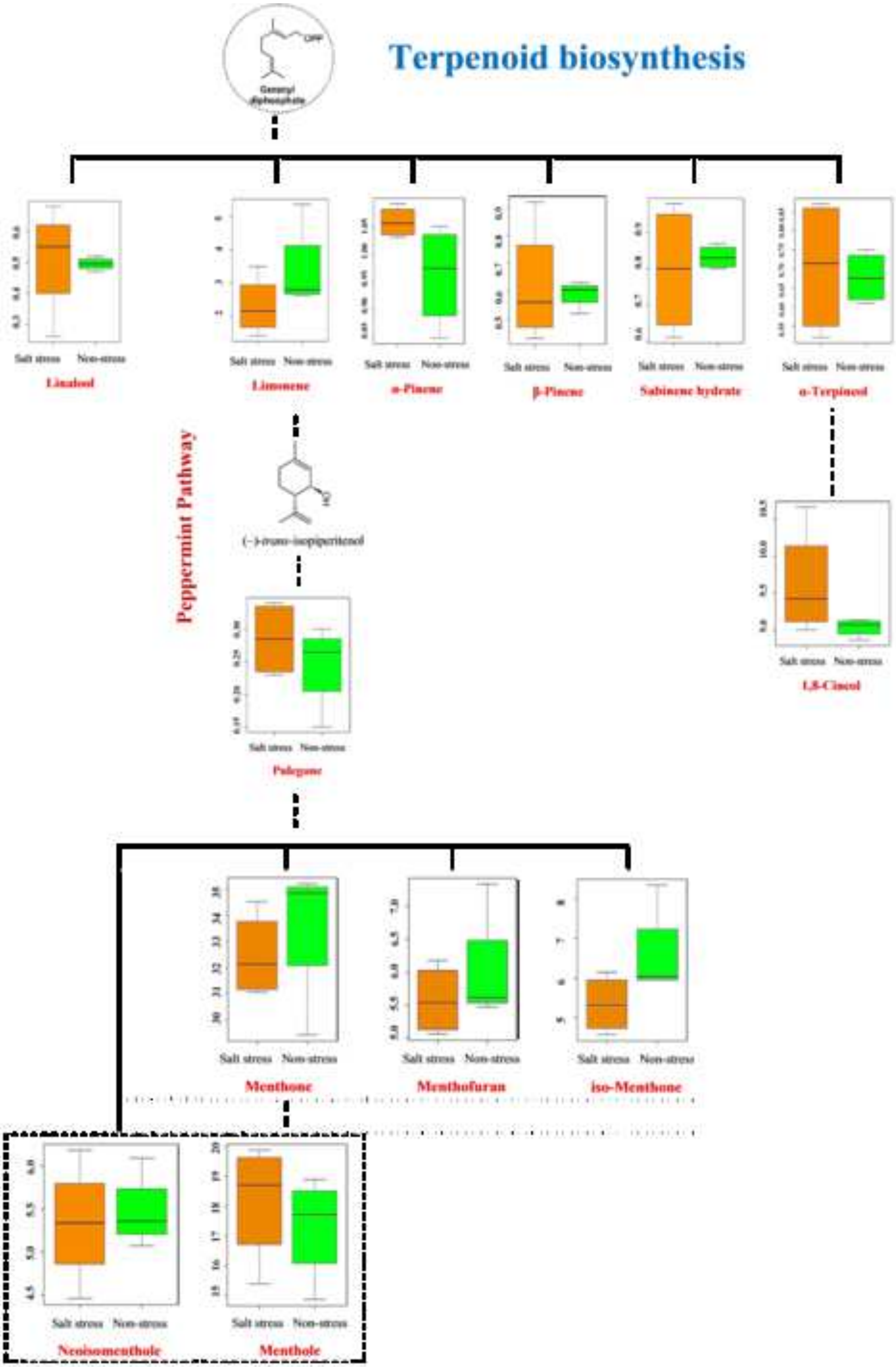
Terpenoid biosynthesis
Geranyl diphosphate
OPP
Salt stress
Non-stress
Linalool
Limonene
α-Pinene
β-Pinene
Sabinene hydrate
α-Terpineol
Peppermint Pathway
(−)-trans-isopiperitenol
1,8-Cineol
Pulegone
Menthone
Menthofuran
iso-Menthone
Neoisomenthole
Menthole

4a

Click here to access/download;Figure;Fig 4a.tiff

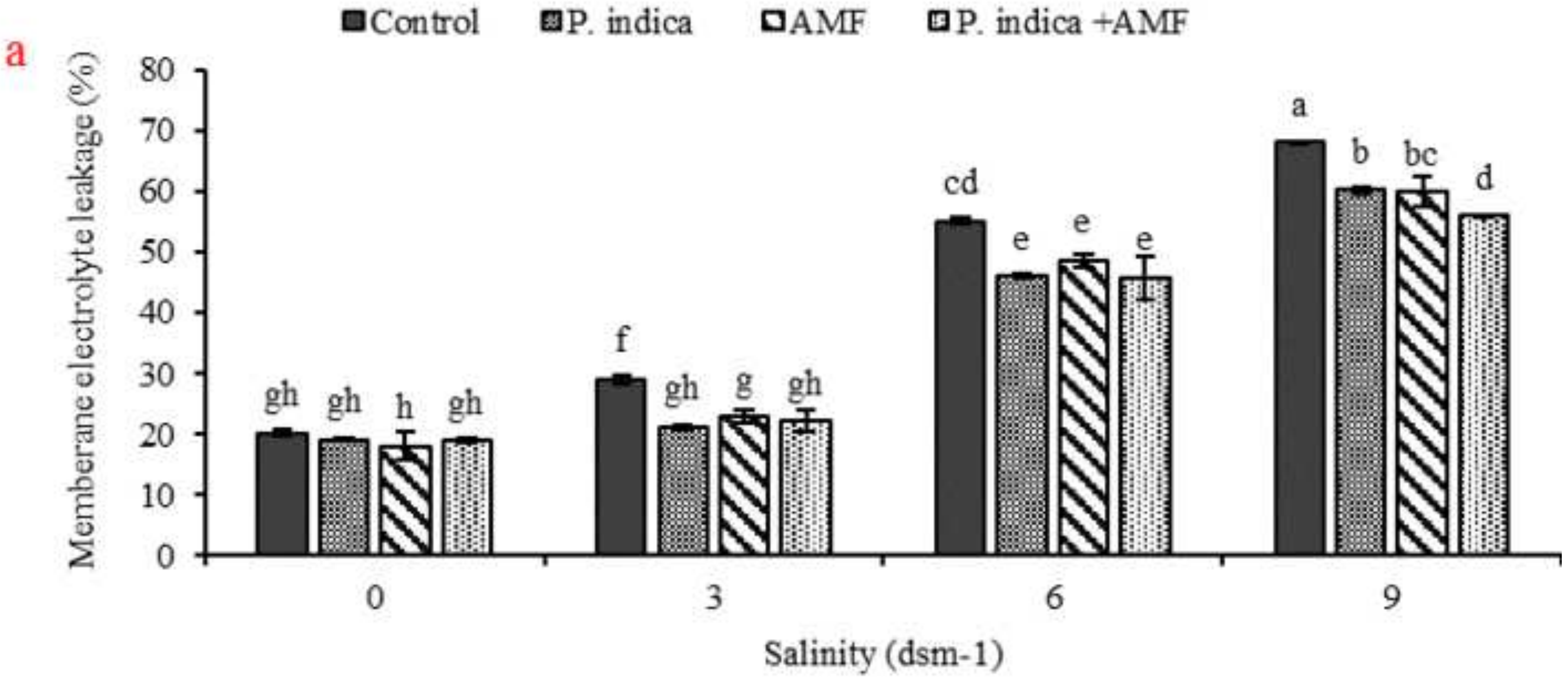

4b

Click here to access/download;Figure;Fig 4b.tiff

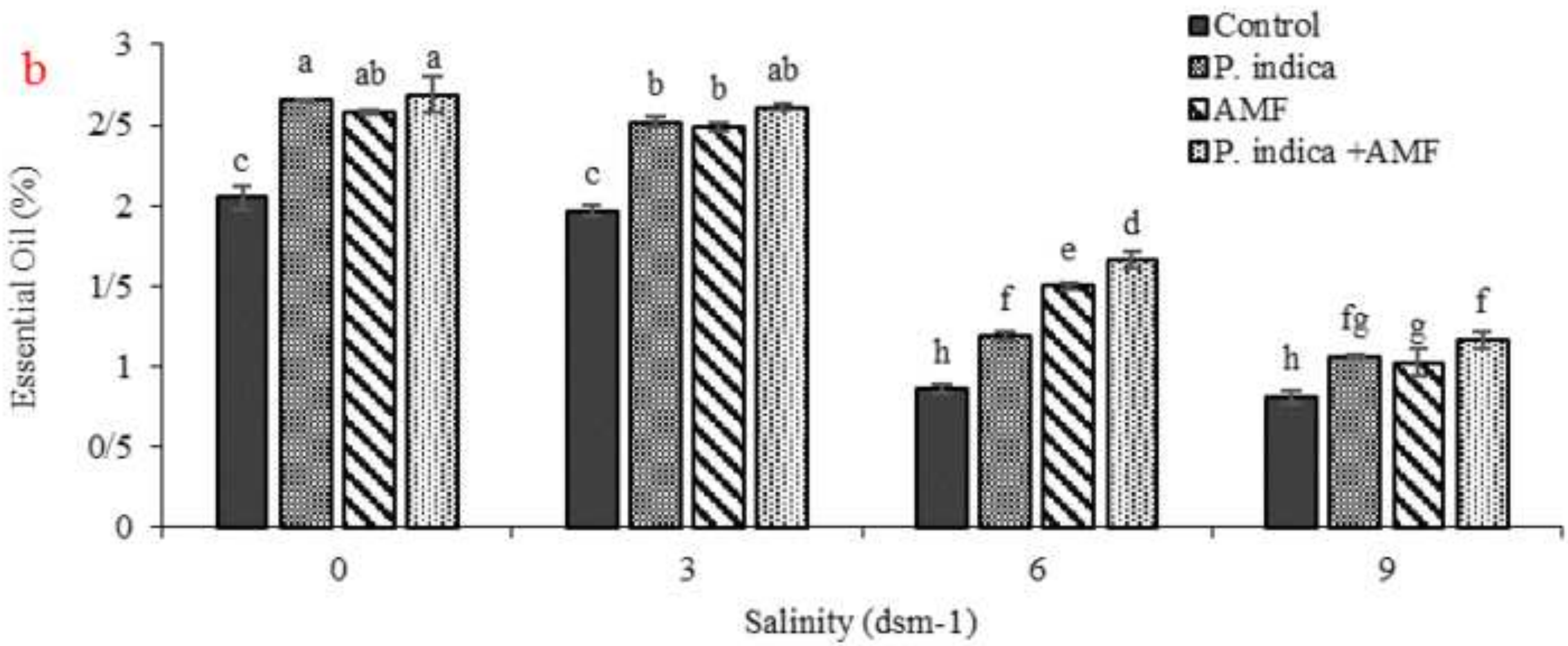

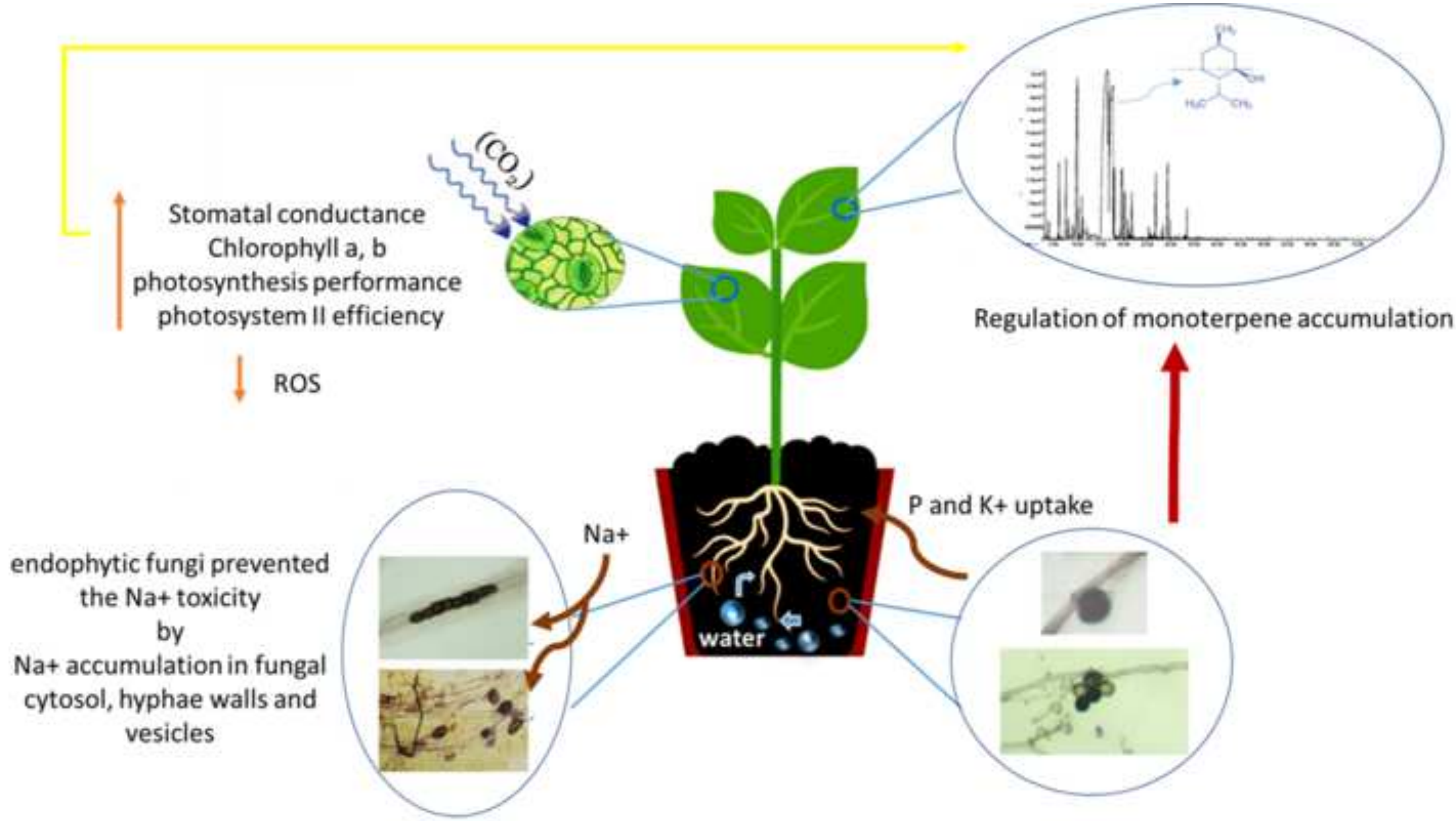
(CO2)
Stomatal conductance
Chlorophyll a, b
photosynthesis performance
photosystem II efficiency
ROS
Regulation of monoterpene accumulation
P and K+ uptake
Na+
water
endophytic fungi prevented
the Na+ toxicity
by
Na+ accumulation in fungal
cytosol, hyphae walls and
vesicles

Graph of essential oil

Click here to access/download;Figure;Graph of essential oil.tiff

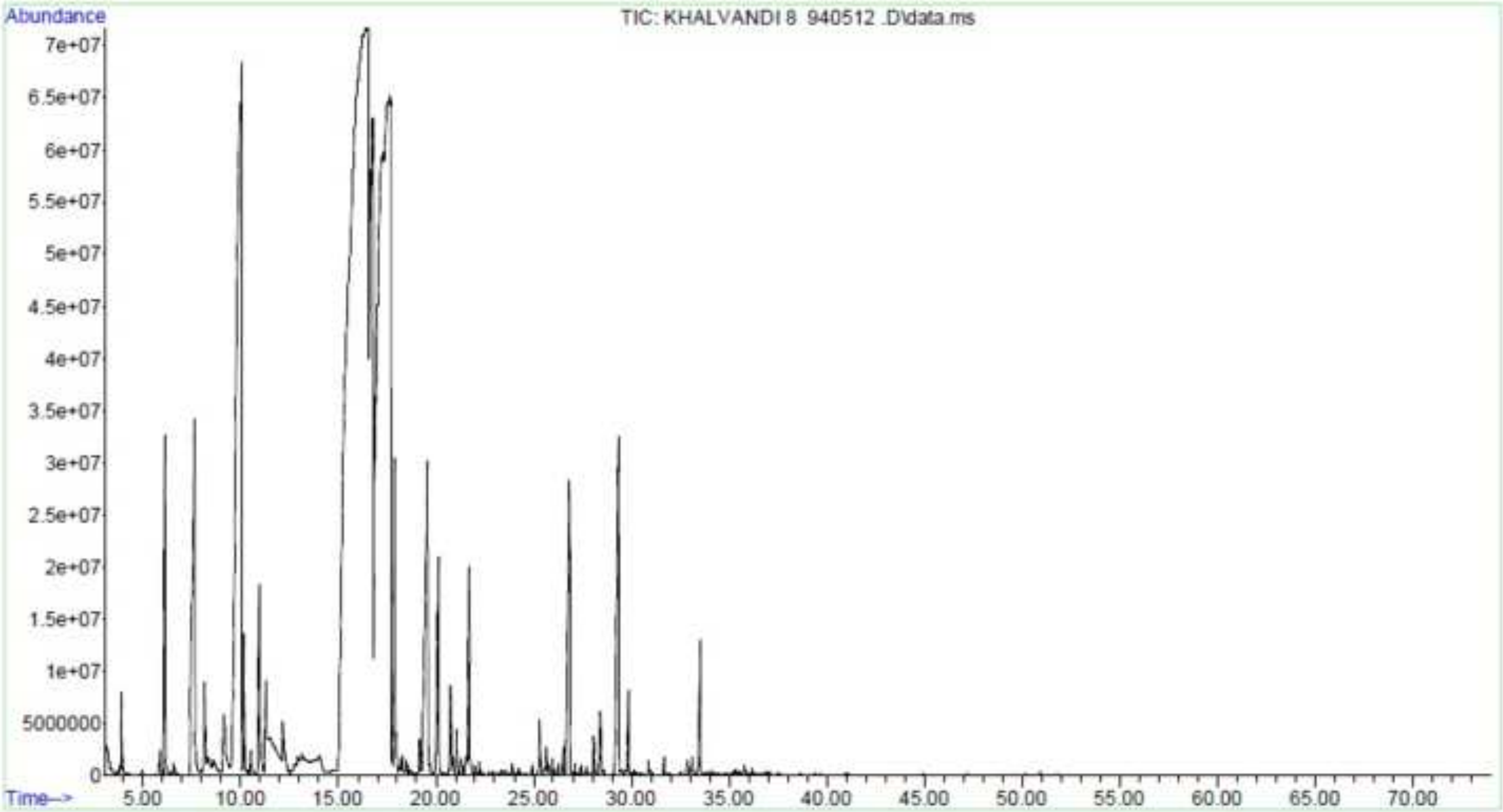

Click here to access/download
Supporting Information
S1.xlsx

Click here to access/download
Supporting Information
S2.xlsx

Click here to access/download
Supporting Information
S3.xlsx

Does co-inoculation of mycorrhiza and *Piriformospora indica* fungi enhance the efficiency of Chlorophyll fluorescence and essential oil composition in peppermint under irrigation with saline water from the Caspian Sea?

Masoumeh Khalvandi[1*], Mohammadreza Amerian[2], Hematollah Pirdashti[3], Sara Keramati[3]

[1] Ph.D. Graduate, Department of Agronomy, Faculty of Agriculture, Shahrood University of Technology, Iran.

[2] Department of Agronomy, Faculty of Agriculture, Shahrood University of Technology, Iran

[3] Department of Agronomy, Genetic and Agricultural Biotechnology Institute of Tabarestan, Sari Agricultural Sciences and Natural Resources University, Iran

[*] Corresponding author
E-mail: M.khalvandi@gmail.com

Abstract

Symbiotic associations with endophytic fungi are ecologically important for medicinal and aromatic plants. Endophytic fungi highly affect the quantity and quality of herbal products. In this study, a pot experiment was carried out in the greenhouse to investigate the interactive effects of *Piriformospora indica* and arbuscular mycorrhizal (AMF) inoculation on the chlorophyll fluorescence, essential oil composition, and antioxidant enzymes of peppermint under saline condition. The results showed that Fo, YNPQ, YNO, and NPQ values were obviously increased under salinity conditions, while essential oil content, chlorophyll a and b, gs, Fm, Fv, ETR, ΦPSII and Fv/Fm ratio decreased by increasing salinity. In addition, salt induced the excess Na^+ uptake, whereas the opposite trend was observed for P and K^+. The synergistic association of *P. indica* and AMF caused a considerable increase in the antioxidant ability, essential oil content, Fv/Fm ratio, ΦPSII, and amount of P and K^+ uptake in salt-stressed plants. The main peppermint oil constituents, menthol, menthone, and 1,8-cineole increased considerably in inoculated plants. Besides, the applied endophytic fungi positively enhanced the ability of peppermint to alleviate the negative effect of the salinity stress.

Keywords: Chlorophyll fluorescence, secondary metabolites, *Piriformospora indica*, arbuscular mycorrhizal, salinity stress.

1. Introduction

Mentha piperita is one of the most important medicinal plants of Lamiaceae family, the demand of which has grown dramatically in recent decades. The essential oil of peppermint is widely used in food, fragrance, pharmaceutical and cosmetic industries (Hemming, 2013; Dolatabadi et al, 2012). It is evident that the essential oil contains a high proportion of cytotoxic, antioxidant, and anti-microbial compounds with health-beneficial effects such as antifungal effects, cancer prevention activities as well as scavenging of reactive oxygen species (ROS) activity because of its valuable constituents (Miguel, 2011; Moghaddam et al., 2013; Sun et al., 2014).

The sustainability of medicinal plants' yield is negatively affected by salt stress in many parts of the world (Abdelshafy Mohamad et al, 2020). Salinity causes a broad range of physiological changes in plants, induces impairments of metabolic processes, such as photosynthesis, pigment synthesis, secondary metabolite accumulation, destruction of the chloroplast and thylakoid systems, and inhibits the photosystem II (PSII) activity, which results in the excess accumulation of ROS (Ashraf et al., 2018; Monetti et al., 2014). In this regard, chlorophyll fluorescence is widely used to detect plant physiological status and to determine photosynthetic damage under various stresses, because the same can show the integrity of the thylakoid membrane, the quantum yield of photosystem II (PSII), and the balance between the metabolism process and energy production (Lu and Zhang, 1998; Giardi et al, 1996). It has been proved that salinity has a negative effect on the photochemical efficiency of PSII as shown by chlorophyll fluorescence parameters such as qP, ΦPSII, and Fv/Fm (Aroca et al, 2013; Radić et al., 2013). In this regard, previous research showed that salt stress reduced the quantum yield of photosystem II; whereas, it increased non-photochemical extinction in lettuce plants (Aroca et al, 2013), and decreased the ratio of the quantum yield of actual PSII photochemistry in durum wheat (Yaghoubi Khanghahi et al, 2020).

Monoterpenes play an important role in plants' response to environmental stresses. Nevertheless, there are few studies focusing on the relationship between terpene biosynthesis and the efficiency of PSII. Terpenoid biosynthesis patterns are mostly affected by photosynthetic carbon assimilation and partly regulated by

environmental stresses (Valifard et al, 2018; Haberstroh et al, 2018). There are also some reports about the anti-stress activity of monoterpenes in plants under stressful conditions. For example, it has been reported that the Monoterpenes in *Quercus ilex* can improve membrane integrity by ROS-scavenging, photoprotective, and thermotolerance roles (Peñuelas & Llusià., 2002, Loreto et al, 2002).
Applying Plant beneficial rhizospheric microorganisms (PBRMs) like symbiotic fungi is an important environmental strategy to overcome the deleterious effects of salinity stress, and to improve plants' yield and performance. Arbuscular mycorrhizal fungi and *Piriformospora indica* (also named as *Sebacinales indica*) are two of these beneficial endophytic fungi which increase plant resistance to various environmental stresses through a wide range of mechanisms; mechanisms such as stimulating the immune system of plants (Chen et al, 2017; Khalvandi et al, 2019), promoting nutrient uptake (Wu et al., 2018), and stimulating root growth (Mansotra et al., 2015). It is believed that symbiotic relationship with *P. indica* can help to ameliorate photo-oxidative damages to PSII and electron transfer chain (Yun et al, 2018), also, it increases the synthesis of secondary metabolites in various plants (Zolfaghari et al., 2013; Kari Dolatabadi et al., 2011). Several studies have shown that symbiotic relationship with *P. indica* can stimulate the human health-promoting compounds synthesis in various plants. For example: podophyllotoxins in *Linum album*, thymol and carvacrol in *Thymus vulgaris*, menthol in *Mentha piperita* (Dolatabadi et al, 2017), and estragole in *Ocimum basilicum*, pharmaceutically useful compounds (Baldi et al., 2010; Tarraf et al., 2015; Keramati et al, 2016). Moreover, it can reduce undesirable compounds for human nutrition, such as erucic acid and glucosinolates contents in *Brassica napus* (Su et al., 2017).
The present study aims to investigate the hypothesis that co-inoculation of AMF and *P. indica* can protect peppermint plants against adverse effects of the salt stress through modifying some physio-biochemical characteristics.

2. Materials and Methods

Growth Conditions:
Plants were grown in the greenhouse at Genetics and Agricultural Biotechnology Institute of Tabarestan at Sari Agricultural Sciences and Natural Resources University. A factorial experiment was carried out in a completely randomized design with three replications. Plants were treated with four levels of fungal inoculation including no-inoculation (control), *Piriformospora indica* (*P. indica*), Arbuscular mycorrhizal fungi (AMF), and co-inoculation with *P. indica* and AMF (*P. indica* + AMF) under four salinity levels including 0, 3, 6 and 9 dSm^{-1} (the Caspian Sea water and distilled water mixture was used for irrigation) (Table 1). In order to reach each level of salinity stress: determined amount of the Caspian Sea water and distilled water were mixed and then the desired salinity was determined by EC meter. *P. indica* and AMF were prepared from Sari Agricultural Sciences and Natural Resources University, Sari, Iran. 10 g of Arbuscular mycorrhizal inoculum (with a density of 120 active spores per gram) and 10 ml of *Piriformospora indica* suspension (1×10^9) were added to the pots. Peppermint rhizomes were planted in plastic pots, and were irrigated with saline water after four weeks of planting. Plant physiological measurements and sampling for the desired traits were carried out after two months of planting in all plants. The roots were bleached in a solution of KOH 10%, they were painted, using 5% solution of ink and vinegar. Then, 40 pieces of stained root were spread out in a Petri dish. The colonization percentage was measured by using the gridline intersects method optical microscope (10- 40X) (Vierheilig et al., 1998; Norris et al., 1992; Varma et al., 1999).
Physiological parameters:
Chlorophyll fluorescence parameters were measured in the last fully developed leaf by using pulse amplitude modulated fluorometer (PAM-2500, Walz, Germany). First, leaves were placed in darkness for 30 minutes using specific leaf clamps. The samples were exposed to low-intensity light [< 0.1 μmol (photon) m^{-2} s^{-1}, red light]. Then, a saturating light pulse [> 8,000 μmol (photon) m^{-2} s^{-1}, white light) was turned on for 1 s (one pulse). The minimum fluorescence (Fo) and maximum fluorescence (Fm) were determined in dark-adapted leaves. The variable fluorescence (Fv) and maximum quantum photosystem II efficiency (Fv/Fm) were evaluated based on Equations 1 and 2. and the effective photochemical quantum efficiency II [ΦPSII], electron transfer rate [ETR, μmol(electron) m^{-2} s^{-1}), non-photochemical quenching (NPQ), the quantum yield of regulated energy dissipation (YNPQ), and Quantum yield of non-regulated energy dissipation in PSII (Y (NO)) were calculated by using Equations 3 and 6 (Khanghahi et al, 2012).
1) Fv= Fm- Fo
2) Fv/Fm= (Fm- Fo)/ Fm
3) ΦPSII= (Fm′ − F)/Fm′
4) NPQ = (Fm/Fm') – 1
5) Y(NPQ) = (F/Fm') – (F/Fm)
6) Y(NO) = F/Fm
F= steady-state fluorescenc
Fm'= maximum fluorescence measured in light-exposed leaf samples.

Stomatal conductance (gs) was determined by using a porometer data. Chlorophyll a and b concentrations were calculated based on the Arnon's methods (1949).
The minerals Na^+ and K^+, P in peppermint leaves were determined according to Ntatsi et al. (2017). The amount of K^+, P and Na^+ were assayed via an atomic absorption spectrophotometer, microplate spectrophotometer, and flame photometer, respectively.
Membrane permeability was monitored based on procedures described in Lutts et al. (1996).
Essential oil extraction:
The essential oil of peppermint was extracted from dried leaves by using hydro-distillation in Clevenger's apparatus. Chemical composition of peppermint essential oil (Figure. 1) was analyzed by gas chromatography/mass spectrometry (GC/MS) (Khalvandi et al, 2019).
Statistical analysis*:*
SAS (9.2) statistical program was used for statistical analysis and a mean between treatments was compared using an LSD (Least Significant Difference) test ($P < 0.05$). Principal component analysis (PCA) and Heatmap was performed with the R language.

3. Results

Analysis of variance showed that salinity stress, P. indica, and AMF significantly affected all measured traits. According to the results, a significant two-way interaction was observed between salinity and inoculation treatment in essential oil, Na, membrane electrolyte leakage, Fv/Fm, ΦPSII, Fo, and stomatal conductance (Table 2 and 3); however, no significant Interaction was observed in other parameters. The main effects were significant for Fm, Fv, P, K, ETR, NPQ, Y(NPQ), Y(NO), chlorophyll a, and chlorophyll b (Table 2 and 3).

Colonization rate

In the microscopic investigation, not only chlamydospores and hyphae of *P. indica*, and vesicles, arbuscules, and intraradical hyphae of AMF were observed in the peppermint root cortex, but also a mycelial network of AMF and *P. indica* was spread around the roots (Figure. 2). The investigation into the AMF and *P. indica* and co-inoculation of them with plants showed 87%, 77%, and 89% colonization rate, respectively. Salt stress significantly reduced the ability of endophytic fungi to colonize with peppermint root. The decline was more noticeable when plants were inoculated with *P. indica* under medium level of salinity (6 dS $m^{-1)}$. Generally, the highest decline in the percentage of root colonization in both fungi (AMF and *P. indica*) was observed in the 9 dS m^{-1} level of salinity. The synergistic association of *P. indica* and AMF considerably increased the colonization ability of them with peppermint roots even under intense salt stress (Figure. 3).

Drought stress increases membrane electrolyte leakage (EL)

Membrane electrolyte leakage was increased under salinity stress by up to 70.4% (9 dSm^{-1}) (Figure. 4.a and Table S1), while microbial inoculation significantly alleviated oxidative damage to the membrane permeability even under severe salt stress. However, the difference between *P. indica* and AMF was not significant. The decrease in the percentage of EL was more noticeable when plants were co-inoculated by both *P. indica* and AMF. The results showed that co-inoculation was more effective than either single inoculation with *P. indica* or AMF for reducing membrane electrolyte leakage in the high level of salinity (9 dSm^{-1}).

Chlorophyll fluorescence parameters

Primary fluorescence (Fo), Maximum fluorescence (Fm), variable fluorescence (Fv)

It is known that salinity stress causes photosynthetic apparatus injury . Our data showed that salinity and microbial inoculation remarkably affected the Chlorophyll fluorescence; as salt stress led to a remarkable increase in Fo value, with maximum enhancement (29.6% and 28.9%, respectively compared to the control treatment) in plants which were grown under medium and high levels of salinity (Figure. 5a and Table S1). In addition, the increase in Fo was consistently accompanied by a decline in Fm and Fv values in salinity-treated leaves compared to the control (33.3%, and 48.81% reduction, respectively, when plants were subjected to the severe stress) (Figure. 6 and Table S2). As indicated in the figure. 5a, symbiotic relationship with *P. indica* and AMF and their co-inoculation significantly reduced Fo, while the same treatments significantly enhanced Fm and Fv. The highest Fm and Fv were observed in *P. indica* -inoculated plants (7.09%, and 24.33% higher, respectively compared to the control) (Figure. 6 and Table S3).

Maximum photochemical quantum yield of PSII (Fv/Fm) was reduced under salt stress

The Fv/Fm ratio considerably decreased when plants were subjected to salinity stress; the reduction was even greater when the higher salinity level (9 dSm^{-1}) was applied (Figure. 5a). As expected, the symbiotic relationship with endophytic fungi was associated with the increased Fv/Fm ratio under both normal and salinity conditions. This improvement was more noticeable when salinity reached 9 dS m^{-1}, as almost increased by 11.47 %, 9.83%, and 6.55%, with single and co-inoculation with *P. indic*a and AMF respectively compared to the control plants (Figure. 5a and Table S1).

Salt stress significantly reduced photochemical efficiency of photosystem II (ΦPSII) and ETR

In this experiment, ΦPSII and ETR values were sharply decreased under saline condition (Table S2), with maximum reduction (39.7% and 53.36% lower respectively, compared to the control) in plants grown under high salinity (9 dSm^{-1}) (Figure. 5 and 6). In general, endophytic fungi and especially their co-inoculation significantly

improved the ability of plants to respond to the negative effects of salinity, with the most obvious improvement of ΦPSII in 9 dSm^{-1}, (13.86% compared to control plants) (Figure. 5). ETR was also positively affected by endophytic fungi inoculation, as the highest ETR (24.11%) was observed in co-inoculated plants (Figure. 6 and Table S3)

Nonphotochemical chlorophyll quenching (NPQ), YNPQ and YNO were increased under salt stress

Salinity had significant effects on NPQ, YNPQ and YNO values in peppermint plants. The plants subjected to salt stress showed a remarkable increase in NPQ, YNPQ and YNO values in comparison to the control plants. The highest NPQ, YNPQ and YNO values (30.76%, 52.17% and 49.09%, respectively compared to controlled plants) was observed in plants which were grown under salt stress (9dsm^{-1}) (Figure. 6 and Table S2).

The results indicated that the fungi inoculation significantly reduced the NPQ, YNPQ and YNO values compared to controlled plants. The most reduction was observed in co-inoculated plants (Figure. 6 and Table S3).

Stomatal conductance (gs) is decreased under salt stress

The effect of saline condition on the stomatal conductance (gs) in the peppermint plants is shown in Figure. 5d. Increasing salinity led to a reduction in stomatal conductance. As indicated in Figure. 5d, gs reached to the lowest level under 9 dSm^{-1} of salinity (84.2% lower compared to the control). In contrast, fungi inoculation remarkably increased gs (53.87%, 47.91%, and 57% higher in *P. indica*, AMF and co-inoculation, respectively compared to un-inoculated plants grown under salt stress (9dsm^{-1})) (Figure. 5d and Table S1).

Salt stress reduces chlorophyll content

Salt stress led to a remarkable decrease in chlorophyll a and b compared to the control. The lowest contents of chlorophyll a and b, were observed in the plants treated with sever salinity (35.82% and 26.87%, respectively compared to the control plants) (Figure. 6b and Table S2). As it can be seen in Figure 6a and Table S3, fungi inoculation and especially co-inoculation significantly enhanced chlorophyll a and b contents (14.71% and 12.66%, respectively compared to controlled plants).

P, K, Na

The effect of saline condition on the minerals (P, K^+ and Na^+) concentrations in the peppermint plants is shown in Figure. 4c and 7. According to the results, Na content was dramatically increased in the peppermint leaves when plants were exposed to salt stress. Fungi inoculation ameliorated salinity negative effects, and the alleviation ratio (25.11% lower than control plant) was more considerable in co-inoculated plants under high (9 dSm^{-1}) level of salinity (Figure. 4c and Table S1). However, the opposite trend was observed for K^+ and P, as increasing in Na level was accompanied by a decline in P and K^+ content. This reduction was even greater when the higher salinity concentration (9 dSm^{-1}) was used (49.53% and 76.27% respectively, compared to the control) (Figure. 7a and 7b and Table S2). Our data shows that microbial inoculation positively affected the absorption of P and K in rhizosphere soil. The highest P content (43.58%) was observed in co-inoculated plants (Figure. 7d and Table S3).

Principal component of chlorophyll fluorescence parameters

Principal component analysis was conducted on the data of the salinity stress × 9 traits selected of leaf chlorophyll fluorescence parameters. The association between the traits were compared across the control, AMF, *P. indica* and co-inoculation treatments (Figure. 8). The score plot displayed four different groups, which were associated with four salinity samples, indicating a distinct separation of the chlorophyll fluorescence profiles under salinity conditions (Figure. 8). The PC1 mainly explained the separation of samples caused by 6 and 9 sm^{-1} of salinity (inoculation and non-inoculation), which was significantly positively correlated with YNPQ, YNO, Fo and, NPQ; whereas, PC2 mainly described non-stressed peppermint plants under low and mild salinity (inoculation and non-inoculation), it also showed the positive correlation with the Fm, Fv, Fv/Fm, ETR and, ΦPSII. Additionally, PCA findings, showed a tight positive association between Fm and Fv while they were associated negatively with Fo.

Essential oil content and composition

Salinity stress negatively affected essential oil content. Generally, salinity levels decreased essential oil content, and the lowest amount was recorded under 9 dSm^{-1}. Surprisingly, fungal symbiosis had a positive impact on essential oil content under all evaluated conditions. However, when the higher salinity concentration (9 dSm^{-1}) was used, co-inoculation was more effective in improving essential oil content compared to those of single inoculated plants (Figure. 4b and Table S1).

Essential oil composition was affected by *P. indica*, AMF, co-inoculation, salinity, and their interactions (Table 4 and Figure. 10). In all essential oil samples, the major constituents were Menthone, Menthol, 1,8-Cineole, and iso-menthone, respectively. As it can be seen in Table 4, the pattern of Menthone and Menthol increase differed in stress and non-stress conditions, indicating that microbial inoculation positively enhanced Menthone and Menthol content in both conditions. The highest Menthone content was recorded in the AMF-inoculated plant's essential oil, whereas the highest Menthol content as the second main component was observed in co-inoculated plants grown under salt stress (Table 4). A similar pattern was also observed for 1,8-Cineole, while the pattern for Menthofuran, another major component, was completely different, where salinity and Microbial inoculation reduced this content compared to the control plants (Table 4).

Piperitone content was increased under high salinity stress (9 dSm^{-1}) while β – Pinene and Terpinolene decreased compared with control samples under the same salinity level. Under both control and salinity conditions, fungi

inoculation enhanced α-Pinene, β – Pinene, 1,8-Cineole, Menthone, Menthol, neoisomenthol, Pulegone, α-Terpineol, Menthyl acetate, Carvacrol, methyl ether, β – Bourbonene, Caryophyllene, and germacrene D. In addition, higher levels of Menthone, Menthol and 1,8-Cineole were considerable in co-inoculated plant under salinity stress.

Heatmap and principal component of the essential oil constituents

As it can be seen in Figure. 9 and 10, Dim1 or PC1 accounted for 42.3% of the total variation and was mainly influenced by hydrocarbon monoterpenes (which is mainly due to the relative percentage variation of menthol), hydrocarbon sesquiterpenes, and alcohol monoterpenes. The PC1 mainly explained the separation of samples caused by endophytic fungi (salinity and non-salinity conditions), whereas, PC2 mainly explained salinity-treated and control peppermint plants (Figure. 9).

A Heatmap analysis was carried out in order to identify the significantly changed metabolites between inoculated and non-inoculated peppermint under salinity and non-salinity conditions (Figure. 11). Interestingly, salinity and non-salinity treatment (inoculated plants and non-inoculated plants) were separated from each other by two opposing groups (Figure. 11). Based on cluster analysis, two clusters of metabolites can be identified; all metabolites of the PC1, except α-Humulene, were classified in the first cluster, whereas the other group consisted of the PC2. However, linalool, Piperitone and Menthofuran were not included in either of the first two components. Controlled plants contained the higher iso-menthone, Terpinolene, and limonene together with Menthofuran and Viridiflorol which is related to oxygenated terpenes. It is possible to observe that co-inoculation treatment largely altered the relative percentage proportion of the essential oil compounds, with a high relative percentage of Menthol, Menthone, β - Pinene, 1,8-Cineole, Caryophyllene oxid, α-Terpineol, Menthyl acetate, β - Bourbonene, linalool, β – Farnesene, and sabinene. However, some metabolites showed higher percentages in control plants and co-inoculation had no significant effect on their production. The significantly changed metabolites in peppermint leaves under salinity stress were shown in Figure. 14.

4. Discussion

In the present study, the root colonization with *P. indica* and AMF were remarkably reduced by increasing salinity (6 to 9 dSm^{-1}). Similar observations were reported in other plants in which salinity inhibited the formation of the spores and arbuscules and growth of fungal hyphae (McMillen et al., 1998, Wu et al., 2010; khalvandi et al, 2019; Hadian-Deljou, et al, 2020).

It has been well documented that salinity induces excessive generation of ROS, the phenomenon that causes oxidative damage to thylakoid membrane components (disrupts the functional integrity of lipid membranes), and impairs electron transport function (Yang et al, 2021). In the current study, while salt stress decreased ETR, Fm, Fv/Fm ratio, and ΦPSII, the Fo and NPQ rates showed an increase under salinity conditions. Such alterations in fluorescence parameters can mainly be linked to the impairment of PSII function and inhibition of the electron transfer process; this may probably be due to 1: the membrane system damage in peppermint, which increases membrane electrolytic leakage (Figure. 4a), and 2: an increase in toxic ions such as Na+ (Figure. 4c).

These results provide a correlation (97%, $P < 0.05$) between the Fm and chlorophyll a. It should be mentioned that the decrease of Fm in response to salinity was along with a reduction in chlorophyll content (Figure. 6b). Fm reduction may be linked to the deactivation of chlorophyll protein compounds due to leaf chlorosis, or to alterations in any biochemical reactions (Gilmore and Björkman, 1995). Similar findings about increased Fo and decreased Fm were also reported on maize under salt stress (Sheng et al, 2008), low temperature stress (Zhu et al., 2010), and on winter wheat under drought stress (Lu and Zhang, 1998).

Increased Fo rate can indicate potential damages to PSII; damages such as D1 protein degradation, decreased thylakoid membrane integrity, damage to electron transport chain of photosystem II, reduction of plastoquinone electron receptors, lack of complete oxidation plastoquinone, and separation of light-harvesting protein complexes in chlorophyll a/b (Calatayud et al., 2006; Baker, 2008; Ashraf and Harris, 2013).

Fv/Fm represents the energy transfer efficiency from the light-harvesting complex of photosystem II to the photosystem II reaction center. Fv/Fm reduction under salinity stress can be due to limitation in the electron transport chain, which can destruct PSII reaction centers and reduce PSII maximum efficiency (Porcar-Castell et al., 2014; Giardi et al, 199, Shahabivand et al, 2017). In our experiment, the decrease in chlorophyll was accompanied by a decline in ΦPSII (Figure. 11), and Fv/Fm parameters under saline condition, which can be explained by a lower efficiency of energy transfer from antenna chlorophylls to the photosystem II reaction centers.

This result corroborates with those of previous studies reporting that salinity decreased Fv/Fm, ΦPSII and qp in durum wheat (Yaghoubi Khanghahi et al, 2020), Fv/Fm, ΦPSII, stomatal conductance and chlorophyll content in maize (Wang et al, 2020), and Chl a, Chl b, stomatal conductance and Fv/Fm in Canola (Athar et al, 2015). It has been well documented that reduction in the chlorophyll contents under salt stress reduces light reactions and electron transport in PS II from primary to secondary acceptor (Nilkens et al., 2010; Murchie and Lawson, 2013). Furthermore, we observed an inverse relationship between ΦPSII and NPQ value in peppermint ($r^2 = 0.81$; $p < 0.001$) (Figure. 12). The decrease in ΦPSII and the significant increase in NPQ in peppermint leaves under salinity stress indicates a decrease in photosynthetic process and carbon fixation capacity; this impairment decreases

utilization of electron transport products and induces greater thermal dissipation of light energy (Tavakkoli et al, 2010).

Our results showed that although the rate of colonization of *P. indica*, AMF and their co-inoculation symbiosis were negatively affected by high levels of salinity, the presence of these fungi in the roots of the plant caused a slight improvement in the function of the plant photosynthetic apparatus. According to the reports of some researchers, these changes can be due to the presence of symbiotic fungi in salinity stress conditions which can improve PSII function by increasing the transcription of chloroplast genes which are responsible for coding antenna proteins (involved in transferring stimulated energy) (Evelin et al, 2019).

The positive effects of plant-microbe interactions on PSII photochemical activity have been reported in many plants; for example, the interaction between AMF fungi and *P. indica* improved the efficiency of energy capture in PSII in pineapple plantlets (Moreira et al. 2015); *P. indica* increased photochemical efficiency of PSII, Fm, Fv/Fm and NPQ, and alleviated damages to the photosynthetic apparatus under salt stress in tomato plants (Ghorbani et al, 2018); symbiotic relationship with *P. indica* in wheat and sunflower, and *G. mosseae* in *Lolium perenne* L. protected photosystem II and enhanced the photosynthetic electron transport capacity of PSII, increased Fv/Fm and ETR values, and Chl-a and Chl-b amounts in Cd-contaminated soil (Zhang et al, 2018; Shahabivand et al, 2017; Shahabivand et al, 2012).

Endophytic fungi can reduce the toxic impact of salinity on the chloroplast and chlorophyll by stimulating antioxidant capacity and secondary metabolism related to ROS scavenger activity (such as PPO, DPPH-scavenging activity), by synthesizing phenolic compounds in peppermint, and last but not least, by alleviating damages to the cell membranes caused by salt stress (Zai et al, 2012; Khalvandi et al, 2019; Sun et al, 2012). Yun et al. (2018) observed a definite relationship between quantum efficiency of photosystem II, electron transport chain, and salt tolerance with the activity of antioxidant enzymes and plant hormone levels in *Zea mays* L. plants under salinity stress (Yun et al, 2018).

It has been well documented that there is a close link between reduction of the quantum efficiency of PSII and photoinhibition and depression of photosynthesis under environmental stress. The above results suggest that a low light harvesting efficiency due to reduced chlorophyll content is the first step of photosynthesis inhibition in response to environmental stresses. In this study, a direct correlation between an increasing chlorophyll content and a high ΦPSII in microbial colonization treatments at the presence of salinity can be attributed to stimulating the accumulation of chlorophyll by the microbes which ameliorated the negative effects of salinity on light-harvesting and electron transfer to plastoquinone.

The results of this study showed that severe salinity stress remarkably reduced P and K^+ uptake in non-inoculated plant because of the high rate of Na^+ absorption. Moreover, the inhibitory effect of ion toxicity, such as Na^+ and Cl^-, on the mineral absorption directly influences photosynthetic performance. Several reports suggest a direct correlation between nutrient imbalance and the simultaneous decline in PSII function in salt-stressed plants (Loudari et al, 2020; Qu et al, 2012); it may be due to reducing membrane permeability and increasing leakage of ions (Figure. 4a), and antagonistic relationship between excess uptake of Na^+ and mineral nutrient such as P and K^+ under severe salinity stress (Figure. 12) leading to an imbalance in cellular ion homeostasis (Yang et al, 2021). Nevertheless, in inoculated plant endophytic fungal symbiosis increased P and K+ content (Figure. 6a) and decreased Na+ uptake in peppermint leaves (Figure. 4c). It has been well established that endophytic fungi can keep the balance of sodium to potassium ratio through inducing the expression of genes which are involved in sodium excretion and potassium entry into the roots. Also, vesicles and arbuscules play a key role in nutrient transfer, and particularly in mobilization of phosphorus (Kumar, 2020). Endophytic fungi can release P from sources which are not accessible for plant roots and transforms P to the available forms, which are transported to host plants through fungal hyphae directly or taken up by roots (Wu et al. 2018). This can be attributed to the ability of endophytic fungi to increase the activity of phosphate transporters and also to stimulate phosphatase activities in the plant as well as in the rhizosphere soil (Wu et al, 2018).

Increased mineral nutrients absorption due to plant-microbe interactions influences photosynthesis performance by regulating gas exchange and photosynthesis pigments. There is a close relationship between the chlorophyll and gas interchange with PSII efficiency (Figure. 12). Positive effects of fungal symbiosis on the capacity of gas exchange are mainly through elevating the mineral absorption and improving water status. In agreement with the present results, Xu et al (2016) reported that the positive effect of AMF on Fv/Fm increasing in maize seedlings can be attributed to the absorption of mineral nutrients, activating mediated genes and sink stimulation. AMF inoculation also positively influenced chlorophyll biosynthesis in *Ligustrum vicaryi* by alleviating the detrimental effects of salinity on Mg^{2+} absorption and therefore improved Fv/Fm values (Qiu et al, 2020).

Furthermore, monoterpene emissions protect the photosynthetic apparatus and maintain PSII stability under stressful conditions through inducing photoprotective mechanisms. Besides, it maintains PSII stability through partitioning of the excess electrons into other sinks in the terpenoid biosynthetic pathway (Bertamini et al, 2020). Based on the results, there was a close correlation between the ETR and some essential oil components (Menthol, Menthone, and 1,8-cineole) (Figure. 13). Similar observations were reported in other plants. For example: it has been shown a tight link between monoterpene emission, increasing Fv/Fm ratio, and ETR in the *Vitis vinifera*

under heat stress (Bertamini et al, 2020); a positive correlation between the secondary metabolites and the quantum efficiency of PII in *Lactuca sativa* was reported (Ouzounis et al, 2015); also, a positive tight link between ETR and UPSII with monoterpene and sesquiterpene emissions in Q. coccifera and R. officinalis, respectively (Ormeño et al, 2009); photosynthetic electron transport rate and limonene emission in *Artemisia annua* (Rapparini et al, 2008); and finally, monoterpene emissions in Quercus species (Niinemets et al, 2002). Besides, the quantum yield of PSII (ΔF/Fm) in *Quercus ilex* L. was higher when plants were exposed to selected exogenous monoterpenes (Loreto et al 1998). According to the results, higher ETR values besides a higher monoterpene content in mycorrhizal plants can be attributed to a better physiological status, changing in the pattern of carbon partitioning, and accumulation and biosynthesis of terpenes. The possible mechanism is that endophytic fungus exerts anti-stress effects by inducing the expression of antioxidant biosynthetic genes (antioxidant potential) and activating antioxidant enzymes. The mechanism which alleviates the damages to chloroplast and chlorophyll, helps to maintain the relative stability of the organelle, and improves performance of ROS scavengers, such as CAT, PPO, and SOD activities.

It is believed that phosphate bonds can be a limiting factor in terpenoid biosynthesis. Naturally, monoterpenes emission is controlled by the availability of photosynthetic substrates. The biosynthesis of terpenoids in endophytic fungi treated plants can mainly be linked to their role in mineral nutrients uptake, especially phosphorus supply for NADPH and ATP (substrates of terpenoids biosynthesis), and increasing peltate glandular trichomes (Zolfaghari et al, 2013; Rai et al,2001; Sirrenberg et al,2007; Song, 2005; Loomis, and Corteau, 1972). Several reports have suggested a direct correlation between the synthesis of menthol, menthone, 1,8-cineole, citral, linalool and caryophyllene with antimicrobial and fungicide properties. The possible mechanism can be explained by increasing biosynthesis of these compounds in inoculated plants through activating defensive reactions (Coban, et al., 2016; Khalvandi et al, 2019). It has also been shown that reduction in CO_2 assimilation capacity under stress condition and defense response to microbial inoculation probably redirected resources to the mevalonic acid (MVA) (Gupta et al, 2018; Vickers et al, 2009).

5. Conclusion

Co-inoculation of mycorrhiza and *Piriformospora indica* fungi had a synergic effect on the enhancement of peppermint essential oil and its physiological characteristics. Fungal co-inoculation protected peppermint plants under salinity conditions. There are many reports about the synergistic effect of symbiotic fungi on the improvement of plant function under various environmental conditions; however, there are other research which did not support the synergistic effect and emphasized that separate application of endophyte fungi had more significant effect on the antioxidant activity and Fv / fm. (Shahabivand et al., 2012; Moreira et al., 2016; Ray and Walsalakumar, 2010). Thus, it needs further research in the future because applying microbial treatments for boosting plant secondary metabolite production could be a natural way as well as a sustainable approach in the promising herbal medicine industry.

Reference

Abdelshafy Mohamad, O. A., Ma, J. B., Liu, Y. H., Zhang, D., Hua, S., Bhute, S., ... & Li, L. (2020). Beneficial endophytic bacterial populations associated with medicinal plant Thymus vulgaris alleviate salt stress and confer resistance to Fusarium oxysporum. Frontiers in plant science, 11, 47.

Arnon, D. I. (1949). Copper enzymes in isolated chloroplasts. Polyphenol oxidase in Beta vulgaris. Plant physiology, 24(1), 1.

Aroca, R., Ruiz-Lozano, J.M., Zamarreño, Á.M., Paz, J.A., García-Mina, J.M., Pozo, M.J., López-Ráez, J.A., (2013). Arbuscular mycorrhizal symbiosis influences strigolactone production under salinity and alleviates salt stress in lettuce plants. J. Plant Physiol.

170, 47–55.

Ashraf, M., Harris, P.J.C., (2013). Photosynthesis under stressful environments: an overview. Photosynthetica 51, 163e190.

Ashraf, M., Shahzad, S.M., Imtiaz, M., Rizwan, M.S., (2018). Salinity effects on nitrogen metabolism in plants–focusing on the activities of nitrogen metabolizing enzymes: A review. J. Plant Nutr. 41, 1065–1081.

Athar, H. U. R., Zafar, Z. U., & Ashraf, M. (2015). Glycinebetaine improved photosynthesis in canola under salt stress: evaluation of chlorophyll fluorescence parameters as potential indicators. Journal of agronomy and crop science, 201(6), 428-442.

Baker, N.R., (2008). Chlorophyll fluorescence: a probe of photosynthesis in vivo. Annu. Rev. Plant Biol. 59, 89e113.

Baldi, A., Farkya, S., Jain, A., Gupta, N., Mehra, R., Datta, V., Srivastava, A.K., & Bisaria, V. S. (2010). Enhanced production of podophyllotoxins by co-culture of transformed Linum album cells with plant growth-promoting fungi. Pure and Applied Chemistry, 82(1), 227-241.

Bertamini, M., Faralli, M., Varotto, C., Grando, M. S., & Cappellin, L. (2020). Leaf Monoterpene Emission Limits Photosynthetic Downregulation Under Heat Stress in Field-Grown Grapevine.

Calatayud, A., Roca, D., Martínez, P.F., (2006). Spatial-temporal variations in rose leaves under water stress conditions studied by chlorophyll fluorescence imaging. Plant Physiol. Biochem. 44, 564e573.

Chen, J., Zhang, H., Zhang, X., & Tang, M. (2017). Arbuscular mycorrhizal symbiosis alleviates salt stress in black locust through improved photosynthesis, water status, and K+/Na+ homeostasis. Frontiers in plant science, 8, 1739.

Coban, O., Göktürk Baydar, N., (2016). Brassinosteroid effects on some physical and biochemical properties and secondary metabolite accumulation in peppermint (*Mentha piperita* L.) under salt stress. Ind Crops Prod. 86, 251-258.

Dolatabadi, H. K., Goltapeh, E. M., Moieni, A., & Varma, A. (2012). Evaluation of different densities of auxin and endophytic fungi (Piriformospora indica and Sebacina vermifera) on Mentha piperita and Thymus vulgaris growth. African Journal of Biotechnology, 11(7), 1644-1650.

Dolatabad, H. K., Goltapeh, E. M., Safari, M., & Golafaie, T. P. (2017). Potential effect of Piriformospora indica on plant growth and essential oil yield in Mentha piperita. Plant Pathol. Quarantine, 7, 96-104.

Evelin, H., Devi, T. S., Gupta, S., & Kapoor, R. (2019). Mitigation of salinity stress in plants by arbuscular mycorrhizal symbiosis: current understanding and new challenges. Frontiers in Plant Science, 10, 470.

Giardi, M. T., Cona, A., Geiken, B., Kučera, T., Masojidek, J., & Mattoo, A. K. (1996). Long-term drought stress induces structural and functional reorganization of photosystem II. Planta, 199(1), 118-125.

Gilmore AM, Björkman O. (1995). Temperature sensitive coupling and uncoupling of ATPase-mediated, nonradiative energy dissipation: Similarities between chloroplasts and leaves. Planta. 197:646-654.

Ghorbani, A., Razavi, S. M., Ghasemi Omran, V. O., & Pirdashti, H. (2018). Piriformospora indica inoculation alleviates the adverse effect of NaCl stress on growth, gas exchange and chlorophyll fluorescence in tomato (Solanum lycopersicum L.). Plant Biology, 20(4), 729-736.

Gupta, R., Singh, A., Srivastava, M., Singh, V., Gupta, M. M., & Pandey, R. (2017). Microbial modulation of bacoside A biosynthetic pathway and systemic defense mechanism in Bacopa monnieri under Meloidogyne incognita stress. *Scientific reports*, *7*(1), 1-11.

Hadian-Deljou, M., Esna-Ashari, M., & Mirzaie-asl, A. (2020). Alleviation of salt stress and expression of stress-responsive gene through the symbiosis of arbuscular mycorrhizal fungi with sour orange seedlings. *Scientia Horticulturae*, *268*, 109373.

Hasan, M., Ma, F., Prodhan, Z., Li, F., Shen, H., Chen, Y., & Wang, X. (2018). Molecular and physio-biochemical characterization of cotton species for assessing drought stress tolerance. International journal of molecular sciences, 19(9), 2636.

Haberstroh, S., Kreuzwieser, J., Lobo-do-Vale, R., Caldeira, M. C., Dubbert, M., & Werner, C. (2018). Terpenoid emissions of two Mediterranean woody species in response to drought stress. Frontiers in plant science, 9, 1071.

Hemming, D., 2013. Plant sciences reviews 2012. CABI. 280 pp. (online ISSN 1749-8848).

Kari Dolatabadi, H., Mohammadi Goltapeh, E., Jaimand, K., Rohani, N., Varma, A., (2011). Effects of *Piriformospora indica* and *Sebacina vermifera* on growth and yield of essential oil in fennel (*Foeniculum vulgare*) under greenhouse conditions. J. Basic Microbiol. 33–39.

Keramati, S., Pirdashti, H., Babaeizad, V., & Dehestani, A. (2016). Essential oil composition of sweet basil (*Ocimum basilicum* L.) in symbiotic relationship with Piriformospora indica and paclobutrazol application under salt stress. *Acta Biologica Hungarica*, *67*(4), 412-423.

Khalvandi, M. Amerian, M.R., Pirdashti, H., Keramati, S., Hosseini, J. (2019). Essential oil of peppermint in symbiotic relationship with *Piriformospora indica* and methyl jasmonate application under saline condition. Industrial crops and products 127, 195-202.

Khatri, K., & Rathore, M. S. (2019). Photosystem photochemistry, prompt and delayed fluorescence, photosynthetic responses and electron flow in tobacco under drought and salt stress. Photosynthetica, 57(1), 61-74.

Kromdijk, J., Głowacka, K., Leonelli, L., Gabilly, S.T., Iwai, M., Niyogi, K.K., Long, S.P. (2016). Improving photosynthesis and crop productivity by accelerating recovery from photoprotection. Science 354, 857–861.

Kumar, G. (2020). Unit 7 Topic: Arbuscular Mycorrhizal Colonization In Plant Roots.

Li, D., Liu, H., Qiao, Y., Wang, Y., Cai, Z., Dong, B., Shi, C., Liu, Y., Li, X., and Liu, M. (2013). Effects of elevated CO2 on the growth, seed yield, and water use efficiency of soybean (*Glycine max* (L.) Merr.) under drought stress. Agricultural Water Management, 129, 105-112.

Li, Y., Song, H., Zhou, L., Xu, Z., & Zhou, G. (2019). Tracking chlorophyll fluorescence as an indicator of drought and rewatering across the entire leaf lifespan in a maize field. Agricultural Water Management, 211, 190-201.

Loomis, W.D., Corteau, R., 1972. Essential oil biosynthesis. Recent Adv Phytochem. 6, 147-185.

Loreto, F., Förster, A., Dürr, M., Csiky, O., & Seufert, G. (2002). On the monoterpene emission under heat stress and on the increased thermotolerance of leaves of Quercus ilex L. fumigated with selected monoterpenes. Plant, Cell & Environment, 21(1), 101-107.

Loudari, A., Benadis, C., Naciri, R., Soulaimani, A., Zeroual, Y., Gharous, M. E., Kalaji, H M., & Oukarroum, A. (2020). Salt stress affects mineral nutrition in shoots and roots and chlorophyll a fluorescence of tomato plants grown in hydroponic culture. Journal of Plant Interactions, 15(1), 398-405.

Lu, C., & Zhang, J. (1998). Effects of water stress on photosynthesis, chlorophyll fluorescence and photoinhibition in wheat plants. Functional Plant Biology, 25(8), 883-892.

Lutts, S., Kinet, J. M., & Bouharmont, J. (1996). NaCl-induced senescence in leaves of rice (Oryza sativa L.) cultivars differing in salinity resistance. Annals of botany, 78(3), 389-398.

Mansotra, P., Sharma, P., & Sharma, S. (2015). Bio augmentation of *Mesorhizobium cicer*, *Pseudomonas spp*. and *Piriformospora indica* for sustainable chickpea production. Physiology and molecular biology of plants, 21(3), 385-393.

McMillen, B.G., Juniper, S., Abbott, L.K., (1998). Inhibition of hyphal growth of a Vesicular arbuscular mycorrhizal fungus in soil containing sodium chloride limits the spread of infection from spores. Soil Biol. Biochem. 30, 1639–1646.

Miguel, M.G., (2011). Anthocyanins: Antioxidant and/or anti -inflammatory activities. J Appl Pharm Sci. 6, 7-15.

Moghaddama, M., Pourbaige, M., Kourosh Tabar, H., Farhadid, N., Ahmadi Hosseinie, S.M., 2013. Composition and Antifungal Activity of Peppermint (*Mentha piperita*) Essential Oil from Iran. J. Essent. Oil-Bear. Plants. 16, 506-512.

Monetti, E., Kadono, T., Tran, D., Azzarello, E., Arbelet-Bonnin, D., Biligui, B., Briand, J., Kawano, T., Mancuso, S., Bouteau, F., (2014). Deciphering early events involved in hyperosmotic stress-induced programmed cell death in tobacco BY-2 cells. J. Exp.

Bot. 65, 1361–1375.

Moreira, B. C., Junior, P. P., Jordao, T. C., da Silva, M. D. C. S., Stürmer, S. L., Salomão, L. C. C., ... & Kasuya, M. C. M. (2016). Effect of inoculation of symbiotic fungi on the growth and antioxidant enzymes' activities in the presence of Fusarium subglutinans f. sp. ananas in pineapple plantlets. Acta Physiologiae Plantarum, 38(10), 1-14.

Murchie, E.H., Lawson, T. (2013). Chlorophyll fluorescence analysis: a guide to good practice and understanding some new applications. J. Exp. Bot. 64, 3983–3998.

Niinemets, Ü., Hauff, K., Bertin, N., Tenhunen, J. D., Steinbrecher, R., & Seufert, G. (2002). Monoterpene emissions in relation to foliar photosynthetic and structural variables in Mediterranean evergreen Quercus species. New Phytologist, 153(2), 243-256.

Nilkens, M., Kress, E., Lambrev, P., Miloslavina, Y., Müller, M., Holzwarth, A.R., Jahns, P. (2010). Identification of a slowly inducible zeaxanthin-dependent component of nonphotochemical quenching of chlorophyll fluorescence generated under steady-state conditions in Arabidopsis.

Norris, I.R., Read, D.J., Varma, A.K., 1992. Methods in Microbiology. Techniques for Study of Mycorrhiza. Academic Press, London. 450 pp.

Ntatsi, G., Aliferis, K.A., Rouphael, Y., Napolitano, F., Makris, K., Kalala, G., Katopodis, G., Savvas, D., 2017. Salinity source alters mineral composition and metabolism of *Cichorium spinosum*. Environ. Exp. Bot. 141, 113–123.

Ormeño, E., Olivier, R., Mévy, J. P., Baldy, V., & Fernandez, C. (2009). Compost may affect volatile and semi-volatile plant emissions through nitrogen supply and chlorophyll fluorescence. Chemosphere, 77(1), 94-104.

Ouzounis, T., Razi Parjikolaei, B., Fretté, X., Rosenqvist, E., & Ottosen, C. O. (2015). Predawn and high intensity application of supplemental blue light decreases the quantum yield of PSII and enhances the amount of phenolic acids, flavonoids, and pigments in Lactuca sativa. Frontiers in plant science, 6, 19.

Peñuelas, J., & Llusià, J. (2002). Linking photorespiration, monoterpenes and thermotolerance in Quercus. New Phytologist, 155(2), 227-237.

Porcar-Castell, A., Tyystj arvi, E., Atherton, J., Van Der Tol, C., Flexas, J., Pfündel, E.E., € Moreno, J., Frankenberg, C., Berry, J.A. (2014). Linking chlorophyll a fluorescence to photosynthesis for remote sensing applications: mechanisms and challenges. J. Exp. Bot. 65, 4065e4095.

Qiu, Y. J., Zhang, N. L., Zhang, L. L., Zhang, X. L., Wu, A. P., Huang, J. Y & Wang, Y. H. (2020). Mediation of arbuscular mycorrhizal fungi on growth and biochemical parameters of Ligustrum vicaryi in response to salinity. Physiological and Molecular Plant Pathology, 112, 101522.

Qu, C., Liu, C., Gong, X., Li, C., Hong, M., Wang, L., & Hong, F. (2012). Impairment of maize seedling photosynthesis caused by a combination of potassium deficiency and salt stress. Environmental and Experimental Botany, 75, 134-141

Radić, S., Peharec Štefanić, P., Lepeduš, H., Roje, V., Pevalek-Kozlina, B., (2013). Salt tolerance of Centaurea ragusina L. is associated with efficient osmotic adjustment and increased antioxidative capacity. Environ. Exp. Bot. 87, 39–48.

Rai, M., Acharya, D., Singh, A., Varma, A., (2001). Positive growth responses of the medicinal plants Spilanthes calva and Withania somnifera to inoculation by Piriformospora indica in a field trial. Mycorrhiza. 11, 123-128.

Rapparini, F., Llusià, J., & Peñuelas, J. (2008). Effect of arbuscular mycorrhizal (AM) colonization on terpene emission and content of Artemisia annua L. Plant Biology, 10(1), 108-122.

Ray, J. G., & Valsalakumar, N. (2010). Arbuscular mycorrhizal fungi and Piriformospora indica individually and in combination with Rhizobium on green gram. Journal of plant nutrition, 33(2), 285-298.

Shahabivand, S., Parvaneh, A., & Aliloo, A. A. (2017). Root endophytic fungus Piriformospora indica affected growth, cadmium partitioning and chlorophyll fluorescence of sunflower under cadmium toxicity. Ecotoxicology and environmental safety, 145, 496-502.

Sheng, M., Tang, M., Chen, H., Yang, B., Zhang, F., & Huang, Y. (2008). Influence of arbuscular mycorrhizae on photosynthesis and water status of maize plants under salt stress. Mycorrhiza, 18(6-7), 287-296.

Shahabivand, S., Maivan, H. Z., Goltapeh, E. M., Sharifi, M., & Aliloo, A. A. (2012). The effects of root endophyte and arbuscular mycorrhizal fungi on growth and cadmium accumulation in wheat under cadmium toxicity. Plant Physiology and Biochemistry, 60, 53-58.

Sirrenberg, A., Gobel, C., Grond, S., Czempinski, N., Ratzinger, A., Karlovsky, P., Santos, P., Feussner I., Pawlowski, K., (2007). *Piriformospora indica* affects plant growth by auxin production. Physiol. Plant. 131, 581-589

Su, Z. Z., Wang, T., Shrivastava, N., Chen, Y. Y., Liu, X., Sun, C., Yin, Y., Gao, Q.K. & Lou, B. G. (2017). *Piriformospora indica* promotes growth, seed yield and quality of *Brassica napus* L. Microbiological research, 199, 29-39.

Sun, C., Johnson, J. M., Cai, D., Sherameti, I., Oelmüller, R., & Lou, B. (2010). *Piriformospora indica* confers drought tolerance in Chinese cabbage leaves by stimulating antioxidant enzymes, the expression of drought-related genes and the plastid-localized CAS protein. *Journal of plant physiology*, *167*(12), 1009-1017.

Sun, Z., Wang, H., Wang, J., Zhou, L., Yang, P., (2014). Chemical composition and anti-inflammatory, cytotoxic and antioxidant activities of essential oil from Leaves of *Mentha piperita* grown in china. PLoS One. 12, e114767.

Tarraf, W., Ruta, C., Cillis, F.D., Tagarelli, A., Tedone, L., Mastro, G.D., (2015). Effects of mycorrhiza on growth and essential oil production in selected aromatic plants. J.

Tavakkoli, E., Rengasamy, P., & McDonald, G. K. (2010). High concentrations of Na+ and Cl–ions in soil solution have simultaneous detrimental effects on growth of faba bean under salinity stress. Journal of Experimental Botany, 61(15), 4449-4459.

Valifard, M., Mohsenzadeh, S., Kholdebarin, B., Rowshan, V., Niazi, A., & Moghadam, A. (2019). Effect of salt stress on terpenoid biosynthesis in Salvia mirzayanii: From gene to metabolite. The Journal of Horticultural Science and Biotechnology, 94(3), 389-399.

Varma, A., Verma, S., Sudha Sahay, N., Bu¨tehorn, B., Franken, P., 1999. Piriformospora indica, a Cultivable Plant-Growth- Promoting Root Endophyte. Appl. Environ. Microbiol. 65, 2741–2744.

Vickers, C. E., Gershenzon, J., Lerdau, M. T., & Loreto, F. (2009). A unified mechanism of action for volatile isoprenoids in plant abiotic stress. *Nature chemical biology*, *5*(5), 283.

Vierheilig, H., Coughlan, A.P., Wyss, U., Piche, Y., 1998. Ink and vinegar, a simple staining technique for arbuscular-mycorrhizal fungi. Appl. Environ. Microbiol. 12, 5004-5007.

Tsai, M.L., Wu, C.T., Lin, T.F., Lin, W.C., Huang, Y.C., Yang, C.H., (2013). Chemical composition and biological properties of essential oils of two mint species. Trop. J. Pharm. Res. 12, 577–582.

Wang, H., Liang, L., Liu, B., Huang, D., Liu, S., Liu, R., ... & Chen, Y. (2020). Arbuscular Mycorrhizas Regulate Photosynthetic Capacity and Antioxidant Defense Systems to Mediate Salt Tolerance in Maize. Plants, 9(11), 1430.

Wu, Q.S., Zou, Y.N., He, X.H., (2010). Contributions of arbuscular mycorrhizal fungi to growth, photosynthesis, root morphology and ionic balance of citrus seedlings under salt stress. Acta Physiol. Plant. 32, 297–304. https://doi.org/10.1007/s11738-009-0407-z.

Wu, M., Wei, Q., Xu, L., Li, H., Oelmüller, R., & Zhang, W. (2018). *Piriformospora indica* enhances phosphorus absorption by stimulating acid phosphatase activities and organic acid accumulation in *Brassica napus*. *Plant and Soil*, *432*(1-2), 333-344.

Xu, H., Lu, Y., & Zhu, X. (2016). Effects of arbuscular mycorrhiza on osmotic adjustment and photosynthetic physiology of maize seedlings in black soils region of northeast China. Brazilian Archives of Biology and Technology, 59.

Yaghoubi Khanghahi, M., Strafella, S., & Crecchio, C. (2020). Changes in photo-protective energy dissipation of photosystem II in response to beneficial bacteria consortium in durum wheat under drought and salinity stresses. Applied Sciences, 10(15), 5031.

Yang, M., Sun, X., Zhu, J., Liu, Y., Chen, N., & Zhao, G. Physiological and proteomics insights into salt tolerance of two Jerusalem artichoke cultivars. Journal of Plant Biochemistry and Biotechnology, 1-10.

Yun, P., Xu, L., Wang, S.S., Shabala, L., Shabala, S., Zhang, W.Y. (2018). *Piriformospora indica* improves salinity stress tolerance in *Zea mays* L. plants by regulating Na+ and K+ loading in root and allocating K+ in shoot. Plant Growth Regul. 1–9. https://doi.org/10.1007/s10725-018-0431-3.

Zai, X. M., Zhu, S. N., Qin, P., Wang, X. Y., Che, L., & Luo, F. X. (2012). Effect of Glomus mosseae on chlorophyll content, chlorophyll fluorescence parameters, and chloroplast ultrastructure of beach plum (*Prunus maritima*) under NaCl stress. *Photosynthetica*, *50*(3), 323-328.

Zolfaghari, M., Nazeri, V., Sefidkon, F., Rejali, F., (2013). Effects of arbuscular mycorrhizal fungi on plant growth and essential oil content and composition of *Ocimum basilicum* L. J Plant Physiol. 3, 643-650.

Zhu, X. C., Song, F. B., & Xu, H. W. (2010). Arbuscular mycorrhizae improve low temperature stress in maize via alterations in host water status and photosynthesis. Plant and Soil, 331(1-2), 129-137.

Table 1. Chemical analysis of Caspian Sea water

parameter	unit	amount
EC	dS m^{-1}	15
pH	-	8.2
sodium	ppm	376.48
potassium	ppm	77.11
calcium	ppm	17
magnesium	ppm	54.5

(Khalvandi et al, 2019)

Table 2. Variance analysis of salinity and *P. indica* and AMF effects on measured chlorophyll fluorescence parameters of peppermint

S.O.V	df	fo	YII	Fv/fm	fm	fv	Y(NPQ)	Y(NO)	NPQ	ETR
Replication	2	0.0017	0.00337	0.00116	0.7396	0.7337	0.0004	0.0011	0.0045	14.7708
Salinity (S)	3	0.6930**	0.14945**	0.09022**	13.4999**	20.2384**	0.0446**	0.0456**	0.3145**	300.055**
Fungi (F)	3	0.0412**	0.01461**	0.00362**	0.4269**	0.6231**	0.0054**	0.0031**	0.0153**	41.50**
S × F Interaction	9	0.0047**	0.00097**	0.00049**	0.1005ns	0.0988ns	0.0015 ns	0.0005ns	0.0042ns	3.7037 ns
Error	30	0.0015	0.00034	0.00013	0.0772	0.0736	0.00072	0.00026	0.0027	3.304
CV%		3.04	3.26	1.52	4.83	6.11	15.44	8.3	5.42	3.304

ns = no significant, * significant (P < 0.05), ** significant (P < 0.01)

Table 3. Variance analysis of salinity and *P. indica* and AMF effects on measured physiological and biochemical parameters of peppermint

S.O.V	df	Chlorophyll a (µg/ml)	Chlorophyll b (µg/ml)	P (meq/gdw^{-1})	K^+ (meq/gdw^{-1})	Na^+ (meq/gdw^{-1})	Essential oil (%)	Stomatal conductance (mmol (H_2O) m^{-2} s^{-1})	Membrane electrolyte leakage %	colonization rate (%)
Replication	2	0.3765	0.50701	0.00255	0.02985	0.0043	0.00316	0.0593	2.9203**	11.583
Salinity (S)	3	13.9547**	5.57805**	0.12515**	3.99761**	2.1872**	6.81303**	655.0106**	4835.1701**	2267.629**
Fungi (F)	3	1.7688**	1.00595**	0.07111**	0.15185**	0.0690**	0.82588**	28.2901**	133.2135**	424.750**
S × F Interaction	9	0.0399^{ns}	0.07208^{ns}	0.00281^{ns}	0.02509^{ns}	0.0145*	0.04237**	0.4364*	13.5865**	13.824*
Error	30	0.08101	0.1196	0.00227	0.02054	0.0061	0.0068	0.1701	6.0838	5.2500
CV%		5.03	6.95	14.21	13.2	14.12	4.59	3.34	6.44	3.25

ns = no significant, * significant (P < 0.05), ** significant (P < 0.01)

Table 4. Effect of microbial inoculation on *Mentha piperita* essential oils composition under salinity stress.

No	Compound	Concentration (%)							
		Salinity* P. indica *AMF	Salinity *AMF	Salinity * P. indica	Salinit y	P. indica *AMF	P. indica	AMF	control
1	limonene	2.4	1.93	1.42	3.5	2.63	2.89	2.73	5.36
2	Carvacrol, methyl ether	1.38	1.77	2.08	1.23	2.01	2.21	1.9	1.38
3	Menthyl acetate	1.21	1.13	1.22	1.07	1.12	1.16	1.15	1.08
4	α-Pinene	1.04	1.07	1.09	1.03	1.05	0.93	1.02	0.85
5	sabinene	0.92	0.88	0.75	0.72	0.82	0.84	0.82	0.86
6	β - Pinene	0.92	0.51	0.61	0.43	0.61	0.6	0.63	0.52
7	β - Bourbonene	0.72	0.65	0.78	0.41	0.43	0.38	0.54	0.32
8	Terpinolene	0.43	0.14	0.35	0.31	0.49	0.48	0.53	0.81
9	α- Terpinene	0.28	0.27	0.23	0.23	0.41	0.48	0.42	0.64
10	cis-Ocimene	0.14	0.29	0.6	0.33	0.52	0.62	0.54	0.64
11	Piperitone	0.1	0.31	0.13	0.29	0.23	0.13	0.21	0.28
12	Menthol	19.87	19.37	18.03	15.39	18.12	18.89	17.3	14.86
13	neoisomenthol	5.41	5.27	6.18	4.46	5.37	6.09	5.35	5.07
14	Trans sabinene hydrate	0.92	0.61	0.98	0.68	0.87	0.81	0.8	0.85
15	1,8-Cineole	10.66	9.62	9.23	9.01	9.14	9.04	9.12	8.87
16	Menthofuran	5.22	6.18	5.07	5.87	5.63	5.61	5.48	7.33
17	α-Terpineol	0.87	0.52	0.85	0.58	0.75	0.63	0.72	0.61
18	Linalool	0.68	0.54	0.26	0.56	0.5	0.49	0.47	0.52
19	Menthone	34.52	31.27	32.98	31.04	34.75	34.95	35.2	29.41
20	iso-menthone	4.9	5.74	4.57	6.15	6.12	5.95	5.97	8.34
21	Pulegone	0.23	0.33	0.34	0.24	0.27	0.3	0.26	0.15
22	Caryophyllene	0.81	0.36	0.76	0.45	0.75	0.73	0.75	0.68
23	β - Farnesene	0.37	0.33	0.35	0.32	0.25	0.28	0.27	0.31
24	germacrene D	0.17	0.21	0.21	0.17	0.2	0.25	0.22	0.15
25	Caryophyllene oxid	0.19	0.13	0.14	0.11	0.11	0.12	0.1	0.06
26	α-Humulene	0.15	0.11	0.14	0.18	0.15	0.27	0.1	0.16
27	Viridiflorol	0.15	0.13	0.12	0.11	0.18	0.31	0.21	0.43

Salinity (9dsm^{-1}); *P. indica*; AMF – Mycorrhizae treatment; P. indica *AMF – *P. indica* and Mycorrhizae treatment.

Fig. 1. *Mentha piperita* essential oil chromatogram

Fig. 2. *Piriformospora indica* interacellular chlamydospores in cortex (a), *P. indica* spore and hyphae (around the roots) (b) and interadical hyphae, exteradical hyphae, vesicles and arbuscules formation of AMF (c and d) were formed in the *Mentha piperita* roots. The root-pieces were examined under light microscope at the magnification of 10–40 X.

Fig. 3. Effect of salinity on root colonization (*P. indica*, AMF and co-inoculation) in peppermint. In each figure, means with the same letter are significantly different according to LSD test at $P < 0.05$.

Fig. 4. Effect of salinity on Membrane electrolyte leakage (a), Essence (b) and Na (c) in inoculated and non-inoculated peppermint with (*P. indica* AMF and co-inoculation)

Fig. 5. Effect of salinity on Fo (a), ΦPSII (b), Fv/Fm (c) and Stomatal conductance (d) in inoculated and non-inoculated peppermint with (*P. indica*, AMF and co-inoculation)

Fig 6. Changes in various physiological traits in inoculated and non-inoculated peppermint with (*P. indica*, AMF and co-inoculation) (a), and caused by salinity stress in peppermint plant (b) *P. indica*; AMF – Mycorrhizae treatment; AMF* *P. indica* –Mycorrhizae and *P. indica* treatment.

Fig 7. Box Plots of P and K in peppermint under salinity stress (a) and in inoculated and non-inoculated peppermint with (*P. indica*, AMF and co-inoculation) (b). *P. indica*; AMF – Mycorrhizae treatment; AMF* *P. indica* –Mycorrhizae and *P. indica* treatment.

Fig. 8. Principal component analysis (PCA) (a) and 2D score plot (b) of chlorophyll fluorescence parameters in peppermint leaves. The score plot for the four-salinity levels; 0, 3, 6 and 9 dsm^{-1} were indicated in blue, orange, green and red, respectively. Abbreviations: *P. indica*; AMF – Mycorrhizae treatment; *P. indica**AMF – *P. indica* and Mycorrhizae treatment.

Fig. 9. Principal component analysis (PCA) of metabolites in peppermint leaves. AMF – Mycorrhizae treatment.

Fig 10. Changes in terpenoid content in inoculated and non-inoculated peppermint with (*P. indica*, AMF and co-inoculation) under salinity stress in peppermint plant. S – Salinity; *P. indica*; AMF – Mycorrhizae treatment; AMF* P. indica –Mycorrhizae and *P. indica* treatment.

Fig. 11. Heatmap pattern of the essential oil constituents from peppermint. AMF – Mycorrhizae treatment; *P. indica* *AMF – *P. indica* and Mycorrhizae treatment.

Fig 12. Relationship between some physiological parameters in inoculated and non-inoculated peppermint with (*P. indica*, AMF and co-inoculation) under salinity stress in peppermint plant. AMF – Mycorrhizae treatment; AMF* *P. indica* –Mycorrhizae and *P. indica* treatment.

Fig 13. Relationship between some metabolites in inoculated and non-inoculated peppermint with (*P. indica*, AMF and co-inoculation) under 9 dsm^{-1} salinity stress in peppermint plant. The salinity and non-salinity condition were indicated in orange and green, respectively.

Fig 14. Leaf metabolites involved in the terpenoied pathways in peppermint plant under salinity stress. The relative abundance of metabolites in inoculated and non-inoculated peppermint with (*P. indica* and AMF) under salt stress was shown as box plots. Regulated metabolites under salinity and non-salinity condition were indicated in orange and green, respectively.

Prof. Mayank Gururani

Editor-in-Chief
PLOS ONE Journal

Dear Prof., Mayank Gururani

Attached please find our revised manuscript entitled: Does co-inoculation of mycorrhiza and Piriformospora indica fungi enhance the efficiency of Chlorophyll fluorescence and essential oil composition in peppermint under irrigation with saline water from the Caspian Sea? (No.: PONE-D-21-02576) for publication in PLOS ONE Journal.

Thank you for giving us this opportunity to revise and resubmit our manuscript. We appreciate your comments and the suggestions made by referees to improve the manuscript. We responded to all of the reviewers' suggestions.

We hope that after these enhancements the manuscript can now be accepted for publication; however, we are certainly willing to consider further changes if necessary.

We look forward to hearing from you.

Yours faithfully,

Masoumeh Khalvandi
In behalf authors

Response to Review Comments

(Manuscript Number: PONE-D-21-02576)

Response to Reviewer #1 Comments

Reviewer point #1: If they limit the discussion to their actual findings, the entire discussion can be summarized to 1/3rd of what is presented.

Author response #1: The length of the discussion part has been reduced. And these sentences were removed in the discussion part.

- The reason that salinity decreased fungal colonization might be attributed to the adverse effects of salinity on photosynthesis, along with a reduction in carbon supply from plant to endophytic fungus. This can also be supported by the fact that salinity induced changes in morphological features of fungal hyphae. Besides,
- These results are similar to what has been recorded in wheat (lyas et al, 2020), and maize (Wang et al, 2020).
- inhibition of QA re-oxidation, and degradation of D1 and D2 proteins in the PSII which
- Salinity stress probably saturates the electron transport chain that leads to accumulation of protons, followed by an increase in the amount of NPQ. The increase in NPQ, which is actually an indicator of heat loss in the processes of the excessive energy removal, shows the high capacity of the xanthophyll cycle (Nilkens et al,. 2010; Murchie and Lawson, 2013).
- Higher values of photochemical activity of PSII in plants which have a symbiotic relationship with fungi are mainly associated with: the increased density of photosynthetic units, the improved reaction center activity, enhancing the plants' ability to use light energy, and facilitating the electron transport to NADP (Xu et al, 2016; Moreira et al. 2015; Zhang et al, 2018; Shahabivand et al, 2017; Ghorbani et al, 2018; Shahabivand et al, 2012). Furthermore, microbial inoculation can alleviate the adverse impacts of salinity on the photochemical activity of PSII through expression of the genes encoding D1 and D2 proteins, improving water absorption, modulating membrane damage, and improving chlorophyll content (Chen et al, 2017).
- AMF has been reported to regulate root permeability by doubling the expression of the LSPLP1 gene, the strategy which leads to a higher tolerance to osmotic stress) (Heikham Evelin et al, 2019).
- In addition to what is mentioned above, salinity suppresses the biosynthesis of essential oil. The reason for the reduction in the synthesis of essential oils can be related to the low availability of assimilates as a result of reduced photosynthetic activity and inactivation of the PSII reaction center (Zuo et al, 2017). However, even when photosynthesis assimilation approaches zero (due to stomatal restriction, damage to chlorophyll, and chloroplast membrane system under severe stress conditions) monoterpenes biosynthesis still occurs due to alternative carbon sources allocation (Lavoir et al, 2009). Naturally, monoterpenes are biosynthesized through MEP/ DOXP

pathway and are fueled by a constant supply of substrates, high amounts of acetyl-CoA, and ATP and NADPH+H cofactors (Niinemets et al., 2002, Behn et al, 2010). This can be the reason for the tight link between methyl erythritol-4-osphate (MEP) and photosynthesis, as the Calvin cycle in chloroplasts is the main carbon sources for terpenoid biosynthesis and the MEP pathway is a place for reducing excess electron flow in the photosynthetic electron transfer chain (Valifard et al, 2018).

- It is believed that the monoterpene pattern is mostly determined by NADPH+H+ -dependent reductive interconversions, like a reduction of menthone to menthol (Behn et al, 2010). These modifications in the biosynthesis of monoterpenes can be linked to alterations in the photosynthetic electron transfer chain, the production of photosynthetic NDPH2 (Maffei and Codignola, 1990), and oxidation of monoterpenes by ROS to generate oxygenated forms under environmental stress (Zuo et al, 2017).

These references were also removed in the revised manuscript.

1. Agron. 10, 160–162.Wu, M., Wei, Q., Xu, L., Li, H., Oelmüller, R., & Zhang, W. (2018). Piriformospora indica enhances phosphorus absorption by stimulating acid phosphatase activities and organic acid accumulation in Brassica napus. Plant and Soil, 432(1-2), 333-344.
2. Behn, H., Albert, A., Marx, F., Noga, G., & Ulbrich, A. (2010). Ultraviolet-B and photosynthetically active radiation interactively affect yield and pattern of monoterpenes in leaves of peppermint (Mentha× piperita L.). Journal of agricultural and food chemistry, 58(12), 7361-7367.
3. Flexas, J., Escalona, J. M., & Medrano, H. (1999). Water stress induces different levels of photosynthesis and electron transport rate regulation in grapevines. Plant, Cell & Environment, 22(1), 39-48.
4. lyas, N., Mazhar, R., Yasmin, H., Khan, W., Iqbal, S., Enshasy, H. E., & Dailin, D. J. (2020). Rhizobacteria isolated from saline soil induce systemic tolerance in wheat (Triticum aestivum L.) against salinity stress. Agronomy, 10(7), 989.
5. Lavoir, A. V., Staudt, M., Schnitzler, J. P., Landais, D., Massol, F., Rocheteau, A., ... & Rambal, S. (2009). Drought reduced monoterpene emissions from the evergreen Mediterranean oak Quercus ilex: results from a throughfall displacement experiment. Biogeosciences, 6(7), 1167-1180.
6. Maffei, M., & Codignola, A. (1990). Photosynthesis, photorespiration and herbicide effect on terpene production in peppermint (Mentha piperita L.). Journal of Essential Oil Research, 2(6), 275-286.
7. Sebastian, A., & Prasad, M. N. V. (2019). Photosynthetic light reactions in Oryza sativa L. under Cd stress: Influence of iron, calcium, and zinc supplements. The EuroBiotech Journal. 3(4): 175-181.
8. Zuo, Z., Wang, B., Ying, B., Zhou, L., & Zhang, R. (2017). Monoterpene emissions contribute to thermotolerance in Cinnamomum camphora. Trees, 31(6), 1759-1771.

Reviewer point #2: Abstract: First sentence of the abstracts seems complex and needs to be simplified

Author response #2: The correction was done in Abstract.

Reviewer point #3: Introduction – 2nd paragraph – 3rd line: 'Photosynthesis process' – please delete 'process'

Author response #3: process was deleted in 'Photosynthesis process'

Reviewer point #4: Introduction – 4th paragraph – Line No.12: 'which are all pharmaceutical useful' – please modify as 'pharmaceutically useful'

Author response #4: 'which are all pharmaceutical useful' was modified to 'pharmaceutically useful'

Reviewer point #5: Material and Methods: 1st paragraph – 2nd line: 'This research was carried out in a factorial experiment in a….' – may be modified as: 'A factorial experiment was carried out in a....'

Author response #5: 'This research was carried out in a factorial experiment in a….' was modified to 'A factorial experiment was carried out in a....'

Reviewer point #6: Discussion – 9th paragraph: 'Several reports have reported a direct correlation between nutrient imbalance and the simultaneous decline in PSII function in salt-stressed plants' - the sentence may be modified as 'Several reports suggest a direct correlation between nutrient imbalance and the simultaneous decline in PSII function in salt-stressed plants'

Author response #6: The correction has been made in the revised manuscript.

Reviewer point #7: which are these reports? Please refer to the reports; the reference of Yang et al. (2021) is not sufficient to substantiate the argument of 'several reports'; otherwise, modify the sentence.

Author response #7: Thank you for pointing this out. The following articles were added to the references.

- Loudari, A., Benadis, C., Naciri, R., Soulaimani, A., Zeroual, Y., Gharous, M. E., Kalaji, H M., & Oukarroum, A. (2020). Salt stress affects mineral nutrition in shoots and roots and chlorophyll a fluorescence of tomato plants grown in hydroponic culture. Journal of Plant Interactions, 15(1), 398-405.

- Qu, C., Liu, C., Gong, X., Li, C., Hong, M., Wang, L., & Hong, F. (2012). Impairment of maize seedling photosynthesis caused by a combination of potassium deficiency and salt stress. Environmental and Experimental Botany, 75, 134-141

Reviewer point #8: 9th paragraph – line 7: 'fungi symbiosis' – may be modified as fungal symbiosis

Author response #8: 'fungi symbiosis' was modified to 'fungal symbiosis'

Reviewer point #9: 9th paragraph: lines 6-7: 'Nevertheless, endophytic fungi symbiosis mitigated the inhibitory effect of salt stress on the mineral uptake' – please mention the evidence which the authors have presented as results in their study to substantiate this argument; otherwise delete this speculative argument. (the first sentence of this paragraph is 'The results of this study showed that severe salinity stress remarkably reduced P and K+ uptake because of the high rate of Na+ absorption', is contradictory to this argument.

Author response #9: 'Nevertheless, endophytic fungi symbiosis mitigated the inhibitory effect of salt stress on the mineral uptake' was modified to 'Nevertheless, in inoculated plant endophytic fungal symbiosis increased P and K+ content (Figure. 6a) and decreased Na+ uptake in peppermint leaves (Figure. 4c).'

In order to avoid ambiguity and contradiction, 'in non-inoculated plant' and 'in inoculated plant' were added to the text of this paragraph.

Reviewer point #10: Line 11: 'osmotic stress)' – delete the bracket

Author response #10**:** done

Reviewer point #11: 10th paragraph: lines 5-7: 'In agreement with the present results, Xu et al. (2016) reported that the positive effect of AMF on Fv/Fm increasing in maize seedlings can be attributed to the absorption of mineral nutrients, activating mediated genes and sink stimulation' – how can it be in agreement with, especially when the authors argue in the 9th paragraph that 'The results of this study showed that severe salinity stress remarkably reduced P and K+ uptake because of the high rate of Na+ absorption' ? - please note the contradiction and revise

Author response #11:

In order to avoid ambiguity and contradiction, 'The results of this study showed that severe salinity stress remarkably reduced P and K+ uptake because of the high rate of Na+ absorption' was modified to 'The results of this study showed that severe salinity stress remarkably reduced P and K^+ uptake in non-inoculated plant because of the high rate of Na^+ absorption.'

Thank you for your valuable suggestion. After reviewing articles in various journals, we found that they also reported such contradictions, and in all of them, although the rate of colonization and formation of fungal hyphae and spores decreased under salinity stress, its presence in the plant roots was able to reduce the negative effects of salinity stress on the plant and improves the amount of some plant parameters.

- Klinsukon, C., Lumyong, S., Kuyper, T. W., & Boonlue, S. (2021). Colonization by arbuscular mycorrhizal fungi improves salinity tolerance of eucalyptus (Eucalyptus camaldulensis) seedlings. Scientific Reports, 11(1), 1-10.

- Zai, X. M., Fan, J. J., Hao, Z. P., Liu, X. M., & Zhang, W. X. (2021). Effect of co-inoculation with arbuscular mycorrhizal fungi and phosphate solubilizing fungi on nutrient uptake and photosynthesis of beach palm under salt stress environment. Scientific Reports, 11(1), 1-11.

- Parvin, S., Van Geel, M., Yeasmin, T., Verbruggen, E., & Honnay, O. (2020). Effects of single and multiple species inocula of arbuscular mycorrhizal fungi on the salinity tolerance of a Bangladeshi rice (Oryza sativa L.) cultivar. Mycorrhiza, 30(4), 431-444.

- Kaya, C., Ashraf, M., Sonmez, O., Aydemir, S., Tuna, A. L., & Cullu, M. A. (2009). The influence of arbuscular mycorrhizal colonisation on key growth parameters and fruit yield of pepper plants grown at high salinity. Scientia horticulturae, 121(1), 1-6.

- Jahromi, F., Aroca, R., Porcel, R., & Ruiz-Lozano, J. M. (2008). Influence of salinity on the in vitro development of Glomus intraradices and on the in vivo physiological and molecular responses of mycorrhizal lettuce plants. Microbial Ecology, 55(1), 45.

Reviewer point #12: I think the 11th and 12th paragraph are unnecessary as these do not discuss any specific findings of the researchers.

Author response #12: These 11th and 12th paragraphs was delated in the revised manuscript.
