# Supplementary material for: Does co-inoculation of mycorrhiza and Piriformospora indica fungi enhance the efficiency of chlorophyll fluorescence and essential oil composition in peppermint under irrigation with saline water from the Caspian Sea?
Source: PLoS One. 2021 Jul 9;16(7):e0254076. doi: 10.1371/journal.pone.0254076 (PMC8270468; doi:10.1371/journal.pone.0254076)
Supplement: Attachment — Submitted filename: Response to Reviewers.docx [file pone.0254076.s006.docx]

Prof. Mayank Gururani

Editor-in-Chief
PLOS ONE Journal

Dear Prof., Mayank Gururani

Attached please find our revised manuscript entitled: Does co-inoculation of mycorrhiza and Piriformospora indica fungi enhance the efficiency of Chlorophyll fluorescence and essential oil composition in peppermint under irrigation with saline water from the Caspian Sea? (No.: PONE-D-21-02576) for publication in PLOS ONE Journal.

Thank you for giving us this opportunity to revise and resubmit our manuscript. We appreciate your comments and the suggestions made by referees to improve the manuscript. We responded to all of the reviewers' suggestions.

We hope that after these enhancements the manuscript can now be accepted for publication; however, we are certainly willing to consider further changes if necessary.

We look forward to hearing from you.

Yours faithfully,

Masoumeh Khalvandi

In behalf authors

**Response to Review Comments**

**(Manuscript Number: PONE-D-21-02576)**

***Response to Reviewer #1 Comments***

***Reviewer point #1: Page 1: Introduction: line 1-2: “which its demand has grown dramatically in recent decades” – may be corrected as: ‘the demand of which has grown dramatically in recent decades’***

***Author response #1:*** the correction has been made in the revised manuscript.

***Reviewer point #2: Line 3: “It is evident that peppermint essential oil” – may be modified as: ‘it is evident that the essential oil’***

***Author response #2:*** “It is evident that peppermint essential oil” was modified to ‘it is evident that the essential oil’

***Reviewer point #3: Line 5-6: “as well as the reactive oxygen species (ROS) scavenging activity because of its valuable constituents” – ‘as well as scavenging of reactive oxygen species (ROS)’***

***Author response #3:*** This has been corrected in the revised manuscript.

***Reviewer point #4:*** ***2nd Para – line 1: - avoid ‘Nowadays’; the sentence may begin as ‘The sustainability …’***

***Author response #4***: (Nowadays) was removed in the introduction.

***Reviewer point #5:*** ***Line 5: no need of repeating – ‘reactive oxygen species (ROS)’, better put the short form only – ROS because the same is already defined above***

***Author response #5:*** done

***Reviewer point #6:*** ***Line 7: “stresses; it can show’ – ‘stresses, because the same can show’***

***Author response #6:*** “stresses; it can show’ was modified to ‘stresses, because the same can show’

***Reviewer point #7:*** ***Page 3: 2nd para – 2nd line: not ‘sever’ – but ‘severe’***

***Author response #7:*** ‘sever’ was changed to ‘severe’

***Reviewer point #8:*** ***Page 4: para 3- line 2: not ‘fungi symbiosis’ – ‘fungal symbiosis’***

***Author response #8:*** the correction has been made in the revised manuscript. ‘fungi symbiosis’ was modified to ‘fungal symbiosis’

***Reviewer point #9: Discussion:*** ***Page 4-5: line 1-4: “In the present study, the root colonization with P. indica and AMF were remarkably reduced by increasing salinity (6 to 9 dSm−1). Similar observations were reported in other plants (Wu et al., 2010; Hadian-Deljou, et al, 2020; khalvandi et al, 2019). The reason that salinity decreased fungal colonization might be attributed to the adverse 5 effects of salinity on photosynthesis, along with a reduction in carbon supply” The above statements seem contradictory to the claim below on page 5, para 5 – line 1-4: “Our findings showed that P. indica, AMF and their co-inoculation symbiosis mitigated the inhibitory effect of salinity on the photosynthetic capability of peppermint”***

***Reviewer point #10: Last para – line 1-2: “The endophytic fungus can mitigate the toxic influence of salinity on chloroplast and chlorophyll by releasing cytokinin-like substances which maintain the relative stability of the organelle” – how can the fungi mitigate the toxic influence, when the fungi themselves are negatively affected by salinity?
“Nevertheless, endophytic fungi symbiosis mitigated the inhibitory effect of salt stress on the mineral uptake. It has been well documented that endophytic fungi can prevent the toxicity of Na+ in aerial parts of plants through accumulation of Na+ ions in fungal cytosol, hyphae wall and vesicles” – more explanations are required for this argument, especially when the fungi are harmfully affected by salt stress***

***Author response #9 & 10:***

In this experiment, symbiotic relationship with fungi under no stress and mild stress conditions had a positive significant effect on the examined traits. On the other hand, we observed negative effects of high salinity levels on colonization and physiological parameters of peppermint plants. Although, fungi themselves are negatively affected by salinity, but the results showed that compared to the control plants, symbiotic relationship with these fungi improved the photosynthetic apparatus function. Based on the reports of some researchers, these changes in the presence of fungi and under severe salinity stress can be due to:

The participating AMF induces expression of genes involved in N+ extrusion to the soil solution, K+ acquisition (by phloem loading and unloading), and release into the xylem, therefore maintaining a favorable Na: K ratio. Colonization by AMF differentially affects the expression of the plasma membrane and tonoplast aquaporins (PIPs and TIPs), which consequently improves the water status of the plant. The formation of AM (arbuscular mycorrhiza) surges the capacity of the plant to mend photosystem-II (PSII) and boosts the quantum efficiency of PSII under salt stress conditions by mounting the transcript levels of chloroplast genes encoding antenna proteins involved in the transfer of excitation energy. Furthermore, AM-induced interplay of phytohormones, including strigolactones, abscisic acid, gibberellic acid, salicylic acid, and jasmonic acid have also been associated with the salt tolerance mechanism (Heikham Evelin et al, 2019).

***Reviewer point #11:*** ***Overall, the researchers have observed evidence for synergistic effect Pirimorphospora and AM in alleviating salt stress in peppermint; however, their arguments need more explanations to avoid self-contradictory appearance as they found salt stress also negatively affecting the plant metabolism as well as fungal colonization. Although, they provide evidences of previous positive findings of synergistic influence of AM and Pirimorphospora in certain plants, they need to report findings showing no such influences as well ( for example: DOI: 10.1080/01904160903435409 )***

***Author response #11: The correction was made in Conclusion section.***

***Response to Reviewer #2 Comments***

***Reviewer point #1: [Introduction] Piriformospora indica, also named as Sebacinales indica, the author needs to add it.***

***Author response #1:*** "Sebacinales indica" was added to the text

***Reviewer point #2: [Materials and Methods] Give more specific info on inoculum: e.g. mycelia mass or chlamydospores per ml of P. indica and AMF. And provide the method or reference of fungi inoculation and detection.***

***Author response #2:*** The methods of fungi inoculation and amount of AMF and P. indica spores were added to the Materials and Methods.

***Reviewer point #3: [Results] This study has many figure, but author doesn’t or good explain the result of the figure.***

***Author response #3:*** Thank you for pointing this out. The reviewer is correct, we checked all figures and we found in Fv/fm some mistake was made because of careless writing. This has been corrected in the revised manuscript.

Also, Analysis of variance was added to the Results.

Figures 7, 10, and 12 were referred to the text.

***Reviewer point #4:*** ***[Discussion] The conclusions drawn by the authors that P. indica, AMF and their co-inoculation symbiosis mitigated the inhibitory effect of salinity on the photosynthetic capability of peppermint is not really supported by those parameters. The authors mention that higher photosynthetic capability attributed to the better physiological status. Why the author not measure the ROS scavengers related enzyme activity to support the point? Kindly explain.***

***Author response #4:*** Thank you for your suggestion. We studied the antioxidant enzymes, such as catalase and superoxide dismutase, and we have considered it for another article. But if necessary, we can also represent them.

***Reviewer point #5:*** ***The Fig. 2 was not clear, please replace it and provide the scale or microscope magnification. The microscopic observation*** ***pictures of the control plant roots are also need provided. “chlamidospore” probably “chlamydospore”? Please check it.***

***Author response #5:*** the scale or microscope magnification was (10- 40X). the scale was added to the pictures. We separated the pictures to make the pictures clearer.

“chlamidospore” was modified to “chlamydospore

***Reviewer point #6:*** ***Fig. 3, add the abbreviation of Pi. Give the test sample number of root colonization of each group.***

***Author response #6:*** The correction was done in text.

***Reviewer point #7:*** ***All the column figures in this study have no error bars, please check/add it.***

***Author response #7:*** Error bars was added to the all figures.

***Reviewer point #8:*** ***Fig. 5 (a-c) different with other column figure like Fig. 4, please check/replace it.***

***Author response #8:*** Thank you for pointing this out. The reviewer is correct, this mistake was made because of using the wrong way of lettering Means. We learned from one of our colleagues that there is a more accurate way to Lettering means. then, we did the Lettering method through the SAS program itself, and all figures have been corrected.

***Reviewer point #9: Fig. 6, “AMF* Pi” or “Pi *AMF”? “PI” or “Pi”? Same with Fig. 7, 8, 10, 12 and 13, keep one unified format. please check/replace it.***

***Author response #9:*** The reviewer is correct; the correction has been made in the revised manuscript.

***Reviewer point #10: I suggest the authors provide an intuitive picture of each treatment group sample peppermint to show the result of this study.***

***Author response #10:*** Thank you for your valuable suggestion. This research has already been done and we only have some pictures from the time of planting that we present.

***Reviewer point #11: Here two excellent publications on symbiosis of P. indica and Mentha piperita should be read in this study to give some reference. Dolatabadi, H.K., Goltapeh, E.M., Moieni, A., Varma, A., 2012. Evaluation of different densities of auxin and endophytic fungi (Piriformospora indica and Sebacina vermifera) on Mentha piperita and Thymus vulgaris growth. Afr J Biotechnol, 11: 1644–1650.
Dolatabadi, H.K., Goltapeh, E.M., Safari, M., Golafaie, T.P., 2017. Potential effect of Piriformospora indica on plant growth and essential oil yield in Mentha piperita. Plant Pathol Quar, 7: 96–104.***

***Author response #11:*** These references were added to the revised manuscript.

***Response to Reviewer #3 Comments***

***Reviewer point #1: 1st Para- line 1:- ‘Which its’ can be written as ‘and its’***

***Author response #1:*** ‘Which its’ was modified to ‘and its’

***Reviewer point #2: 2nd Para- line 1:- Yield is negatively affected (Kindly add references)***

***Author response #2:*** The appropriate reference was added to "Yield is negatively affected"

***Reviewer point #3: 3rd Para- 2nd last line:- ‘Quercus ilex’ should be italics***

***Author response #3:*** The correction was done in text.

***Reviewer point #4:*** ***1st para- line 3:- ‘Three replications’; always use minimum five replications***

***Author response #4:*** Thanks to the referee’s suggestion, definitely conducting the experiment with five replications would be more accurate. However, we found authoritative articles that tested their experiment with three repetitions, such as:

1. Bharti, N., Pandey, S. S., Barnawal, D., Patel, V. K., & Kalra, A. (2016). Plant growth promoting rhizobacteria Dietzia natronolimnaea modulates the expression of stress responsive genes providing protection of wheat from salinity stress. Scientific reports, 6(1), 1-16.
2. Gururani, M. A., Venkatesh, J., Ghosh, R., Strasser, R. J., Ponpandian, L. N., & Bae, H. (2018). Chlorophyll-a fluorescence evaluation of PEG-induced osmotic stress on PSII activity in Arabidopsis plants expressing SIP1. Plant Biosystems-An International Journal Dealing with all Aspects of Plant Biology, 152(5), 945-952.
3. Li, X., Zhao, C., Zhang, T., Wang, G., Amombo, E., Xie, Y., & Fu, J. (2021). Exogenous Aspergillus aculeatus Enhances Drought and Heat Tolerance of Perennial Ryegrass. *Frontiers in Microbiology*, *12*, 307.
4. Varghese, N., Alyammahi, O., Nasreddine, S., Alhassani, A., & Gururani, M. A. (2019). Melatonin positively influences the photosynthetic machinery and antioxidant system of Avena sativa during salinity stress. Plants, 8(12), 610.
5. Yamane, K., Oi, T., Enomoto, S., Nakao, T., Arai, S., Miyake, H., & Taniguchi, M. (2018). Three‐dimensional ultrastructure of chloroplast pockets formed under salinity stress. Plant, cell & environment, 41(3), 563-575.
6. Ji, J., Yue, J., Xie, T., Chen, W., Du, C., Chang, E., ... & Shi, S. (2018). Roles of γ-aminobutyric acid on salinity-responsive genes at transcriptomic level in poplar: involving in abscisic acid and ethylene-signalling pathways. Planta, 248(3), 675-690.

***Reviewer point #5:*** ***Line 5:- Kindly mention the spore counts of P indica and AMF***

***Author response #5:*** 10 g of Arbuscular mycorrhizal inoculum (with a density of 120 active spores per gram) and 10 ml of Piriformospora indica suspension (1× 10^9^) were added to the pots.

***Reviewer point #6:*** ***Line 6:- Kindly specify the ratio of Caspian sea water and distilled water***

***Author response #6:*** In order to reach each level of salinity stress: determined amount of the Caspian Sea water and distilled water were mixed and then the desired salinity was determined by EC meter.

***Reviewer point #7:*** ***Physiological parameters: Line 3-5:- Kindly elaborate the methods used for analysis***

***Author response #7:*** The methods used for analysis were added to the Materials and Methods section.

Chlorophyll fluorescence parameters were measured in the last fully developed leaf by using pulse amplitude modulated fluorometer (PAM-2500, Walz, Germany). First, leaves were placed in darkness for 30 minutes using specific leaf clamps. The samples were exposed to low-intensity light [< 0.1 μmol (photon) m−2 s−1, red light]. Then, a saturating light pulse [> 8,000 μmol (photon) m−2 s−1, white light) was turned on for 1 s (one pulse). The minimum fluorescence (Fo) and maximum fluorescence (Fm) were determined in dark-adapted leaves. The variable fluorescence (Fv) and maximum quantum photosystem II efficiency (Fv/Fm) were evaluated based on Equations 1 and 2. and the effective photochemical quantum efficiency II [ФPSII], electron transfer rate [ETR, μmol(electron) m–2 s–1), non-photochemical quenching (NPQ), the quantum yield of regulated energy dissipation (YNPQ), and Quantum yield of non-regulated energy dissipation in PSII (Y (NO)) were calculated by using Equations 3 and 6 (Khanghahi et al, 2012).

1) Fv= Fm- Fo

2) Fv/Fm= (Fm- Fo)/ Fm

3) ФPSII= (Fm´ − F)/Fm´

4) NPQ = (Fm/Fm') – 1

5) Y(NPQ) = (F/Fm') – (F/Fm)

6) Y(NO) = F/Fm

F= steady-state fluorescenc

Fm'= maximum fluorescence measured in light-exposed leaf samples.

***Reviewer point #8:*** ***Results: 1st Para- 2nd last line:- Kindly*** ***confirm the synergistic association between P indica & AMF through plating techniques.***

***Author response #8: Thank you for your valuable suggestion, we definitely investigate the synergistic association between P indica & AMF through plating techniques in our future research; however, this research has already been conducted and done; thus, we can't add a new experiment.***
