# Supplementary material for: Does co-inoculation of mycorrhiza and Piriformospora indica fungi enhance the efficiency of chlorophyll fluorescence and essential oil composition in peppermint under irrigation with saline water from the Caspian Sea?
Source: PLoS One. 2021 Jul 9;16(7):e0254076. doi: 10.1371/journal.pone.0254076 (PMC8270468; doi:10.1371/journal.pone.0254076)
Supplement: Attachment — Submitted filename: Response to Reviewers.docx [file pone.0254076.s007.docx]

Prof. Mayank Gururani

Editor-in-Chief
PLOS ONE Journal

Dear Prof., Mayank Gururani

Attached please find our revised manuscript entitled: Does co-inoculation of mycorrhiza and Piriformospora indica fungi enhance the efficiency of Chlorophyll fluorescence and essential oil composition in peppermint under irrigation with saline water from the Caspian Sea? (No.: PONE-D-21-02576) for publication in PLOS ONE Journal.

Thank you for giving us this opportunity to revise and resubmit our manuscript. We appreciate your comments and the suggestions made by referees to improve the manuscript. We responded to all of the reviewers' suggestions.

We hope that after these enhancements the manuscript can now be accepted for publication; however, we are certainly willing to consider further changes if necessary.

We look forward to hearing from you.

Yours faithfully,

Masoumeh Khalvandi

In behalf authors

**Response to Review Comments**

**(Manuscript Number: PONE-D-21-02576)**

***Response to Reviewer #1 Comments***

***Reviewer point #1: If they limit the discussion to their actual findings, the entire discussion can be summarized to 1/3rd of what is presented.***

***Author response #1:*** The length of the discussion part has been reduced. And these sentences were removed in the discussion part.

- The reason that salinity decreased fungal colonization might be attributed to the adverse effects of salinity on photosynthesis, along with a reduction in carbon supply from plant to endophytic fungus. This can also be supported by the fact that salinity induced changes in morphological features of fungal hyphae. Besides,
- These results are similar to what has been recorded in wheat (lyas et al, 2020), and maize (Wang et al, 2020).
- inhibition of QA re-oxidation, and degradation of D1 and D2 proteins in the PSII which
- Salinity stress probably saturates the electron transport chain that leads to accumulation of protons, followed by an increase in the amount of NPQ. The increase in NPQ, which is actually an indicator of heat loss in the processes of the excessive energy removal, shows the high capacity of the xanthophyll cycle (Nilkens et al., 2010; Murchie and Lawson, 2013).
- Higher values of photochemical activity of PSII in plants which have a symbiotic relationship with fungi are mainly associated with: the increased density of photosynthetic units, the improved reaction center activity, enhancing the plants’ ability to use light energy, and facilitating the electron transport to NADP (Xu et al, 2016; Moreira et al. 2015; Zhang et al, 2018; Shahabivand et al, 2017; Ghorbani et al, 2018; Shahabivand et al, 2012). Furthermore, microbial inoculation can alleviate the adverse impacts of salinity on the photochemical activity of PSII through expression of the genes encoding D1 and D2 proteins, improving water absorption, modulating membrane damage, and improving chlorophyll content (Chen et al, 2017).
- AMF has been reported to regulate root permeability by doubling the expression of the LSPLP1 gene, the strategy which leads to a higher tolerance to osmotic stress) (Heikham Evelin et al, 2019).
- In addition to what is mentioned above, salinity suppresses the biosynthesis of essential oil. The reason for the reduction in the synthesis of essential oils can be related to the low availability of assimilates as a result of reduced photosynthetic activity and inactivation of the PSII reaction center (Zuo et al, 2017). However, even when photosynthesis assimilation approaches zero (due to stomatal restriction, damage to chlorophyll, and chloroplast membrane system under severe stress conditions) monoterpenes biosynthesis still occurs due to alternative carbon sources allocation (Lavoir et al, 2009). Naturally, monoterpenes are biosynthesized through MEP/ DOXP pathway and are fueled by a constant supply of substrates, high amounts of acetyl-CoA, and ATP and NADPH+H cofactors (Niinemets et al., 2002, Behn et al, 2010). This can be the reason for the tight link between methyl erythritol-4-osphate (MEP) and photosynthesis, as the Calvin cycle in chloroplasts is the main carbon sources for terpenoid biosynthesis and the MEP pathway is a place for reducing excess electron flow in the photosynthetic electron transfer chain (Valifard et al, 2018).
- It is believed that the monoterpene pattern is mostly determined by NADPH+H+ -dependent reductive interconversions, like a reduction of menthone to menthol (Behn et al, 2010). These modifications in the biosynthesis of monoterpenes can be linked to alterations in the photosynthetic electron transfer chain, the production of photosynthetic NDPH2 (Maffei and Codignola, 1990), and oxidation of monoterpenes by ROS to generate oxygenated forms under environmental stress (Zuo et al, 2017).

These references were also removed in the revised manuscript.

1. Agron. 10, 160–162.Wu, M., Wei, Q., Xu, L., Li, H., Oelmüller, R., & Zhang, W. (2018). Piriformospora indica enhances phosphorus absorption by stimulating acid phosphatase activities and organic acid accumulation in Brassica napus. Plant and Soil, 432(1-2), 333-344.
2. Behn, H., Albert, A., Marx, F., Noga, G., & Ulbrich, A. (2010). Ultraviolet-B and photosynthetically active radiation interactively affect yield and pattern of monoterpenes in leaves of peppermint (Mentha× piperita L.). Journal of agricultural and food chemistry, 58(12), 7361-7367.
3. Flexas, J., Escalona, J. M., & Medrano, H. (1999). Water stress induces different levels of photosynthesis and electron transport rate regulation in grapevines. Plant, Cell & Environment, 22(1), 39-48.
4. lyas, N., Mazhar, R., Yasmin, H., Khan, W., Iqbal, S., Enshasy, H. E., & Dailin, D. J. (2020). Rhizobacteria isolated from saline soil induce systemic tolerance in wheat (Triticum aestivum L.) against salinity stress. Agronomy, 10(7), 989.
5. Lavoir, A. V., Staudt, M., Schnitzler, J. P., Landais, D., Massol, F., Rocheteau, A., ... & Rambal, S. (2009). Drought reduced monoterpene emissions from the evergreen Mediterranean oak Quercus ilex: results from a throughfall displacement experiment. Biogeosciences, 6(7), 1167-1180.
6. Maffei, M., & Codignola, A. (1990). Photosynthesis, photorespiration and herbicide effect on terpene production in peppermint (Mentha piperita L.). Journal of Essential Oil Research, 2(6), 275-286.
7. Sebastian, A., & Prasad, M. N. V. (2019). Photosynthetic light reactions in Oryza sativa L. under Cd stress: Influence of iron, calcium, and zinc supplements. The EuroBiotech Journal. 3(4): 175-181.
8. Zuo, Z., Wang, B., Ying, B., Zhou, L., & Zhang, R. (2017). Monoterpene emissions contribute to thermotolerance in Cinnamomum camphora. Trees, 31(6), 1759-1771.

***Reviewer point #2:*** ***Abstract: First sentence of the abstracts seems complex and needs to be simplified***

***Author response #2:*** The correction was done in Abstract.

***Reviewer point #3:*** ***Introduction – 2nd paragraph – 3rd line: ‘Photosynthesis process’ – please delete ‘process’***

***Author response #3***: process was deleted in ‘Photosynthesis process’

***Reviewer point #4:*** ***Introduction – 4th paragraph – Line No.12: ‘which are all pharmaceutical useful’ – please modify as ‘pharmaceutically useful’***

***Author response #4:*** ‘which are all pharmaceutical useful’ was modified to ‘pharmaceutically useful’

***Reviewer point #5:*** ***Material and Methods: 1st paragraph – 2nd line:*** ***‘This research was carried out in a factorial experiment in a….’ – may be modified as: ‘A factorial experiment was carried out in a....’***

***Author response #5:*** ‘This research was carried out in a factorial experiment in a….’ was modified to ‘A factorial experiment was carried out in a....’

***Reviewer point #6:*** ***Discussion – 9th paragraph: ‘Several reports have reported a direct correlation between nutrient imbalance and the simultaneous decline in PSII function in salt-stressed plants’ - the sentence may be modified as ‘Several reports suggest a direct correlation between nutrient imbalance and the simultaneous decline in PSII function in salt-stressed plants’***

***Author response #6:*** The correction has been made in the revised manuscript.

***Reviewer point #7:*** ***which are these reports? Please refer to the reports; the reference of Yang et al. (2021) is not sufficient to substantiate the argument of ‘several reports’; otherwise, modify the sentence.***

***Author response #7:*** Thank you for pointing this out. The following articles were added to the references.

- Loudari, A., Benadis, C., Naciri, R., Soulaimani, A., Zeroual, Y., Gharous, M. E., Kalaji, H M., & Oukarroum, A. (2020). Salt stress affects mineral nutrition in shoots and roots and chlorophyll a fluorescence of tomato plants grown in hydroponic culture. Journal of Plant Interactions, 15(1), 398-405.
- Qu, C., Liu, C., Gong, X., Li, C., Hong, M., Wang, L., & Hong, F. (2012). Impairment of maize seedling photosynthesis caused by a combination of potassium deficiency and salt stress. Environmental and Experimental Botany, 75, 134-141

***Reviewer point #8: 9th paragraph – line 7: ‘fungi symbiosis’ – may be modified as fungal symbiosis***

***Author response #8***: ‘fungi symbiosis’ was modified to ‘fungal symbiosis’

***Reviewer point #9:*** ***9th paragraph: lines 6-7: ‘Nevertheless, endophytic fungi symbiosis mitigated the inhibitory effect of salt stress on the mineral uptake******’ – please mention the evidence which the authors have presented as results in their study to substantiate this argument; otherwise delete this speculative argument. (the first sentence of this paragraph is ‘The results of this study showed that severe salinity stress remarkably reduced P and K+ uptake because of the high rate of Na+ absorption’, is contradictory to this argument.***

***Author response #9:*** ‘Nevertheless, endophytic fungi symbiosis mitigated the inhibitory effect of salt stress on the mineral uptake’ was modified to ‘Nevertheless, in inoculated plant endophytic fungal symbiosis increased P and K+ content (Figure. 6a) and decreased Na+ uptake in peppermint leaves (Figure. 4c).’

In order to avoid ambiguity and contradiction, ***‘***in non-inoculated plant***’*** and ***‘***in inoculated plant***’*** were added to the text of this paragraph.

***Reviewer point #10:*** ***Line 11: ‘osmotic stress)’ – delete the bracket***

***Author response #10*:** done

***Reviewer point #11: 10th paragraph: lines 5-7: ‘In agreement with the present results, Xu et al. (2016) reported that the positive effect of AMF on Fv/Fm increasing in maize seedlings can be attributed to the absorption of mineral nutrients, activating mediated genes and sink stimulation’ – how can it be in agreement with, especially when the authors argue in the 9th paragraph that ‘The results of this study showed that severe salinity stress remarkably reduced P and K+ uptake because of the high rate of Na+ absorption’ ? - please note the contradiction and revise***

***Author response #11:***

In order to avoid ambiguity and contradiction, ‘The results of this study showed that severe salinity stress remarkably reduced P and K+ uptake because of the high rate of Na+ absorption’ was modified to ‘The results of this study showed that severe salinity stress remarkably reduced P and K^+^ uptake in non-inoculated plant because of the high rate of Na^+^ absorption.’

Thank you for your valuable suggestion. After reviewing articles in various journals, we found that they also reported such contradictions, and in all of them, although the rate of colonization and formation of fungal hyphae and spores decreased under salinity stress, its presence in the plant roots was able to reduce the negative effects of salinity stress on the plant and improves the amount of some plant parameters.

- Klinsukon, C., Lumyong, S., Kuyper, T. W., & Boonlue, S. (2021). Colonization by arbuscular mycorrhizal fungi improves salinity tolerance of eucalyptus (Eucalyptus camaldulensis) seedlings. Scientific Reports, 11(1), 1-10.
- Zai, X. M., Fan, J. J., Hao, Z. P., Liu, X. M., & Zhang, W. X. (2021). Effect of co-inoculation with arbuscular mycorrhizal fungi and phosphate solubilizing fungi on nutrient uptake and photosynthesis of beach palm under salt stress environment. Scientific Reports, 11(1), 1-11.
- Parvin, S., Van Geel, M., Yeasmin, T., Verbruggen, E., & Honnay, O. (2020). Effects of single and multiple species inocula of arbuscular mycorrhizal fungi on the salinity tolerance of a Bangladeshi rice (Oryza sativa L.) cultivar. Mycorrhiza, 30(4), 431-444.
- Kaya, C., Ashraf, M., Sonmez, O., Aydemir, S., Tuna, A. L., & Cullu, M. A. (2009). The influence of arbuscular mycorrhizal colonisation on key growth parameters and fruit yield of pepper plants grown at high salinity. Scientia horticulturae, 121(1), 1-6.
- Jahromi, F., Aroca, R., Porcel, R., & Ruiz-Lozano, J. M. (2008). Influence of salinity on the in vitro development of Glomus intraradices and on the in vivo physiological and molecular responses of mycorrhizal lettuce plants. Microbial Ecology, 55(1), 45.

***Reviewer point #12: I think the 11th and 12th paragraph are unnecessary as these do not discuss any specific findings of the researchers.***

***Author response #12:*** These 11th and 12th paragraphs was delated in the revised manuscript.
