# Supplementary material for: Does co-inoculation of mycorrhiza and Piriformospora indica fungi enhance the efficiency of chlorophyll fluorescence and essential oil composition in peppermint under irrigation with saline water from the Caspian Sea?
Source: PLoS One. 2021 Jul 9;16(7):e0254076. doi: 10.1371/journal.pone.0254076 (PMC8270468; doi:10.1371/journal.pone.0254076)
Supplement: Attachment — Submitted filename: Response to Reviewers.docx [file pone.0254076.s009.docx]

Prof. Mayank Gururani

Editor-in-Chief
PLOS ONE Journal

Dear Prof., Mayank Gururani

Attached please find our revised manuscript entitled: Does co-inoculation of mycorrhiza and *Piriformospora indica* fungi enhance the efficiency of Chlorophyll fluorescence and essential oil composition in peppermint under irrigation with saline water from the Caspian Sea? (No.: PONE-D-21-02576) for publication in PLOS ONE Journal.

Thank you for giving us this opportunity to revise and resubmit our manuscript. We appreciate your comments and the suggestions made by referees to improve the manuscript. We responded to all of the reviewers' suggestions.

We hope that after these enhancements the manuscript can now be accepted for publication; however, we are certainly willing to consider further changes if necessary.

We look forward to hearing from you.

Yours faithfully,

Masoumeh Khalvandi

In behalf authors

**Response to Review Comments**

**(Manuscript Number: PONE-D-21-02576)**

***Response to Reviewer #1 Comments***

***Reviewer point #1: The lengthy and imprecise debate can only tarnish the brilliance of findings in any good research. Therefore, I request the authors to revise the ‘discussion’ to clarify the exact relevance of their findings. Please use the comments given in the ‘discussion’ part of the revised 'manuscript with track changes' as a model for the revision. Please avoid excessive references to previous findings; limit the citations to a maximum of 1-2 most relevant authorities in each case. Please also thoroughly check the entire text before submission of the revision for spelling and grammar; summarize or rephrase sentences to improve clarity wherever necessary. Finally, check the references thoroughly after deletion of unnecessary citations in the text during revision of the text.***

***Author response #1:*** All requested corrections were made in the revised manuscript. The comments made in the ‘discussion’ part of the 'revised manuscript with track changes' were used as a model for revision. citations were reduced to a maximum of 1-2 most relevant authorities in each case. unnecessary references were deleted. The text was grammatically reviewed and corrected.

The following references have been deleted:

1. Calatayud, A., Roca, D., Martínez, P.F., (2006). Spatial-temporal variations in rose leaves under water stress conditions studied by chlorophyll ﬂuorescence imaging. Plant Physiol. Biochem. 44, 564e573
2. Coban, O., Göktürk Baydar, N., (2016). Brassinosteroid effects on some physical and biochemical properties and secondary metabolite accumulation in peppermint (Mentha piperita L.) under salt stress. Ind Crops Prod. 86, 251-258.
3. Gupta, R., Singh, A., Srivastava, M., Singh, V., Gupta, M. M., & Pandey, R. (2017). Microbial modulation of bacoside A biosynthetic pathway and systemic defense mechanism in Bacopa monnieri under Meloidogyne incognita stress. Scientific reports, 7(1), 1-11.
4. Hasan, M., Ma, F., Prodhan, Z., Li, F., Shen, H., Chen, Y., & Wang, X. (2018). Molecular and physio-biochemical characterization of cotton species for assessing drought stress tolerance. International journal of molecular sciences, 19(9), 2636.
5. Khatri, K., & Rathore, M. S. (2019). Photosystem photochemistry, prompt and delayed fluorescence, photosynthetic responses and electron flow in tobacco under drought and salt stress. Photosynthetica, 57(1), 61-74.
6. Kromdijk, J., Głowacka, K., Leonelli, L., Gabilly, S.T., Iwai, M., Niyogi, K.K., Long, S.P. (2016). Improving photosynthesis and crop productivity by accelerating recovery from photoprotection. Science 354, 857–861.
7. Kumar, G. (2020). Unit 7 Topic: Arbuscular Mycorrhizal Colonization In Plant Roots.
8. Li, D., Liu, H., Qiao, Y., Wang, Y., Cai, Z., Dong, B., Shi, C., Liu, Y., Li, X., and Liu, M. (2013). Effects of elevated CO2 on the growth, seed yield, and water use efficiency of soybean (Glycine max (L.) Merr.) under drought stress. Agricultural Water Management, 129, 105-112.
9. Li, Y., Song, H., Zhou, L., Xu, Z., & Zhou, G. (2019). Tracking chlorophyll fluorescence as an indicator of drought and rewatering across the entire leaf lifespan in a maize field. Agricultural Water Management, 211, 190-201.
10. Loomis, W.D., Corteau, R., 1972. Essential oil biosynthesis. Recent Adv Phytochem. 6, 147-185.
11. McMillen, B.G., Juniper, S., Abbott, L.K., (1998). Inhibition of hyphal growth of a Vesicular arbuscular mycorrhizal fungus in soil containing sodium chloride limits the spread of infection from spores. Soil Biol. Biochem. 30, 1639–1646.
12. Moreira, B. C., Junior, P. P., Jordao, T. C., da Silva, M. D. C. S., Stürmer, S. L., Salomão, L. C. C., ... & Kasuya, M. C. M. (2016). Effect of inoculation of symbiotic fungi on the growth and antioxidant enzymes’ activities in the presence of Fusarium subglutinans f. sp. ananas in pineapple plantlets. Acta Physiologiae Plantarum, 38(10), 1-14.
13. Niinemets, Ü., Hauff, K., Bertin, N., Tenhunen, J. D., Steinbrecher, R., & Seufert, G. (2002). Monoterpene emissions in relation to foliar photosynthetic and structural variables in Mediterranean evergreen Quercus species. New Phytologist, 153(2), 243-256.
14. Nilkens, M., Kress, E., Lambrev, P., Miloslavina, Y., Müller, M., Holzwarth, A.R., Jahns, P. (2010). Identification of a slowly inducible zeaxanthin-dependent component of nonphotochemical quenching of chlorophyll fluorescence generated under steady-state conditions in Arabidopsis.
15. Ormeño, E., Olivier, R., Mévy, J. P., Baldy, V., & Fernandez, C. (2009). Compost may affect volatile and semi-volatile plant emissions through nitrogen supply and chlorophyll fluorescence. Chemosphere, 77(1), 94-104.
16. Rai, M., Acharya, D., Singh, A., Varma, A., (2001). Positive	growth	responses	of	the medicinal	plants Spilanthes calva and Withania somnifera to inoculation by Piriformospora indica in a field trial. Mycorrhiza. 11, 123-128.
17. Rapparini, F., Llusià, J., & Peñuelas, J. (2008). Effect of arbuscular mycorrhizal (AM) colonization on terpene emission and content of Artemisia annua L. Plant Biology, 10(1), 108-122.
18. Shahabivand, S., Parvaneh, A., & Aliloo, A. A. (2017). Root endophytic fungus Piriformospora indica affected growth, cadmium partitioning and chlorophyll fluorescence of sunflower under cadmium toxicity. Ecotoxicology and environmental safety, 145, 496-502.
19. Sun, C., Johnson, J. M., Cai, D., Sherameti, I., Oelmüller, R., & Lou, B. (2010). Piriformospora indica confers drought tolerance in Chinese cabbage leaves by stimulating antioxidant enzymes, the expression of drought-related genes and the plastid-localized CAS protein. Journal of plant physiology, 167(12), 1009-1017.
20. Tarraf, W., Ruta, C., Cillis, F.D., Tagarelli, A., Tedone, L., Mastro, G.D., (2015). Effects of mycorrhiza on growth and essential oil production in selected aromatic plants. J.
21. Vickers, C. E., Gershenzon, J., Lerdau, M. T., & Loreto, F. (2009). A unified mechanism of action for volatile isoprenoids in plant abiotic stress. Nature chemical biology, 5(5), 283.
22. Tsai, M.L., Wu, C.T., Lin, T.F., Lin, W.C., Huang, Y.C., Yang, C.H., (2013). Chemical composition and biological properties of essential oils of two mint species. Trop. J. Pharm. Res. 12, 577–582.

***Reviewer #4: The authors have adequately addressed the points raised by the previous reviewers and the manuscript can be accepted for publication. However, the article will look more authentic if you could mention the source of both the Piriformospora indica and Arbuscular mycorrhizal fungi used in this work. If available, mention the culture depository details as well.***

***Author response #1:*** The source of both the *Piriformospora indica* and Arbuscular mycorrhizal fungi used in this work was added in text.

We obtained both he *Piriformospora indica* and Arbuscular mycorrhizal fungi from Sari Agricultural Sciences and Natural Resources University. But the main source of these two fungi was as follows: Mycorrhiza fungi inoculum (consisted of spores in a sand and mycorrhizal roots mixture) was prepared from Turan Biotechnology Company, Shahrood, Iran. The Piriformospora indica culture was kindly gifted by Prof. Karl-Heinz Kogel, Institute of Phytopathology and Applied Zoology, University of Giessen, Germany. P. indica was cultured in liquid Kafer’s medium at 24◦C for 10 days (Yaghoubian et al., 2019).

***Journal Requirements:***

***Please review your*** ***reference list to ensure that it is complete and correct. If you have cited papers that have been retracted, please include the rationale for doing so in the manuscript text, or remove these references and replace them with relevant current references. Any changes to the reference list should be mentioned in the rebuttal letter that accompanies your revised manuscript. If you need to cite a retracted article, indicate the article’s retracted status in the References list and also include a citation and full reference for the retraction notice.***

***Author response #1:*** reference list has been corrected in the revised manuscript.

"Arnon, D. I. (1949). Copper enzymes in isolated chloroplasts. Polyphenol oxidase in Beta vulgaris. Plant physiology, 24(1), 1" was replaced with "Hameed, A., Akram, N. A., Saleem, M. H., Ashraf, M., Ahmed, S., Ali, S., ... & Alyemeni, M. N. (2021). Seed treatment with α-tocopherol regulates growth and key physio-biochemical attributes in carrot (Daucus carota l.) plants under water limited regimes. Agronomy, 11(3), 469".

"Hemming, D., (2013). Plant sciences reviews 2012. CABI. 280 pp. (online ISSN 1749-8848)" was replaced with " Sun, Z., Wang, H., Wang, J., Zhou, L., Yang, P., (2014). Chemical composition and anti-inflammatory, cytotoxic and antioxidant activities of essential oil from Leaves of Mentha piperita grown in china. PLoS One. 12, e114767."

"Bertamini, M., Faralli, M., Varotto, C., Grando, M. S., & Cappellin, L. (2021). Leaf Monoterpene Emission Limits Photosynthetic Downregulation under Heat Stress in Field-Grown Grapevine. Plants, 10(1), 181" was corrected.
