# Supplementary material for: Does co-inoculation of mycorrhiza and Piriformospora indica fungi enhance the efficiency of chlorophyll fluorescence and essential oil composition in peppermint under irrigation with saline water from the Caspian Sea?
Source: PLoS One. 2021 Jul 9;16(7):e0254076. doi: 10.1371/journal.pone.0254076 (PMC8270468; doi:10.1371/journal.pone.0254076)
Supplement: Attachment — Submitted filename: Reviewer Comments PLOS ONE.doc [file pone.0254076.s005.doc]

**Reviewer Comments**

**Introduction:**

1^st^ Para- line 1:- ‘Which its’ can be written as ‘and its’

2^nd^ Para- line 1:- Yield is negatively affected (Kindly add references)

3^rd^ Para- 2^nd^ last line:- ‘*Quercus ilex’* should be italics

**Material and Methods**

Growth Conditions:-

1^st^ para- line 3:- ‘Three replications’; always use minimum five replications

Line 5:- Kindly mention the spore counts of *P indica* and *AMF*

Line 6:- Kindly specify the ratio of Caspian sea water and distilled water

Physiological parameters:

Line 3-5:- Kindly elaborate the methods used for analysis

**Results:**

1^st^ Para- 2^nd^ last line:- Kindly confirm the synergistic association between P indica & AMF through plating techniques.
